# Cyclin-dependent kinase inhibitor 1A inhibits pyroptosis to enhance human lung adenocarcinoma cell radioresistance by promoting DNA repair

**DOI:** 10.1016/j.heliyon.2024.e26975

**Published:** 2024-02-29

**Authors:** Jing Li, Teng Liu, Ning Tang, Sheng Lin, Feng Zhang, Wei Yuan, Ting Zhang, Shi-hua Deng, Dong-ming Wu, Ying Xu

**Affiliations:** a School of Clinical Medicine, Chengdu Medical College, Chengdu, Sichuan, 610500, PR China; b The First Affiliated Hospital of Chengdu Medical College, Chengdu, Sichuan, 610500, PR China; c The First People's Hospital of Ziyang City, Ziyang, Sichuan, PR China

**Keywords:** CDKN1A, Pyroptosis, Radiotherapy, DNA repair, Inflammasomes

## Abstract

**Purpose:**

One of the best anticancer treatments available is radiotherapy, which can be used either alone or in conjunction with other forms of treatment including chemotherapy and surgery. Nevertheless, a number of biochemical and physiological processes that react to ionizing radiation might provide tumor cells radioresistance, which makes radiotherapy ineffective. It has been found that CDKN1A regulates DNA damage repair, which contributes to tumor radioresistance. However, the precise mechanism is still unknown. Therefore, this study aimed to explore the mechanisms underlying CDKN1A-enhanced radioresistance in tumor cells.

**Methods:**

Cells were irradiated with 4 Gy after CDKN1A overexpression or knockdown. CDKN1A expression was measured using real-time PCR, cell viability was evaluated using cell counting kit-8 and colony formation assays, and cytotoxicity was assessed using a lactate dehydrogenase assay. Pyroptosis in cells was analyzed using caspase-1 activity assay, enzyme-linked immunosorbent assay, and flow cytometry. Inflammation activation was detected through a co-immunoprecipitation assay. Activation of pyroptosis-related proteins was analyzed using immunohistochemistry, Western blot, and immunofluorescence. Tumor radioresistance *in vivo* was evaluated in a mouse xenograft model.

**Results:**

Radiotherapy upregulated CDKN1A expression, which promoted lung adenocarcinoma cell survival. CDKN1A influenced radiation-induced pyroptosis in A549, which mainly depended on inhibiting the activation of the AIM2 inflammasome by promoting DNA repair. Additionally, CDKN1A upregulation enhanced A549 xenograft tumor radioresistance by inhibiting radiation-induced pyroptosis *in vivo*.

**Conclusions:**

CDKN1A inhibits pyroptosis to enhance the radioresistance of lung adenocarcinoma cells by promoting DNA repair. This study may serve as a reference for developing novel targeted therapies against cancer.

## Highlights

• CDKN1A confers radioresistance to A549 and H1650 human lung adenocarcinoma cells.
• CDKN1A influences radiation-induced pyroptosis in A549 cells and xenograft tumors.
• CDKN1A inhibits AIM2 inflammasome activation by promoting DNA repair.

## 1 Introduction

Non-small cell lung cancer accounts for over 85% of all human lung cancers, with lung adenocarcinoma (LUAD) being the most common subtype, accounting for half of all occurrences. Radiotherapy is the principal treatment for lung cancer. Approximately 50% of patients with cancer undergo radiotherapy [1] for curative or palliative purposes. Despite the great progress in radiotherapy in recent years, the application of this treatment is limited by tumor cell resistance, which leads to unsatisfactory treatment outcomes or disease recurrence [2]. Several cellular and molecular mechanisms underlying the response to ionizing radiation can confer tumor cells with radioresistance, leading to radiotherapy failure. Therefore, the mechanisms underlying tumor cell radioresistance must be elucidated to provide novel approaches for tumor treatment.

After irradiation (IR), tumor cells produce reactive oxygen species (ROS), which then attack the bases and phosphoribosyl skeleton of DNA, causing different degrees of damage [3], including base damage, DNA single-strand breaks, DNA double-strand breaks (DSBs) [4], and DNA–DNA and DNA–protein cross-linking [5]. Among these types of DNA damage, DSBs are the most cytotoxic to irradiated cells [6], which activate response mechanisms [7]. Specifically, irradiated cells respond by halting the cell cycle and attempting to eliminate or repair the damage. DNA repair allows the cell to survive, whereas unrepaired or misrepaired DSBs can cause genomic instability and trigger cell senescence or death [8]. Currently, although DNA damage repair is a proven cause of radioresistance, the exact mechanisms underlying this phenomenon remain elusive.

To explore the mechanisms behind radioresistance in tumors, a previous study performed RNA-seq on A549 human lung adenocarcinoma cells after radiotherapy and found that Cyclin-Dependent Kinase Inhibitor 1A (CDKN1A) is highly expressed. The CDKN1A-encoded p21 protein regulates cell cycle, proliferation, differentiation, and senescence [[9], [10], [11], [12]]. Three distinct patterns of p21 dynamics determine different cell fates after drug treatment [13]. p21 is also related to tumor chemoresistance [14,15] and DNA repair regulation after radiotherapy [16,17]. These studies suggest that CDKN1A plays a role in tumor radioresistance by regulating DNA damage repair. However, its exact mechanisms remain unclear.

In the present study, we aimed to clarify the mechanisms underlying CDKN1A-enhanced tumor radioresistance. The results of this study may serve as a reference for developing novel targeted treatments against cancer.

## 2 Materials and methods

### 2.1 Cells and cell culture

We purchased A549 and H1650 cells from the Cell Bank of the Chinese Academy of Sciences (Shanghai, China). The cells were cultured in RPMI-1640 medium which contained 10% fetal bovine serum and 5 mg/ml penicillin/streptomycin. The cultivation conditions were 37 °C and 5% CO_2_. The culture media and materials involved in this experiment were purchased from Gibco (Grand Island, NY, USA).

### 2.2 Animals

Female BALB/C nude mice (GemPharmatech, Chengdu, China) weighing 18–22 g and aged 6–8 weeks were fed and watered sterilely. All animal experiments were approved by Chengdu Medical College's Experimental Animal Ethics Committee (Approval No. CMC-IACUC-2022050) and carried out in accordance with the Ministry of Health of the People's Republic of China's experimental animal guidelines (January 25, 1998).

### 2.3 Antibodies

Antibodies against CDKN1A (10355-1-AP), interleukin (IL)-18 (10663-1-AP), cleaved poly (ADP-ribose) polymerase (C-PARP, 13371-1-AP), glutathione peroxidase 4 (GPX4, 67763-Ig), HMGB1 (10829-1-AP), lipidation of microtubule-associated protein 1 light chain 3 form II (LC3-II, 14600-1-AP), P53BP1 (20002-1-AP), and GAPDH (60004-1-Ig) were obtained from Proteintech (Wuhan, China). Antibodies against caspase-3 (CASP-3, D3R6Y), cleaved-IL-1β (83186), γH2AX (9718), nucleotide-binding and oligomerization domain-like receptor (NLR) pyrin domain-containing 3 (NLRP3, 15101), and cleaved-caspase-1 (89332) were obtained from Cell Signaling Technology (Danvers, MA, USA). Antibodies against absent in melanoma 2 (AIM2, ab93015), gasdermin-D (GSDMD)-N (ab215203), and apoptosis-associated speck-like protein containing a C-terminal caspase recruitment domain (ASC, ab180799) were purchased from Abcam (Massachusetts, UK). Horseradish peroxidase (HRP)-conjugated AffiniPure goat anti-mouse IgG (SA00001-1) and HRP-conjugated AffiniPure goat anti-rabbit IgG (SA00001-2) were purchased from Proteintech. Cy3-labeled goat anti-mouse IgG (H + L) (A0521) and Cy3-labeled goat anti-rabbit IgG (H + L) (A0516) were purchased from Beyotime (Shanghai, China).

### 2.4 RNA sequencing analysis

RNA sequences were analyzed by CloudSeq Pte Ltd. (Shanghai, China). The data set supporting the conclusions in this paper was obtained from the GEO database (GSE124396) and hyperlinked to the dataset(s) in https://www.ncbi.nlm.nih.gov/geo/query/acc.cgi?acc=GSE124396.

### 2.5 CDKN1A silencing and overexpression

To knock down CDKN1A, We used Lipofectamine 3000 (Invitrogen, Carlsbad, CA, USA) to transfect A549 and H1650 cells with CDKN1A small interfering RNA (siRNA) which contains three sequences (Si#01: GATGGAACTTCGACTTTGT, Si#02: GAGACTCTCAGGGTCGAAA, Si#03: AGACCATGTGGACCTGTCA, RiboBio, Guangzhou, China). RiboBio provided the appropriate negative control. To stably knock down CDKN1A in cells, we created a siRNA targeting Si#02 and placed it into a pHBLV-U6-MCS-CMV-ZsGreen-PGK-PURO lentiviral vector (HanBio, Shanghai, China).

To increase CDKN1A expression in A549 and H1650 cells, we cloned the CDKN1A sequence into a pHBLV-CMV-MCS-3FLAG-EF1-ZsGreen-T2A-PURO lentiviral vector. An empty vector was used as a negative control. The knockdown and overexpression efficiencies were assessed using real-time fluorescence quantitative PCR (RT-qPCR) and western blotting (WB).

### 2.6 Real-time fluorescent quantitative PCR

Using a total RNA extraction kit (Solarbio, Beijing, China) and according to the manufacturer's instructions, total RNA was isolated from the cells. An iScript cDNA synthesis kit (Bio-Rad, Hercules, CA, USA) was used to reverse-transcribe 1 μg of total RNA to synthesize cDNA. Using the Bio-Rad CFX96 real-time equipment and SYBR Green Supermix, qRT-PCR was carried out. The forward primer of CDKN1A is GCTGAGCCGCGACTGTGATG, and the reverse is CCTCCAGTGGTGTCTCGGTGAC. GAPDH (Sangon Biotech, Shanghai, China) was used as the internal control. The forward primer of GAPDH is CAGGAGGCATTGCTGATGAT, and the reverse is GAAGGCTGGGGCTCATTT. The 2^–ΔΔCT^ method was used to calculate the relative expression levels.

### 2.7 Western blot analysis

Cells and tumor tissues were treated with RIPA lysis buffer (P0013B, Beyotime) on ice for 30 min, followed by centrifugation at 12,000×*g* for 15 min. The resulting supernatants were collected and analyzed using an enhanced bicinchoninic acid protein assay kit (P0010S, Beyotime) to determine protein concentration. Next, the proteins were separated by performing sodium dodecyl sulfate-polyacrylamide gel electrophoresis on 12% or 15% gels, and then transferred onto polyvinylidene difluoride membranes. The membranes were then blocked with a solution of Tris-buffered saline containing 0.1% Tween20 (TBST) and 5% fat-free milk, followed by overnight incubation with primary antibodies at 4 °C. After washing thrice with TBST, the membranes were incubated with a secondary *anti*-IgG antibody at 37 °C for 1–2 h and washed again thrice with TBST. Visualization of protein bands was achieved by using an enhanced chemiluminescent HRP substrate (WBKLS0500, Millipore, Burlington, MA, USA) and detected using the Universal Hood II (Bio-Rad) system and Image Lab Software (Bio-Rad). Finally, the bands were quantified using Quantity One 5.2 software (Bio-Rad, USA).

### 2.8 Cell viability assay

Cells were seeded at a density of 10,000 cells per well in 96-well plates, 24 h after exposure to radiation and/or drug treatment. Cell viability was assessed using Cell Counting Kit (CCK)-8 (C0038, Beyotime, Shanghai, China) following the manufacturer's instructions.

### 2.9 Colony formation assays

After being categorized based on experimental conditions, cells were seeded at a density of 1000 cells per well in 6-well plates and cultured for 24 h. Subsequently, they were exposed to irradiation using an X-RAD 160-225 instrument (42 cm; 225 kV/s; 12.4 mA; 2.0 Gy/min; filter, 2 mm aluminum; Precision X-Ray, North Branford, CT, USA). Following a 14-day culture period, the colonies were fixed with methanol and stained with crystal violet solution, and then the colony numbers were quantified.

### 2.10 Flow cytometry

The cells that underwent treatment were gathered and rinsed with PBS two times. Subsequently, they were stained using an Annexin V-PE/7-AAD apoptosis detection kit (KGA1015-1018, Key GEN BioTECH, Nanjing, China) following the instructions provided by the manufacturer. The stained cells were then examined using a DxFLEX flow cytometer (Beckman), and the resulting data were analyzed using the CytExpert software for DxFLEX.

### 2.11 Caspase-1 activity assay

Cells were seeded at a density of 10,000 cells per well in 96-well plates, 24 h after exposure to radiation ，a caspase-1 activity assay kit (C1102, Beyotime) was used to detect caspase-1 activity following the manufacturer's instructions.

### 2.12 Enzyme-linked immunosorbent assay

The commercial enzyme-linked immunosorbent assay (ELISA) kits were used to detect the concentrations of IL-18 and IL-1β in serum and supernatant following the manufacturer's instructions.

### 2.13 Lactate dehydrogenase release assay

Cells were seeded at a density of 10,000 cells per well in 96-well plates, 24 h after exposure to radiation ，lactate dehydrogenase (LDH) cytotoxicity assay kit (C0016, Beyotime) was used to detect the LDH release following the manufacturer's instructions.

### 2.14 Immunofluorescence

Cells were seeded at a density of 20,000 cells per well in 24-well plates, 24 h after exposure to radiation ，the grown cells were fixed in methanol that had been chilled to −20 °C for 15 min, rinsed three times in PBS, and blocked for 1 h in PBS that included 0.25% Triton X-100 and 10% normal goat serum. The cells were incubated for 24 h at 4 °C using the primary antibody solution, followed by three PBS washes, 1 h at 37 °C using a Cy3-conjugated secondary antibody stain, 10 min of DAPI staining, and three PBS washes. A DM4000 microscope (LEICA, Wetzlar, Germany) with a DFC450C camera and a 20 × or 40 × objective lens (LEICA) was used to take all of the immunofluorescence pictures. The LAS V4.5 controller software (LEICA) was used to process the images.

### 2.15 Co-immunoprecipitation

Cells were seeded at a density of 100,000 cells per well in 6-well plates, 24 h after exposure to radiation, the RIPA buffer was used to lyse cells, Protein A + G agarose (P2055-10 ml, Beyotime) was used for immunoprecipitation following the manufacturer's instructions.

### 2.16 Comet assay

Cells were seeded at a density of 100,000 cells per well in 6-well plates, 24 h after exposure to radiation, the trypsin was used to split the cells into individual cells. A DNA damage detection kit (KGA240, Key GEN BioTECH, Nanjing, China) was used to perform a comet assay following the manufacturer's instructions. With the aid of CASPlab software, the cell trailing rate and mean tail length were determined and examined.

### 2.17 Mouse xenograft model

Four treatment groups, consisting of four mice each, were randomly assigned to the animals: Vector, CDKN1A, IR + Vector, and IR + CDKN1A. A549 cells (1 × 10^6^) with or without CDKN1A overexpression were suspended in 100 μl PBS and subcutaneously injected into each mouse's axilla. The mice in the IR groups were given a weekly dose of 4 Gy of radiation using an X-RAD 160-225 device under anesthesia, 7 days after the implantation of their cells. To make sure that the radiation exclusively reached the subcutaneous tumor in the axilla, individual cylindrical lead cover shields were utilized. Every seven days, the tumor development was measured with Vernier calipers. Formula V = (a × b^2^)/2 was used to compute the tumor volume, where a and b represent the maximum and minimum diameters, respectively, in millimeters. The mice were euthanized and weighed immediately following dissection after four weeks.

### 2.18 Histopathologic analysis

The tumor tissues were embedded in paraffin at 58–60 °C after being treated with 4% formaldehyde. A microtome (HistoCore BIOCUT, LEICA) was then used to segment the tissues into 5 μm slices. A hematoxylin–eosin staining kit (G1120; Solarbio, Beijing, China) was used to stain the tumor sections following the manufacturer's instructions. A SPlink detection kit (SP-9000, ZSGB-BIO, Beijing, China) was used for immunohistochemical staining following the manufacturer's instructions. A DAB reagent kit (ZLI-9019, ZSGB-BIO, Beijing, China) was used to assess the immunohistochemistry following the manufacturer's instructions. A DM4000 microscope with a DFC450C camera and a 20 × or 40 × objective lens was used to take all of the immunofluorescence pictures. The LAS V4.5 controller software was used to process the images.

### 2.19 Statistical analysis

An unpaired Student's *t*-test was used to assess statistical significance when comparing two groups. One-way or two-way analysis of variance with Tukey's multiple comparison post-hoc test was used to statistically assess experiments involving more than two groups. GraphPad Prism 7 (San Diego, CA, USA) was used for all statistical analyses. The mean ± SD is used to present the data. At p < 0.05, statistical significance was established. Each experiment was performed independently at least thrice.

## 3 Results

### 3.1 CDKN1A promoted lung adenocarcinoma cell survival after radiotherapy

**Fig. 1:**
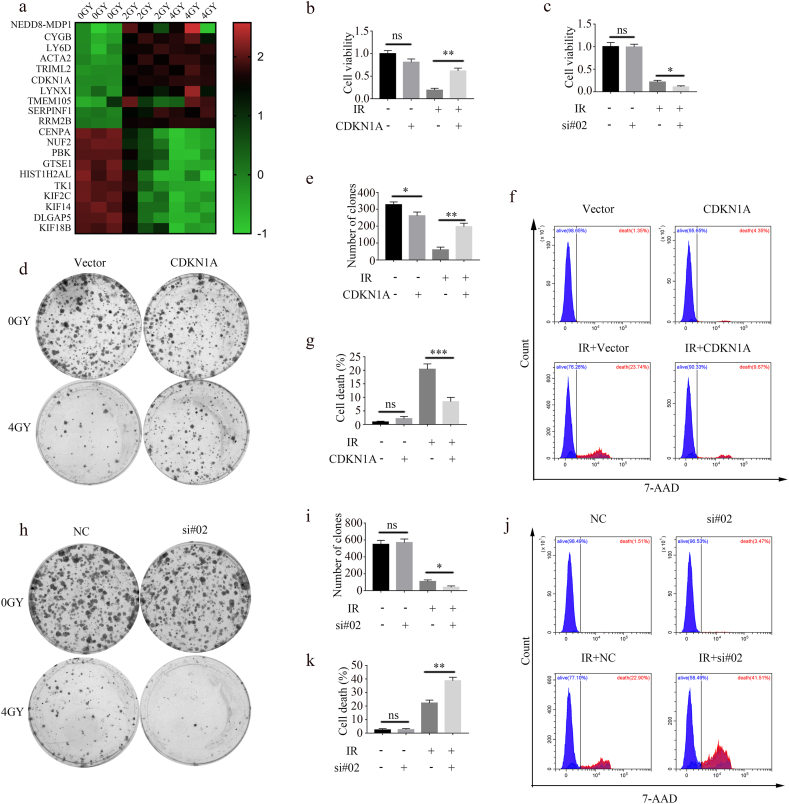
CDKN1A promotes A549 cell survival after radiotherapy. (a) Heat map of gene expression before and after radiotherapy. (b) A549 cells were exposed to 4 Gy radiation after CDKN1A overexpression, and cell viability was assessed using CCK-8 assay (mean ± SD, one-way ANOVA with Tukey's multiple comparison test; third vs. fourth group p < 0.01, n = 3 independent experiments). (c) A549 cells were exposed to 4 Gy radiation after CDKN1A knockdown, and cell viability was assessed using CCK-8 assay (mean ± SD, one-way ANOVA with Tukey's multiple comparison test; third vs. fourth group p < 0.05, n = 3 independent experiments). (d, e) Radiation-induced death of A549 cells with CDKN1A overexpression was monitored using colony formation assays. Representative images (d) and quantitation (e) are shown (mean ± SD, one-way ANOVA with Tukey's multiple comparison test; third vs. fourth group p < 0.01, n = 3 independent experiments). (f, g) Radiation-induced death of CDKN1A-overexpressing A549 cells was detected via flow cytometry. Representative images (f) and quantitation (g) are shown (mean ± SD, one-way ANOVA with Tukey's multiple comparison test; third vs. fourth group p < 0.001, n = 3 independent experiments). (h, i) Radiation-induced death of CDKN1A-knockdown A549 cells was monitored using colony formation assays. Representative images (h) and quantitation (i) are shown (mean ± SD, one-way ANOVA with Tukey's multiple comparison test; third vs. fourth group p < 0.05, n = 3 independent experiments). (j, k) Radiation-induced death of CDKN1A-knockdown A549 cells was detected using flow cytometry. Representative images (j) and quantitation (k) are shown (mean ± SD, one-way ANOVA with Tukey's multiple comparison test; third vs. fourth group p < 0.01, n = 3 independent experiments).

To explore the mechanisms underlying tumor radioresistance, we performed RNA-seq in A549 cells after radiotherapy. Fig. 1a shows the top 10 upregulated and downregulated genes.

Considering that the coefficient of gene expression variation was less than 15%, we selected CDKN1A as our target gene and confirmed that CDKN1A was upregulated in the irradiated A549 cells (Figs. S1a–c). To investigate the effect of CDKN1A on A549 cells after IR, we overexpressed (Figs. S1d–f) and knocked down CDKN1A (Figs. S1g–i) in A549 cells. After IR, cell viability was evaluated using a CCK-8 assay. The results showed that the viability of the irradiated A549 cells significantly decreased, and CDKN1A overexpression reversed the radiation damage (Fig. 1b and c). CDKN1A overexpression increased radioresistance (Fig. 1d and e) and survival of A549 cells following IR (Fig. 1f and g), whereas CDKN1A knockdown increased A549 cell radiosensitivity (Fig. 1h–k). We also treated H1650 cells using the same method and obtained similar results (Fig. S2). These findings showed that CDKN1A increased the radioresistance and survival of lung cancer cells following radiation.

### 3.2 CDKN1A upregulation inhibited radiation-induced pyroptosis in A549 cells

**Fig. 2:**
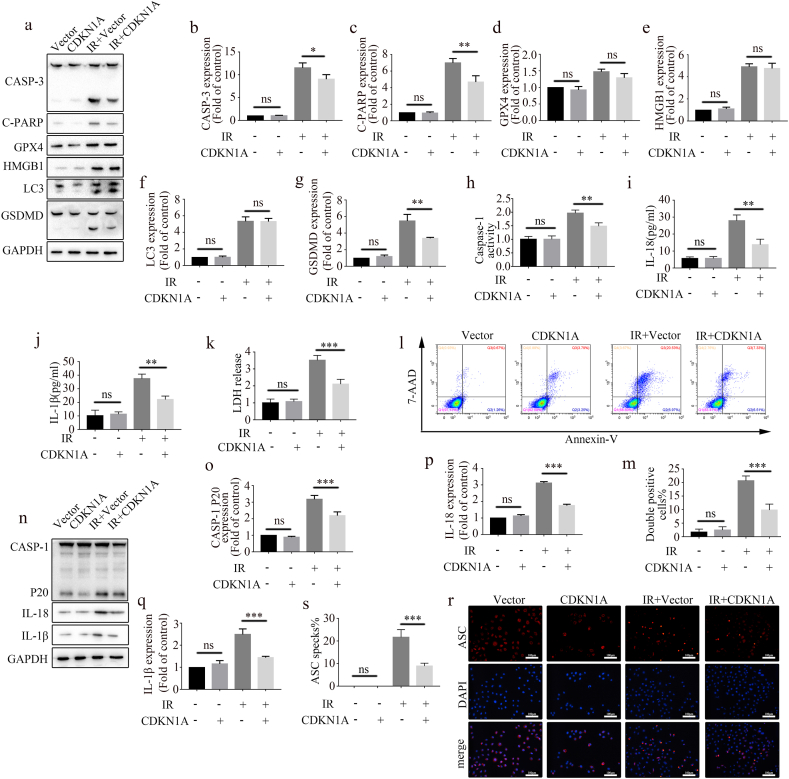
CDKN1A upregulation inhibits radiation-induced pyroptosis in A549 cells. (a–g) Western blot analysis of indicated proteins (a) and quantification graphs (b–g) in CDKN1A-overexpressing and control cells following radiation treatment. (h) Caspase-1 activity in A549 under different treatment conditions (mean ± SD, one-way ANOVA with Tukey's multiple comparison test; third vs. fourth group p < 0.01, n = 3 independent experiments). (i) IL-18 concentration in the supernatant of A549 cultures under different treatment conditions (mean ± SD, one-way ANOVA with Tukey's multiple comparison test; third vs. fourth group p < 0.01, n = 3 independent experiments). (j) IL-1β concentration in the supernatant of A549 cultures under different treatment conditions (mean ± SD, one-way ANOVA with Tukey's multiple comparison test; third vs. fourth group p < 0.01, n = 3 independent experiments). (k) Cell death assessment based on the amount of LDH released into the supernatant (mean ± SD, one-way ANOVA with Tukey's multiple comparison test; third vs. fourth group p < 0.001, n = 3 independent experiments). (l, m) Irradiation-induced death in A549 cells by 7AAD and Annexin V cytometry assay (l) and quantification graphs (m; mean ± SD, one-way ANOVA with Tukey's multiple comparison test; third vs. fourth group p < 0.001, n = 3 independent experiments). (n–q) Representative blots of caspase-1, p20, IL-18, and IL-1β expression in A549 cells were determined using Western blot (n) and quantification graphs (o–q). (r, s) ASC specks detected by immunofluorescence staining (r) and quantification graphs (s; mean ± SD, one-way ANOVA with Tukey's multiple comparison test; third vs. fourth group p < 0.001, n = 3 independent experiments). Scale bar: 100 μm.

Radiation can cause cytotoxicity, leading to different types of cell death [18,19]. To understand which type of cell death was triggered by CDKN1A after radiotherapy, we examined biomarkers of several commonly regulated cell death pathways. WB assay results showed that the expression of apoptosis biomarkers (cleaved CASP-3 and C-PARP) and pyroptosis (cleaved GSDMD) was downregulated in CDKN1A-overexpressing cells after IR, whereas the expression of ferroptosis biomarkers (GPX4), necroptosis/necrosis (HMGB1), and autophagy (LC3-II) did not change significantly (Fig. 2a–g). Although the role of CDKN1A in apoptosis has been supported by many reports, its function in pyroptosis is poorly understood. Therefore, we focused on pyroptosis in the present study.

The caspase-1 activity in the irradiated A549 cells increased, whereas CDKN1A overexpression reversed caspase-1 activity in the irradiated A549 cells (Fig. 2h). ELISA results showed that the IL-18 and IL-1β levels in the supernatant decreased in the CDKN1A overexpression group after IR (Fig. 2i and j). During cell pyroptosis, the permeability of the cell membrane changes, and LDH is released. In the present study, LDH release assay results showed that CDKN1A overexpression significantly inhibited LDH release in the irradiated A549 cells (Fig. 2k). Then, pyroptosis was assessed by flow cytometry. The rate of pyroptosis increased considerably following IR, whereas CDKN1A overexpression decreased this impact (Fig. 2l and m). WB results showed that IR dramatically elevated caspase-1 p20, IL-18, and IL-1β levels in the cells, but CDKN1A overexpression lowered them (Fig. 2n–q). Immunofluorescence revealed that ASC oligomerized, with the number of ASC specks considerably increasing after IR but decreasing after CDKN1A overexpression (Fig. 2r and s). Taken together, these findings indicate that CDKN1A overexpression prevents pyroptosis in A549 cells after radiation.

### 3.3 CDKN1A knockdown after overexpression reversed the suppression of radiation-induced pyroptosis by CDKN1A in A549 cells

To further verify the role of CDKN1A in inhibiting radiation-induced pyroptosis, we knocked down CDKN1A in A549 cells after CDKN1A overexpression. IR increased caspase-1 activity in the A549 cells, and CDKN1A knockdown further enhanced this effect (Fig. S3a). LDH release assay results showed that CDKN1A knockdown significantly promoted LDH release after IR (Fig. S3b). The CDKN1A knockdown group's supernatant IL-18 and IL-1β levels increased during IR, according to ELISA data (Figs. S3c and d). The pyroptosis rate of the irradiated A549 cells increased significantly following CDKN1A knockdown, according to flow cytometric data (Figs. S3e and f). Furthermore, WB findings showed that following CDKN1A knockdown, the levels of caspase-1 p20, IL-18, and IL-1β in the irradiation A549 cells increased considerably (Figs. S3g–i). Following CDKN1A knockdown, immunofluorescence demonstrated a considerable increase in the amount of ASC specks in the irradiated A549 cells (Figs. S3k and l).

### 3.4 CDKN1A upregulation inhibited the activation of the AIM2 and NLRP3 inflammasomes in A549 cells after IR

**Fig. 3:**
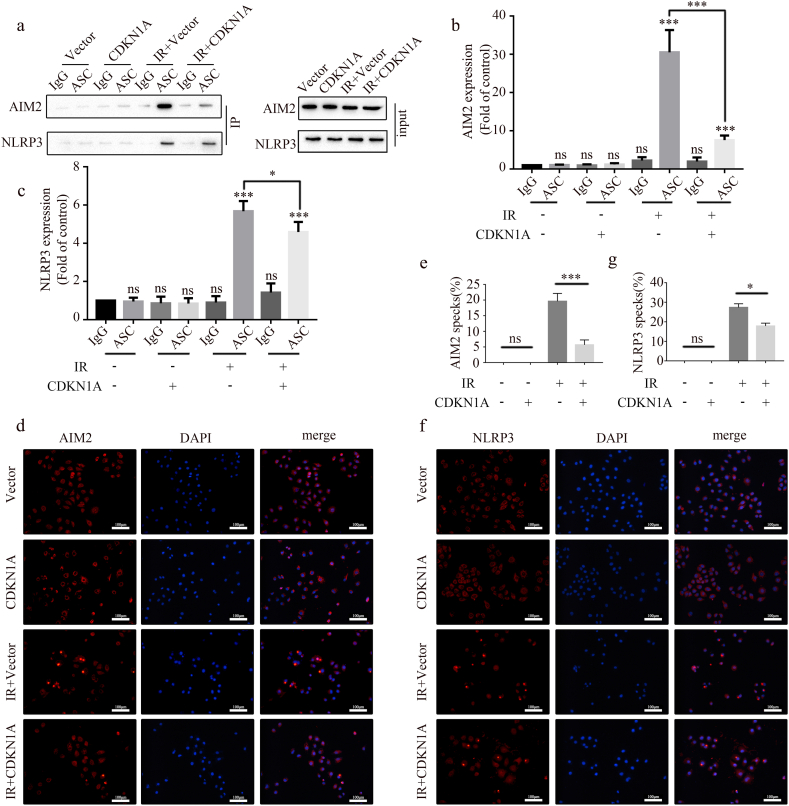
CDKN1A upregulation inhibits the activation of the AIM2 and NLRP3 inflammasomes in A549 cells after radiation. (a–c) Activation of AIM2 and NLRP3 inflammasomes detected using CO-IP (a) and quantification graphs (b,c). (d, e) AIM2 specks detected through immunofluorescence staining (d) and quantification graphs (e) (mean ± SD, one-way ANOVA with Tukey's multiple comparison test; third vs. fourth group p < 0.001, n = 3 independent experiments; scale bar: 100 μm). (f, g) NLRP3 specks detected using immunofluorescence staining (f) and quantification graphs (g; mean ± SD, one-way ANOVA with Tukey's multiple comparison test; third vs. fourth group p < 0.05, n = 3 independent experiments).

Radiation can cause DNA damage and release double-strand DNA into the cytosol and extracellular space, which can activate the AIM2 inflammasome [[20], [21], [22]]. Simultaneously, radiation can increase ROS production and induce Ca^2+^and K + influx, activating the NLRP3 inflammasome [[23], [24], [25]]. In the present study, we investigated whether CDKN1A affects the radiation-mediated activation of the AIM2 and NLRP3 inflammasomes through a co-immunoprecipitation assay. As shown in Fig. 3a–c, CDKN1A overexpression significantly reduced the assembly of the AIM2 inflammasome after IR but only slightly decreased that of the NLRP3 inflammasome. Immunofluorescence showed that CDKN1A overexpression reduced the number of AIM2 and NLRP3 specks in the irradiated A549 cells (Fig. 3d–g), but its effect was stronger on the AIM2 inflammasome than on the NLRP3 inflammasome (Fig. 3e–g). When considered collectively, these findings imply that in irradiated A549 cells, CDKN1A overexpression prevents the activation of the AIM2 and NLRP3 inflammasomes.

**Fig. 4:**
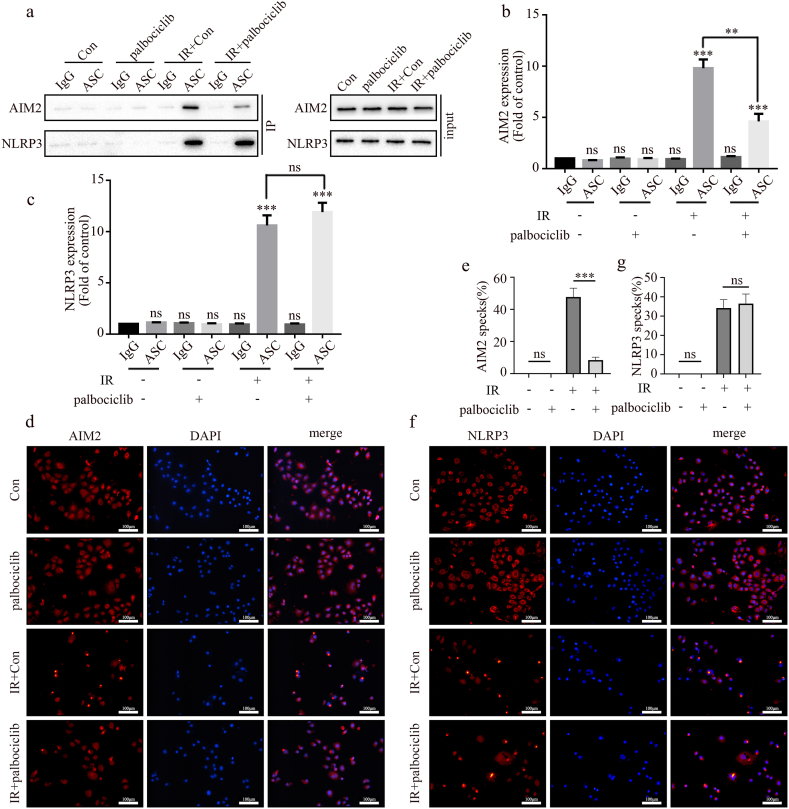
Palbociclib inhibits the activation of the AIM2 inflammasomes but not NLRP3 inflammasomes in A549 cells after radiation. (a–c) Activation of AIM2 and NLRP3 inflammasomes detected using CO-IP (a) and quantification graphs (b, c) after palbociclib treatment. (d, e) After palbociclib treatment, AIM2 specks were detected through immunofluorescence staining (d), and quantification graphs are shown (e; mean ± SD, one-way ANOVA with Tukey's multiple comparison test; third vs. fourth group p < 0.001, n = 3 independent experiments; scale bar: 100 μm). (f, g) After palbociclib treatment, NLRP3 specks were detected using immunofluorescence staining (f), and quantification graphs are shown (g; mean ± SD, one-way ANOVA with Tukey's multiple comparison test; third vs. fourth group p = 0.8351, n = 3 independent experiments). Scale bar: 100 μm.

P21 can stop the cell cycle by preventing CDK2/4/6 from functioning. In order to investigate the potential impact of cell cycle arrest on the activation of AIM2 and NLRP3 inflammasomes, we inhibited cell cycle progression by using palbociclib, a specific inhibitor of CDK4/6. The activation of the AIM2 inflammasome was considerably decreased by palbociclib (2 μM, 24 h) treatment, but not that of the NLRP3 inflammasome, as seen in Fig. 4a–c. This result was further confirmed by using immunofluorescence (Fig. 4d–g). Thus, the cell cycle arrest caused by CDK4/6 inhibition only inhibits the activation of the AIM2 inflammasome but not that of the NLRP3 inflammasome.

### 3.5 CDKN1A affected DNA repair after IR

**Fig. 5:**
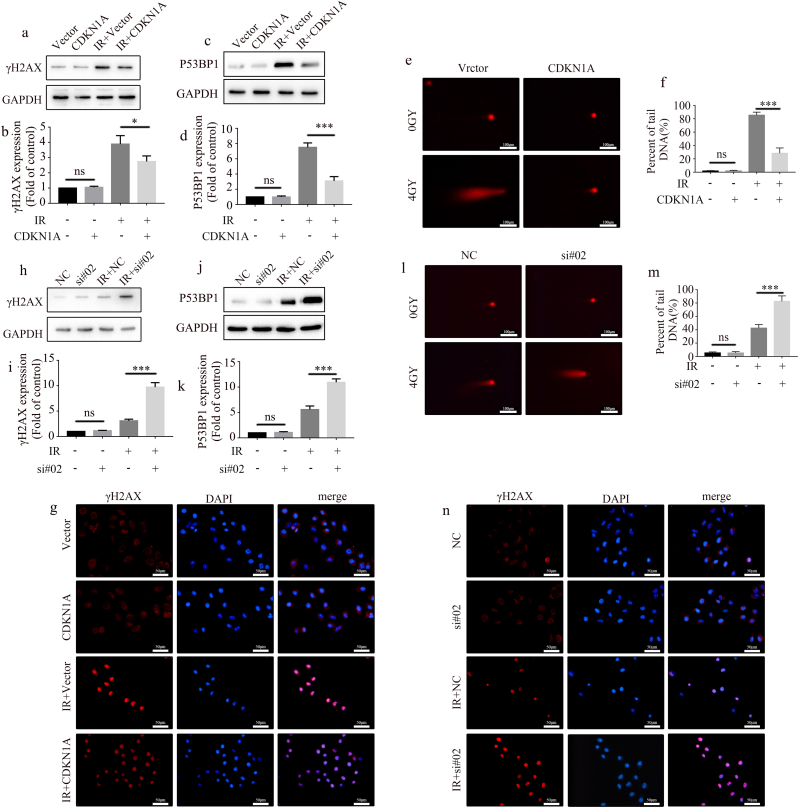
CDKN1A affects DNA repair after radiotherapy. (a, b) A549 cells were exposed to 4 Gy radiation after CDKN1A overexpression, and γH2AX expression was detected by Western blot (a) and the quantification graphs (b). (c, d) A549 cells were exposed to 4 Gy radiation after CDKN1A overexpression, and P53BP1 expression was detected by Western blot (c) and the quantification graphs (d). (e, f) A549 cells were exposed to 4 Gy radiation after CDKN1A overexpression, and DNA repair capacity was assessed using comet assay. Representative images (e) and quantitation (f) are shown (mean ± SD, one-way ANOVA with Tukey's multiple comparison test; the third vs fourth group p < 0.001, n = 3 independent experiments). Scale bar: 100 μm. (g) A549 cells were exposed to 4 Gy radiation after CDKN1A overexpression, and γH2AX was detected by immunofluorescence. Scale bar: 50 μm. (h, i) CDKN1A-overexpressing A549 cells were irradiated with 4 Gy after CDKN1A knockdown, and γH2AX was detected by WB (h) and the quantification graphs (i). (j, k) CDKN1A-overexpressing A549 cells were irradiated with 4 Gy after CDKN1A knockdown, and P53BP1 was detected by WB (j) and the quantification graphs (k). (l, m) CDKN1A-overexpressing A549 cells were irradiated with 4 Gy after CDKN1A knockdown, and DNA repair capacity was assessed by comet assay. Representative images (l) and quantitation (m) are shown (mean ± SD, one-way ANOVA with Tukey's multiple comparison test; the third vs fourth group p < 0.001, n = 3 independent experiments). Scale bar: 100 μm. (n) CDKN1A-overexpressing A549 cells were irradiated with 4 Gy after CDKN1A knockdown, and γH2AX was detected by immunofluorescence. Scale bar: 50 μm.

CDKN1A overexpression reduced the activation of AIM2 inflammasome in irradiated A549 cells, suggesting that CDKN1A can affect DNA repair after irradiation. Therefore, we analyzed γH2AX and P53BP1, two key markers of DNA damage. Our results showed that γH2AX and P53BP1expression were downregulated after CDKN1A overexpression in irradiated A549 cells (Fig. 5a–d). Comet assay results exhibited that CDKN1A overexpression significantly promoted the DNA repair capacity of the cells (Fig. 5e and f). Immunofluorescence demonstrated that γH2AX was localized in the nucleus, and its expression was downregulated in CDKN1A-overexpressing cells after irradiation (Fig. 5g). To further verify the role of CDKN1A in DNA repair after irradiation, we knocked down CDKN1A in A549 cells after CDKN1A overexpression. Results showed that CDKN1A knockdown upregulated γH2AX and P53BP1 expression after irradiation (Fig. 5h–k). Comet assay results demonstrated that CDKN1A knockdown significantly attenuated the cellular DNA repair capacity (Fig. 5l and m). Immunofluorescence analysis revealed that the expression of γH2AX was upregulated in CDKN1A knockdown cells after irradiation (Fig. 5n). Overall, these findings suggest that CDKN1A affects DNA repair in irradiated A549 cells.

### 3.6 CDKN1A upregulation enhanced A549 xenograft tumor radioresistance *in vivo*

**Fig. 6:**
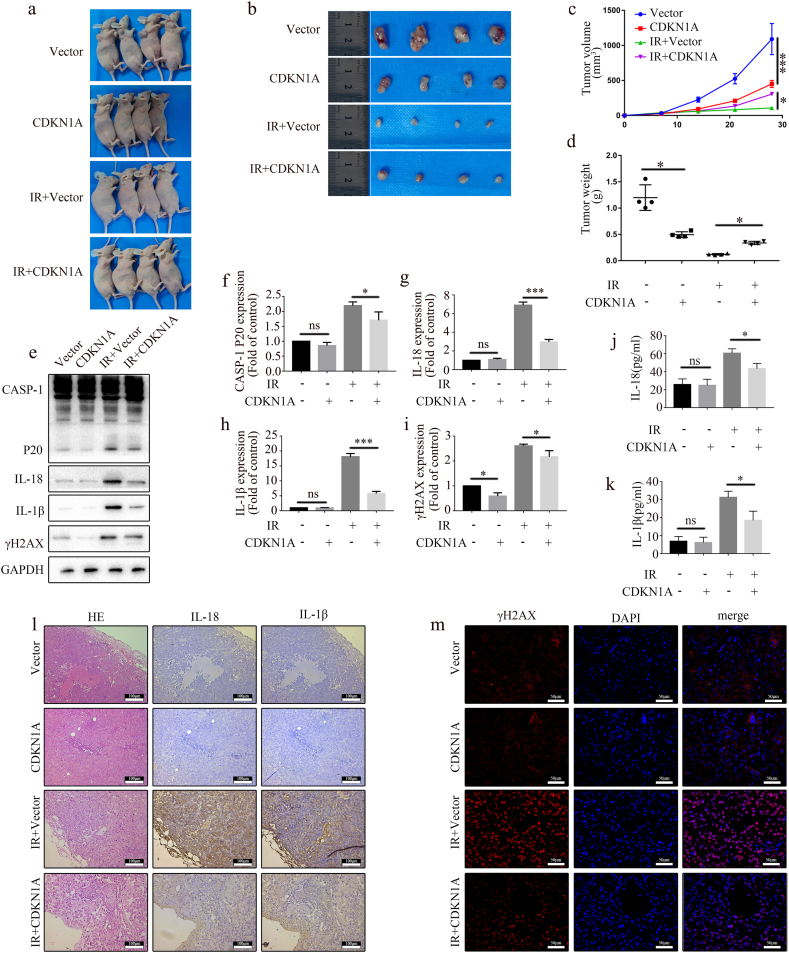
CDKN1A upregulation enhances A549 xenograft tumor radioresistance *in vivo*. (a) Images of nude mice with subcutaneous xenograft tumors. (b) Xenograft tumors isolated from sacrificed mice at the end of experiment. (c) Volumes of subcutaneous xenograft tumors (mean ± SD, two-way ANOVA with Tukey's multiple comparison test; third vs. fourth group p < 0.05, each group with four mice). (d) Average weights of xenograft tumors (mean ± SD, one-way ANOVA with Tukey's multiple comparison test; third vs. fourth group p < 0.05, each group with four mice). (e–i) Representative blots of caspase-1 p20, IL-18, IL-1β, and γH2AX expression in xenograft tumors determined by Western blot (e) and quantification graphs (f–i). (j) IL-18 concentration in serum (mean ± SD, one-way ANOVA with Tukey's multiple comparison test; third vs. fourth group p < 0.05, n = 3 independent experiments). (k) IL-1β concentration in serum (mean ± SD, one-way ANOVA with Tukey's multiple comparison test; third vs. fourth group p < 0.05, n = 3 independent experiments). (l) Representative images of xenograft tumors stained with hematoxylin–eosin, and IL-18 and IL-1β levels determined by immunohistochemistry. Scale bar: 100 μm. (m) γH2AX was detected by immunofluorescence in xenograft tumors. Scale bar: 50 μm.

In order to investigate the function of CDKN1A in radioresistance in more detail, we used A549 cells with or without CDKN1A overexpression to create a subcutaneous tumor model in nude mice. As shown in Fig. 6a and b, CDKN1A overexpression efficiently enhanced the radioresistance of A549 xenografts to concurrent radiation treatment. The A549 xenografts with CDKN1A overexpression had larger volumes and weights than those without CDKN1A overexpression after radiation (Fig. 6c and d). Altogether, these results indicate that CDKN1A upregulation enhances the radioresistance of A549 xenograft tumors *in vivo*.

We evaluated the indicators of A549 xenograft pyroptosis to determine whether CDKN1A can enhance the radioresistance of A549 xenografts *in vivo*. WB results revealed that caspase-1 p20, IL-18, and IL-1β levels significantly increased after IR and decreased following CDKN1A overexpression (Fig. 6e–h). The expression of γH2AX in the irradiated mice was downregulated after CDKN1A overexpression (Fig. 6e–i). Furthermore, ELISA data showed that the serum IL-18 and IL-1β levels in the CDKN1A overexpression group decreased after IR (Fig. 6j and k). Further examination of the A549 xenograft tissue showed that compared to the IR group alone, the A549 xenografts in the CDKN1A overexpression group exhibited reduced levels of IL-18 and IL-1β (Fig. 6l). Immunofluorescence showed that CDKN1A overexpression could reduce γH2AX expression in the irradiated mice (Fig. 6m). These results demonstrate that CDKN1A upregulation inhibits pyroptosis that caused by radiation *in vivo*.

## 4 Discussion

In the present study, CDKN1A enhanced radioresistance in lung adenocarcinoma cells. CDKN1A upregulation following radiotherapy promoted lung adenocarcinoma cell survival, as evidenced by the increased viability of irradiated CDKN1A-overexpressing lung adenocarcinoma cells. Furthermore, following CDKN1A overexpression, the irradiated mice's serum caspase-1 activity and IL-1β levels dropped. Cellularly, there was a decrease in the number of pyroptotic cells, along with a drop in caspase-1 activity, LDH release, IL-18 and IL-1β levels, and ASC speck count. Following CDKN1A overexpression, the NLRP3 and AIM2 inflammasomes' activation in the irradiated A549 cells diminished. These findings suggest that radiation-induced pyroptosis is inhibited by CDKN1A overexpression. Moreover, CDKN1A promoted post-radiation DNA repair because the expression of the DNA damage marker γH2AX in the irradiated A549 cells was reduced after CDKN1A overexpression. Taken together, these results demonstrate that CDKN1A inhibits pyroptosis to enhance the radioresistance of A549 cells by promoting DNA repair. Our results also showed that the expression of apoptosis biomarkers was downregulated in CDKN1A-overexpressing cells after IR, which indicates the possibility that CDKN1A may affect radiation resistance by regulating the crosstalk between apoptosis and pyroptosis. However, further investigation is still required to validate these findings.

The CDKN1A-encoded p21 protein is a potent CDK inhibitor. It regulates cell cycle progression during the G1 and S phases by directly or indirectly inhibiting the activities of CDK1, CDK2, CDK4, and CDK6, providing additional time for DNA repair [26]. Several studies have claimed that p21 can colocalize with DNA repair proteins at DNA damage sites, initiating the assembly of p21 to DNA excision foci [27]. In the present study, CDKN1A promoted DNA repair to enhance the radioresistance of A549 cells, which is consistent with the results of previous studies. However, other studies demonstrated that p21 downregulation is required for PCNA ubiquitination, which facilitates PCNA-dependent DNA repair [28]. Taken together, these results indicate that p21 plays a double-edged role in DNA repair. Furthermore, previous studies found that radiation-mediated DNA damage and subsequent repair involve HR and NHEJ, in which p21 may play an important role [[29], [30], [31]], However, the specific mechanism of action still requires further investigation.

Pyroptosis is a form of regulated cell death caused by inflammasome activation, mainly manifested by cell swelling, large bubbles in the plasma membrane, and the release of several pro-inflammatory cytokines [32]. The inflammasome complex during pyroptosis is mainly assembled by pattern recognition receptors, ASC, and pro-caspase-1 [33]. Examples of pattern recognition receptors include NLRP11, NLRP3, NLRP6, NLRP7, AIM2, and HNAIP-NLRC4; different pattern recognition receptor proteins can respond to distinct stimuli and activate the corresponding inflammasome assembly [34]. The inflammasome then cleaves and processes pro-caspase-1 into mature caspase-1. Activated caspase-1 cleaves GSDMD, releases the GSDMD-N-terminal domain with pore-forming activity, destroys the integrity of the plasma membrane, and releases the cell's contents, finally causing the inflammatory death of cells [35]. In the present study, CDKN1A inhibited the activation of the NLRP3 and AIM2 inflammasomes. We demonstrated that CDKN1A promoted DNA repair by inhibiting the activation of the AIM2 inflammasome; nonetheless, the mechanism by which CDKN1A inhibits the NLRP3 inflammasome remains unclear. The NLRP3 inflammasome could detect signs of metabolic stress [36], and CDKN1A could regulate cell metabolism [37], suggesting that CDKN1A inhibits the activation of the NLRP3 inflammasome by regulating cell metabolism. However, further studies are warranted to validate this hypothesis. Furthermore, our research demonstrated the ability of CDKN1A to promote DNA repair after radiation. However, the specific mechanism of action still requires further investigation.

## 5 Conclusions

Our findings indicate that CDKN1A inhibits the activation of AIM2 inflammasome by promoting DNA repair after radiotherapy, thus promoting the survival of lung adenocarcinoma cells. This discovery provides a novel idea for reducing the radioresistance of tumor cells. This study may serve as a reference for developing novel targeted therapies against cancer.

## Ethics statement

All animal experiments were approved by Chengdu Medical College's Experimental Animal Ethics Committee (Approval No. CMC-IACUC-2022050) and carried out in accordance with the Ministry of Health of the People's Republic of China's experimental animal guidelines (January 25, 1998).

## Data availability statement

The data set supporting the conclusions in this paper was obtained from the GEO database (GSE124396) and hyperlinked to dataset(s) in https://www.ncbi.nlm.nih.gov/geo/query/acc.cgi?acc=GSE124396.

The corresponding author can provide the datasets used and/or analyzed in the current work upon reasonable request.

## Funding

This study was funded by the 10.13039/501100001809National Natural Science Foundation of China (81972977, 82273574, 82273433), Foundation of Science and Technology Innovation Talent Project of Sichuan Province (2021038), Foundation of Health Commission of Sichuan Province (20ZD016), Foundation of Health Commission of Chengdu (2021001), Foundation of 10.13039/501100010822Chengdu Science and Technology Bureau (2021-YF05-00291-10.13039/100003351SN), Foundation of Sichuan Science and Technology Agency (2019YJ0589), Foundation of the First Affiliated Hospital of Chengdu Medical College (CYFY202LNZD01, CYFY2020YB05, CYFY2017ZD03, CYFY2018ZD02, and CYFY2019ZD06), and Disciplinary Construction Innovation Team Foundation of Chengdu Medical College (CMC-XK-2103).

## Authors' contributions

The data was gathered by JL and TL, who also authored, reviewed, and edited the publication. Research materials and procedures were provided by NT, SL, FZ, and WY. TZ and SD helped build the animal models. YX and DW oversaw the project and wrote, reviewed, and edited the manuscript. The final manuscript has been read and approved by all writers.

## Patient consent for publication

Not applicable.

## Declaration of competing interest

There are no disclosed conflicts of interest for the authors.
