# Supplementary material for: Cyclin-dependent kinase inhibitor 1A inhibits pyroptosis to enhance human lung adenocarcinoma cell radioresistance by promoting DNA repair
Source: Heliyon. 2024 Feb 29;10(5):e26975. doi: 10.1016/j.heliyon.2024.e26975 (PMC10926078; doi:10.1016/j.heliyon.2024.e26975)

Fig.2a – CASP3-Repeat1

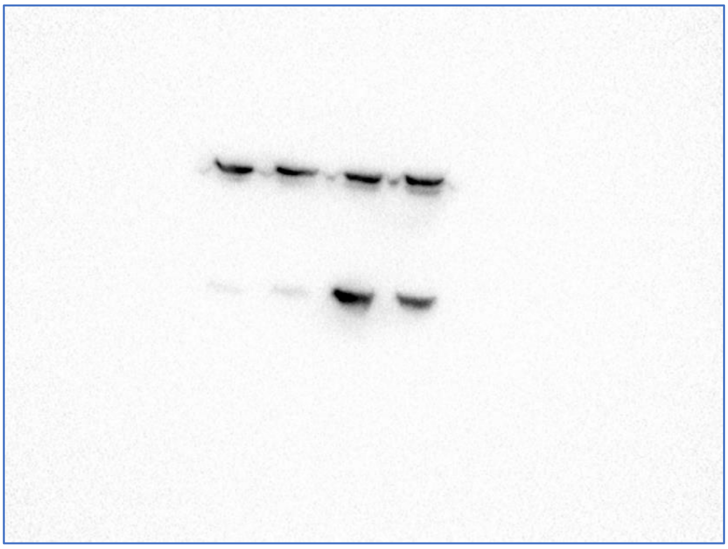

Fig.2a – CASP3-Repeat2

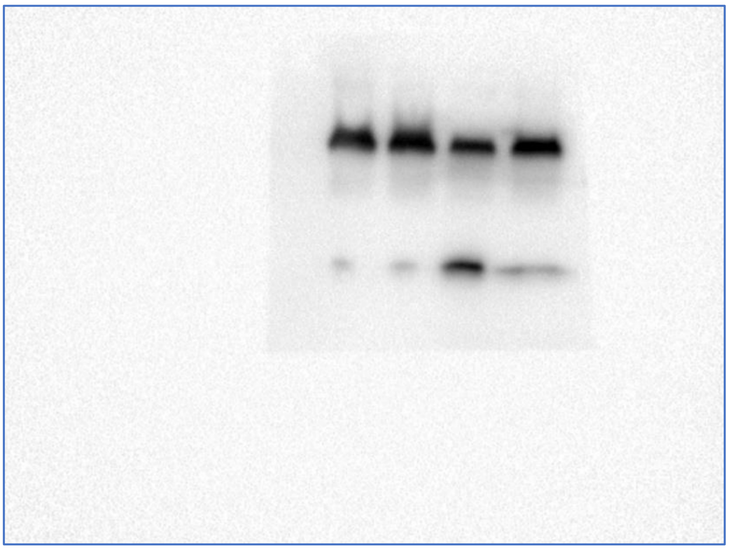

Fig.2a – CASP3-Repeat3

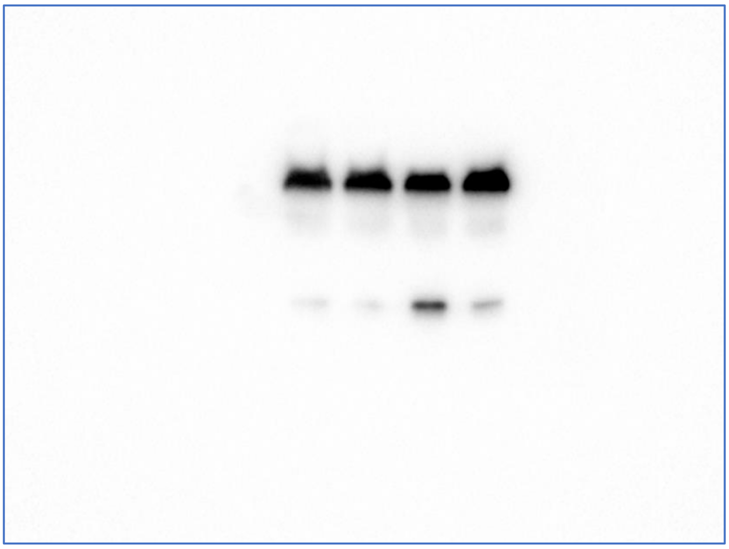

Fig.2a – C-PARP- Repeat1

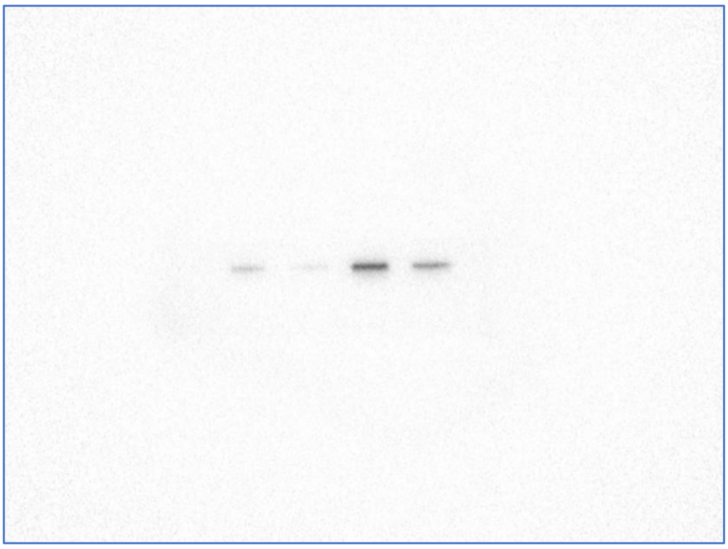

Fig.2a – C-PARP- Repeat2

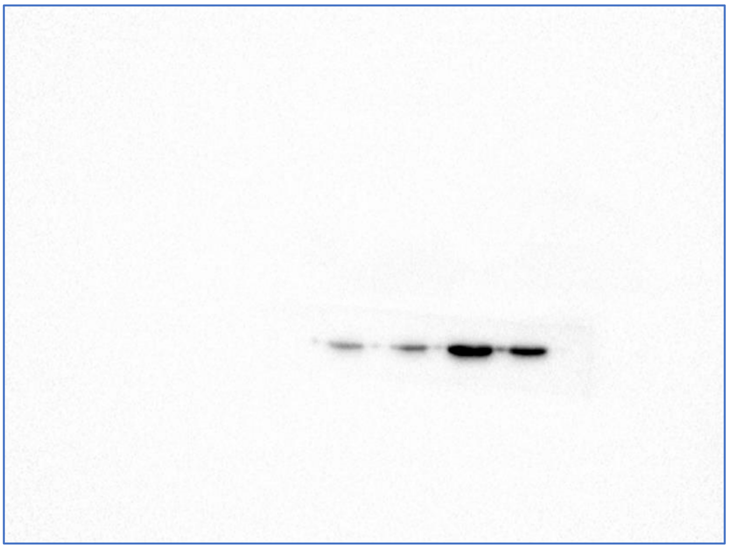

Fig.2a – C-PARP- Repeat3

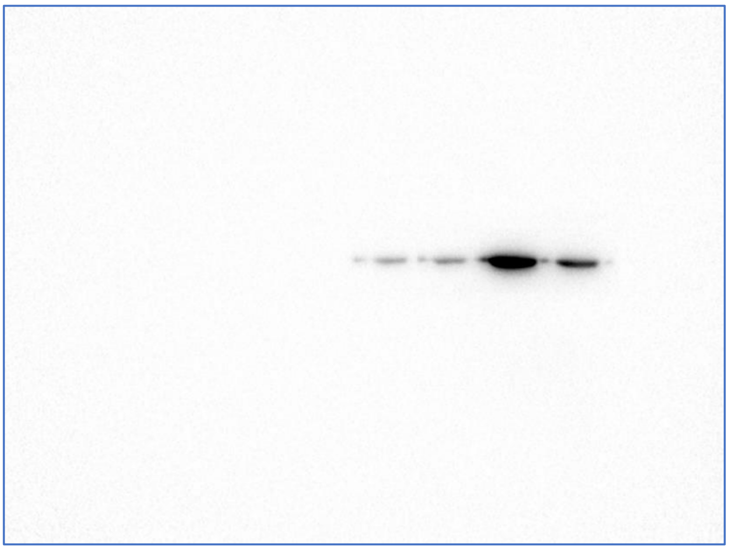

Fig.2a – GPX4- Repeat1

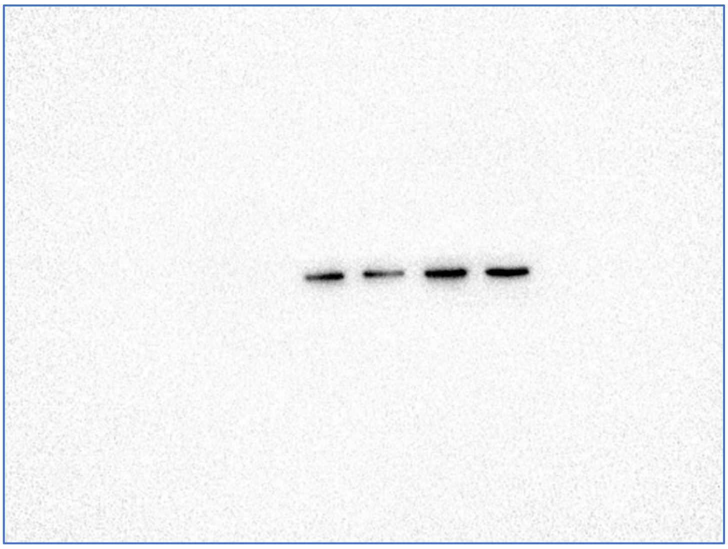

Fig.2a – GPX4- Repeat2

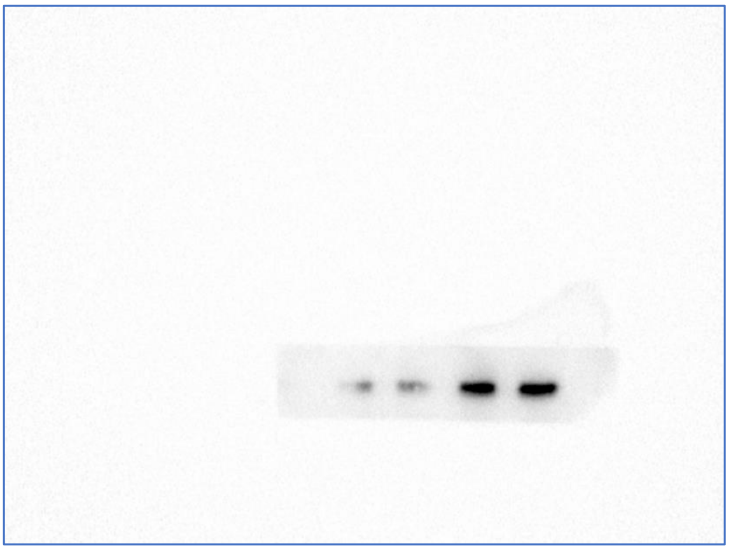

Fig.2a – GPX4- Repeat3

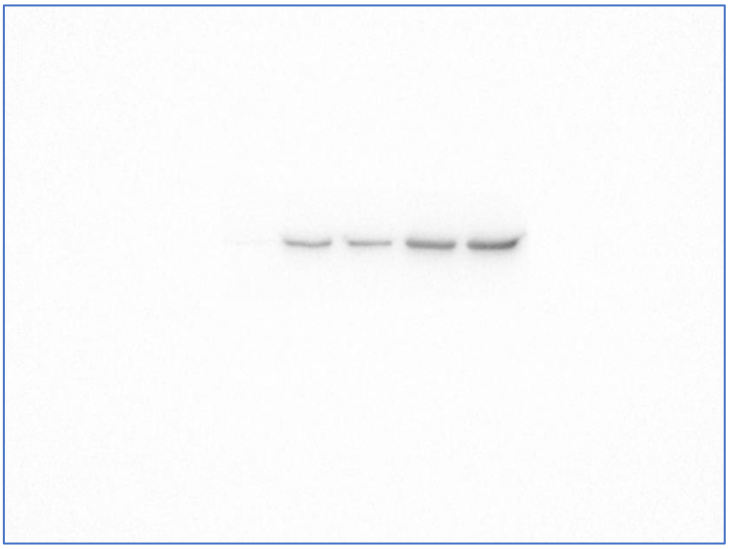

Fig.2a – HMGB1 - Repeat1

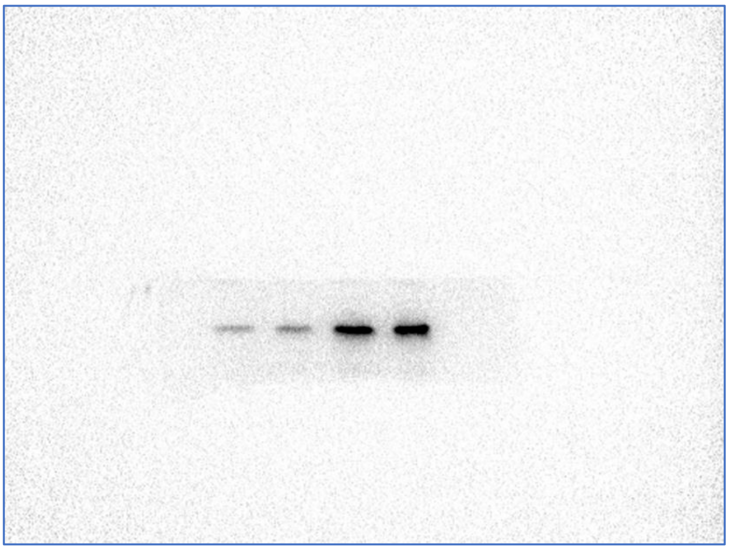

Fig.2a – HMGB1 – Repeat2

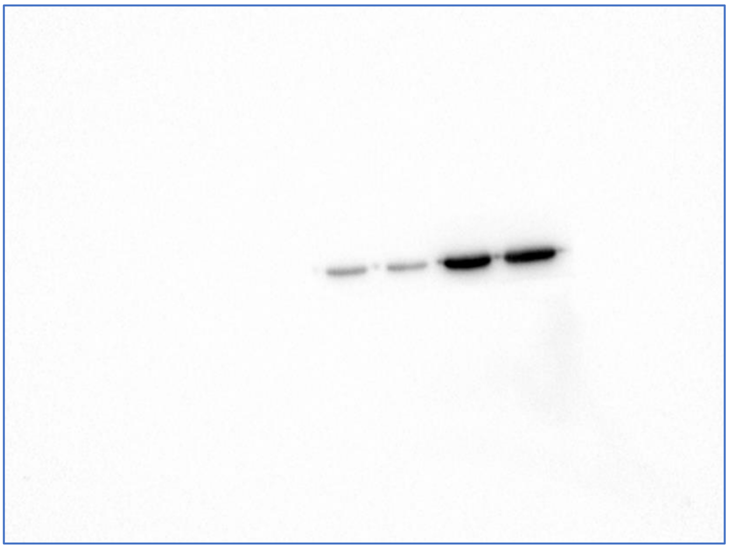

Fig.2a – HMGB1 – Repeat3

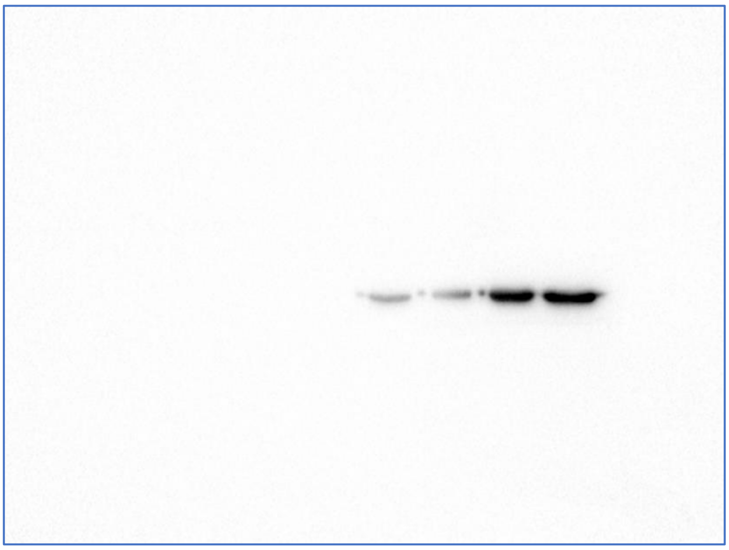

Fig.2a – LC3- Repeat1

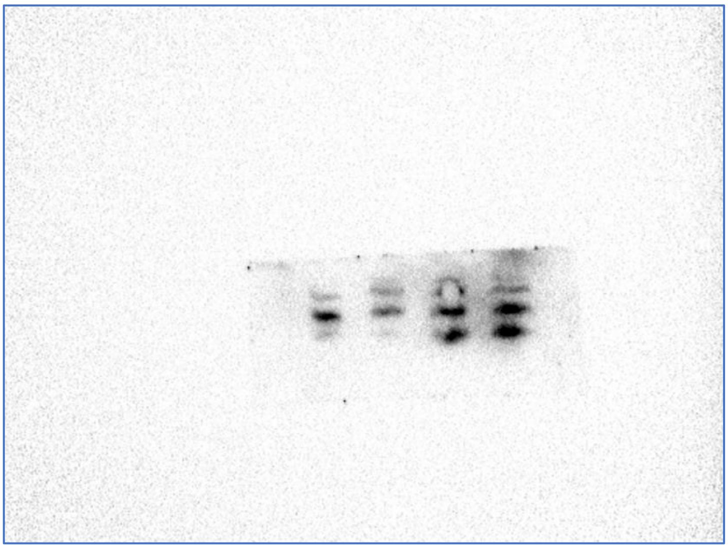

Fig.2a – LC3- Repeat2

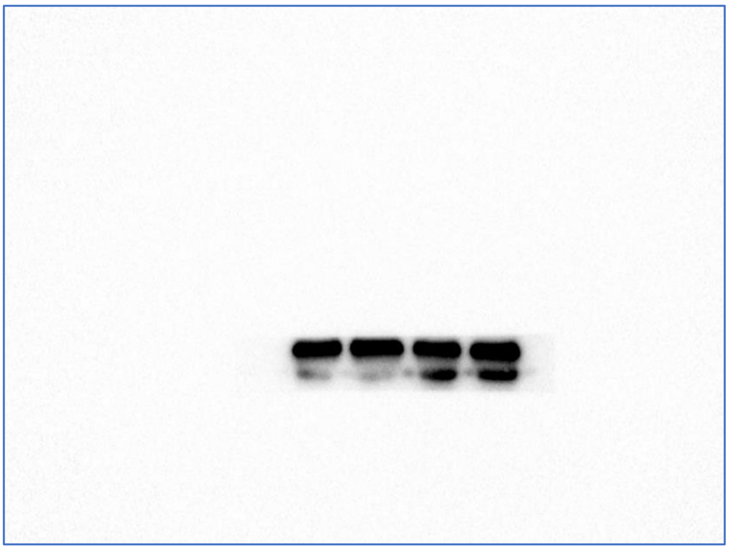

Fig.2a – LC3- Repeat3

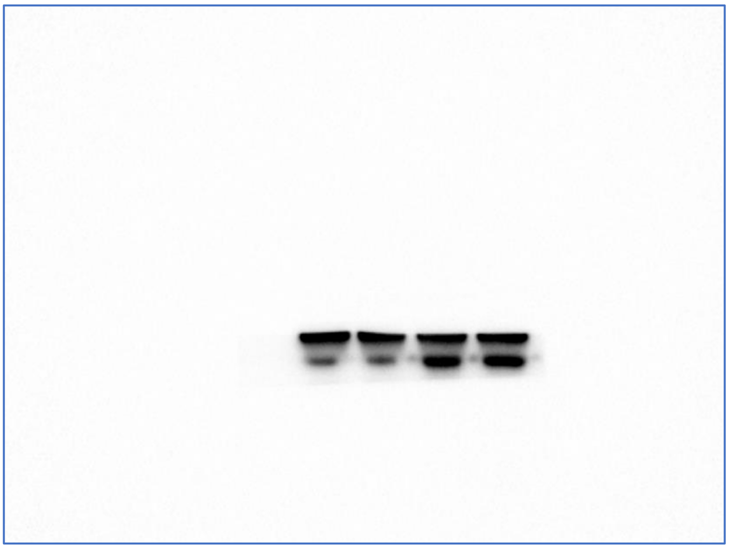

Fig.2a – GSDMD- Repeat1

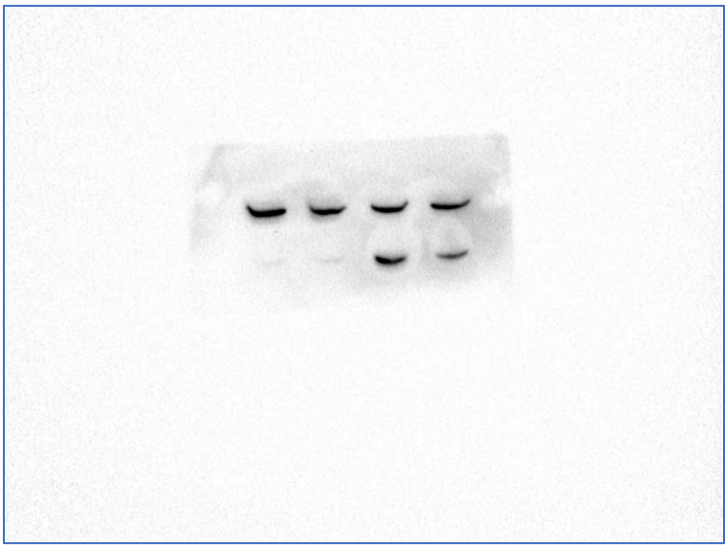

Fig.2a – GSDMD- Repeat2

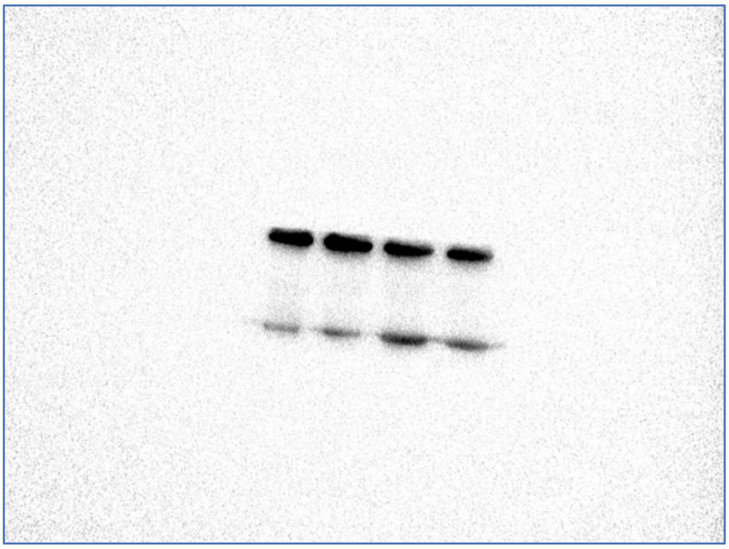

Fig.2a – GSDMD- Repeat3

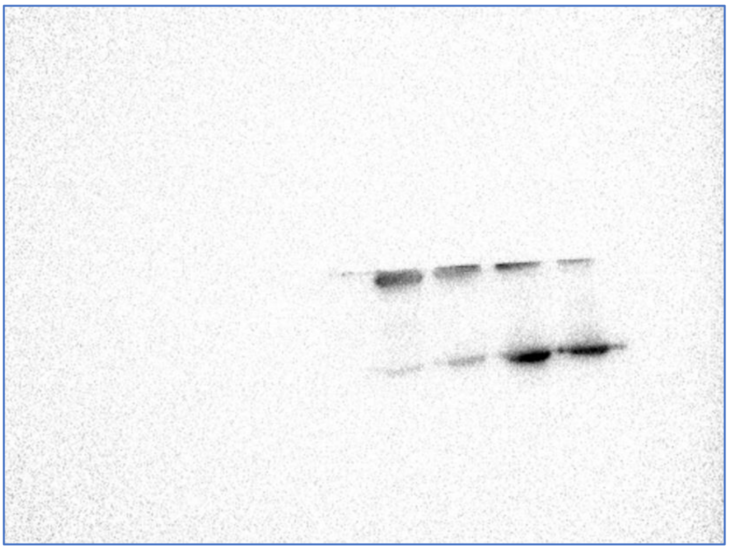

Fig.2a – GAPDH - Repeat1

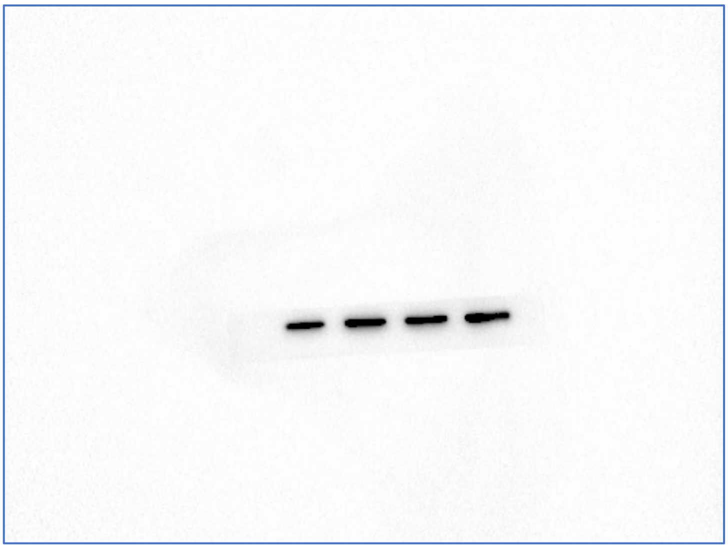

Fig.2a – GAPDH – Repeat2

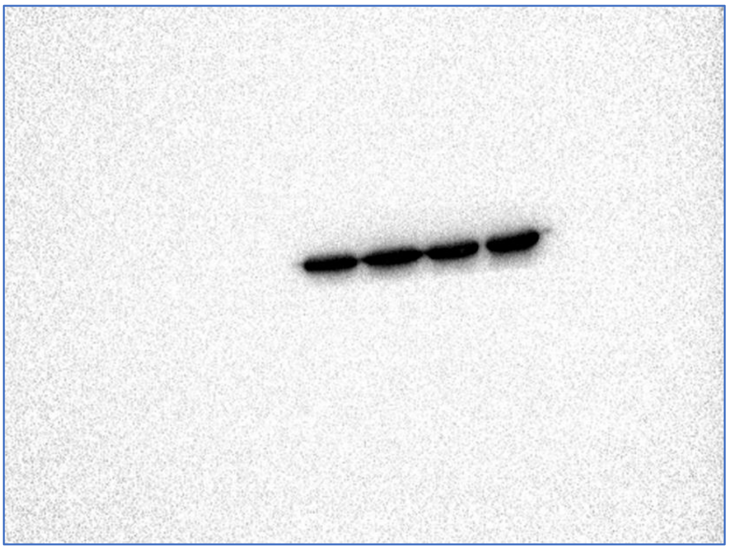

Fig.2a – GAPDH – Repeat3

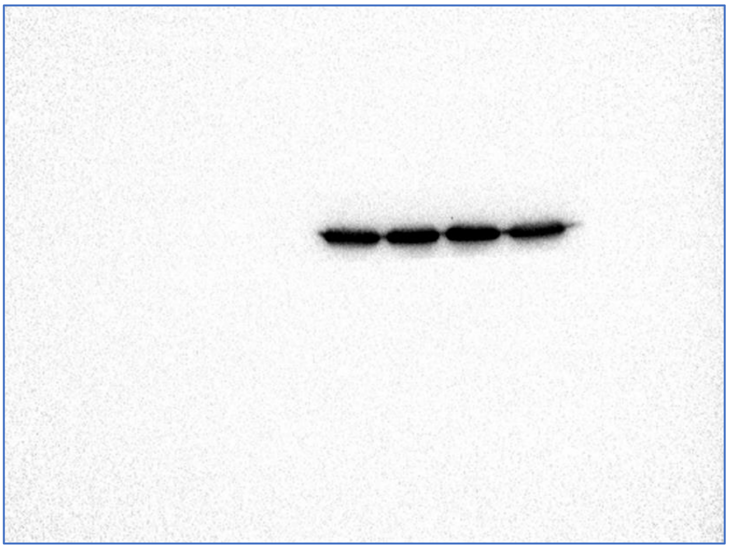

Fig.2n – CASP-1 - Repeat1

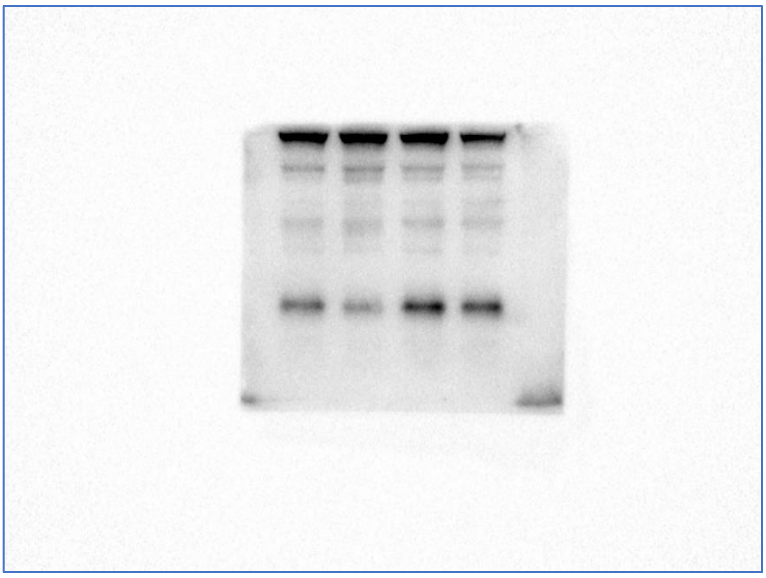

Fig.2n – CASP-1 – Repeat2

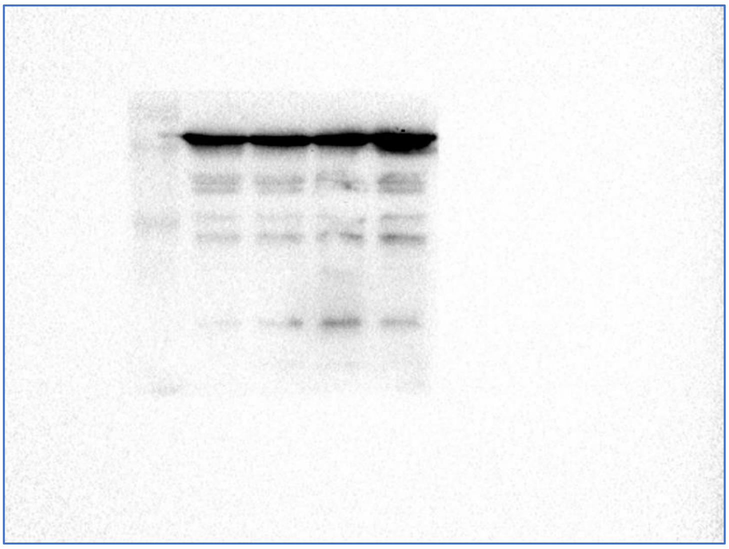

Fig.2n – CASP-1 – Repeat3

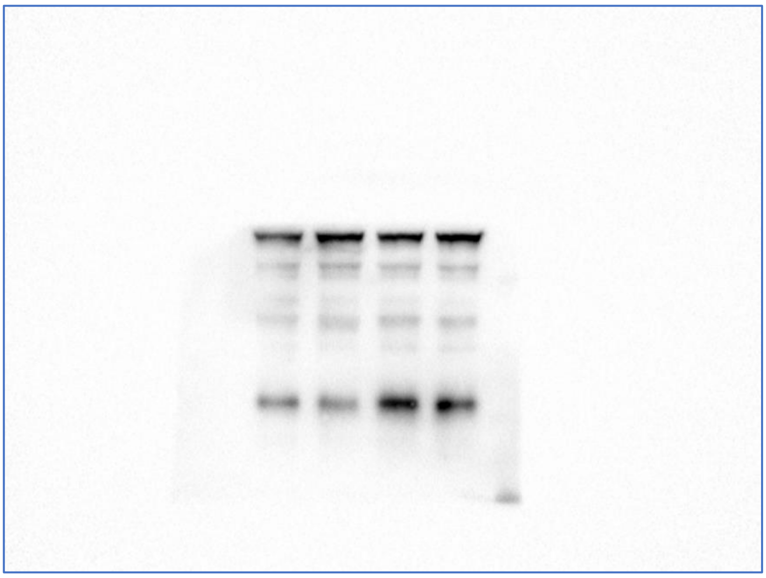

Fig.2n – IL-18- Repeat1

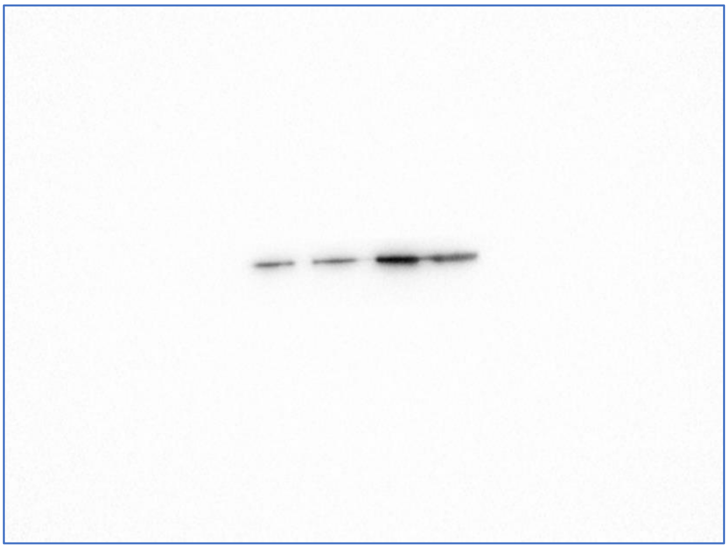

Fig.2n – IL-18- Repeat2

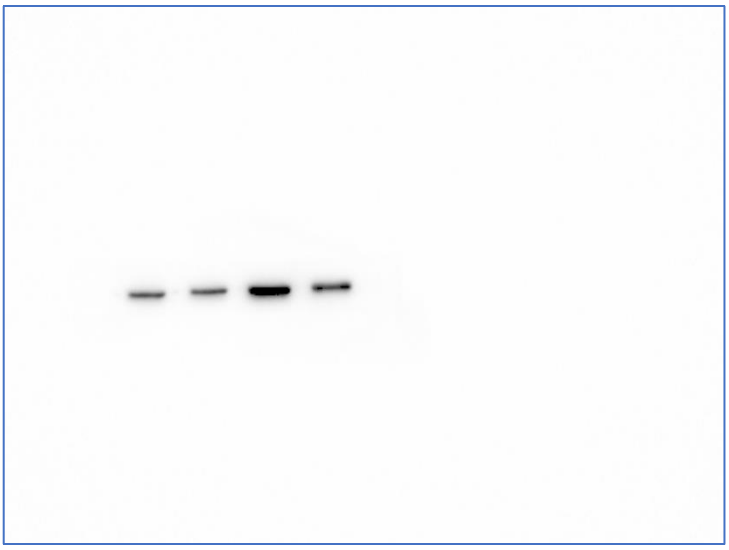

Fig.2n – IL-18- Repeat3

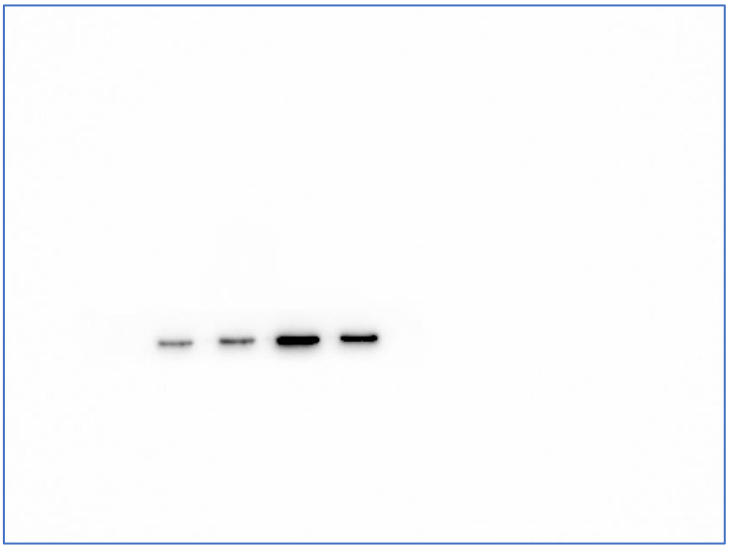

Fig.2n – IL-1β- Repeat1

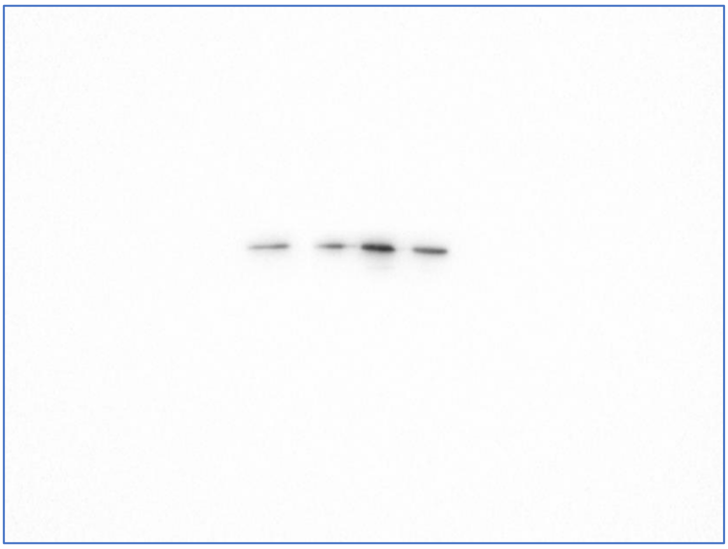

Fig.2n – IL-1β- Repeat2

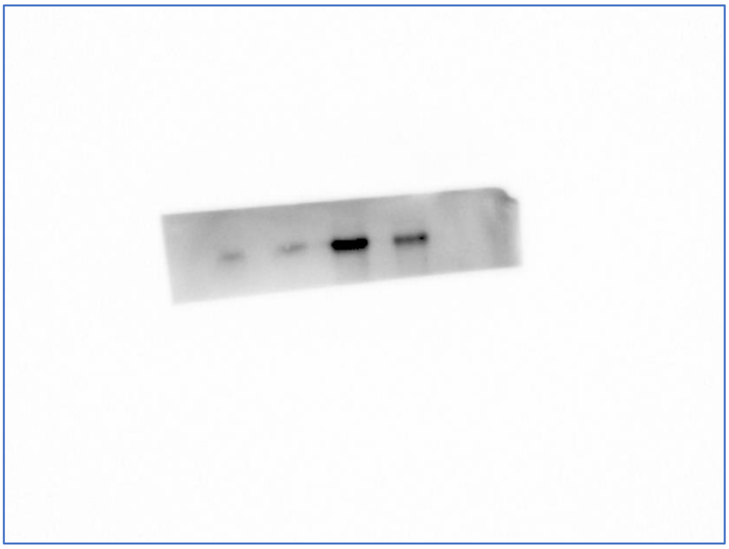

Fig.2n – IL-1β- Repeat3

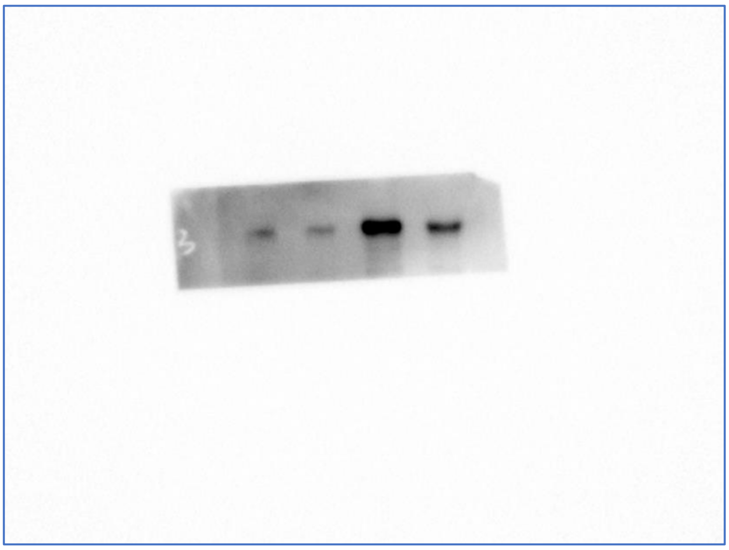

Fig.2n – GAPDH - Repeat1

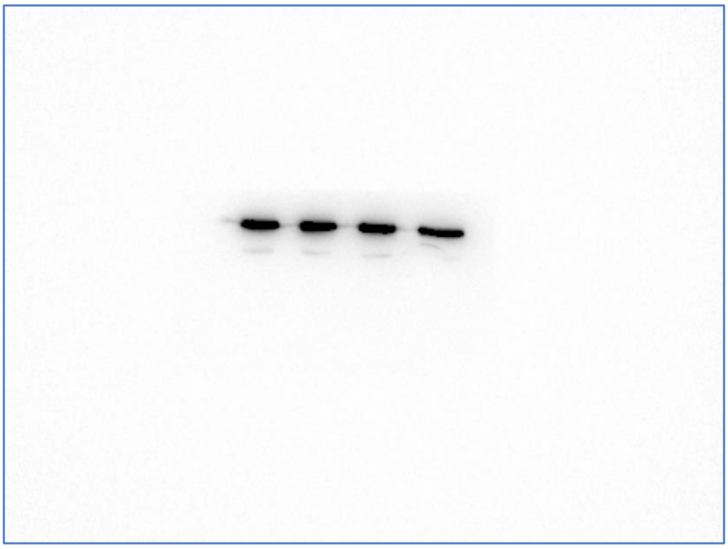

Fig.2n – GAPDH – Repeat2

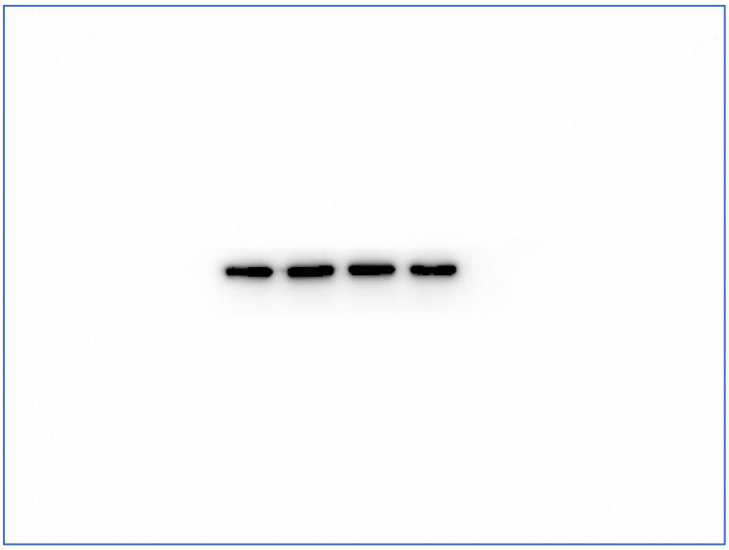

Fig.2n – GAPDH – Repeat3

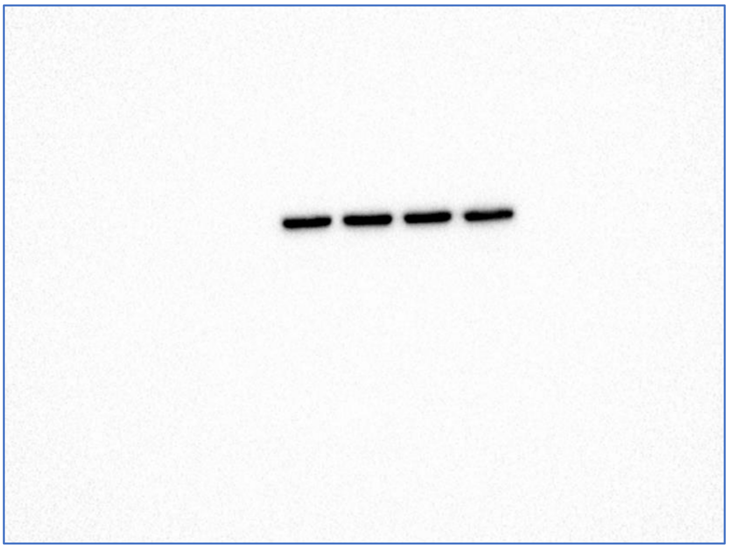

Fig.3a – AIM2-IP - Repeat1

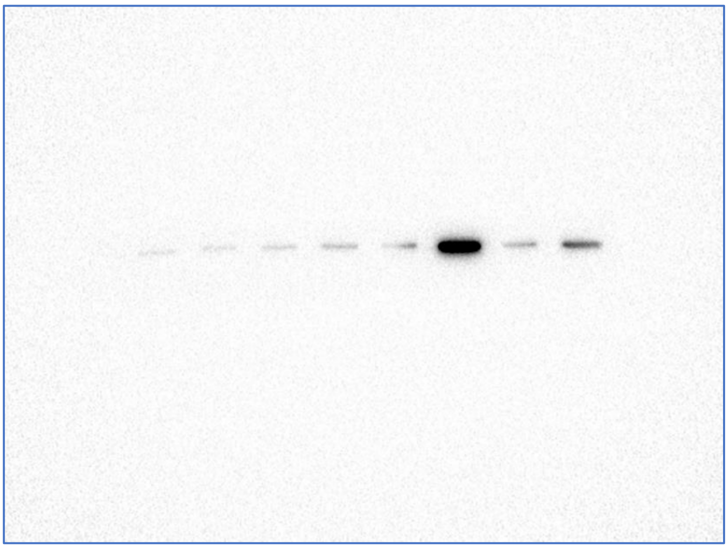

Fig.3a – AIM2-IP – Repeat2

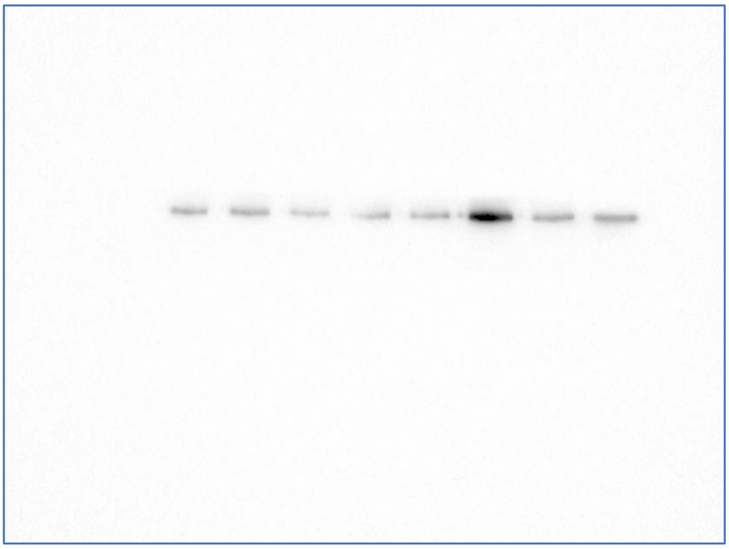

Fig.3a – AIM2-IP – Repeat3

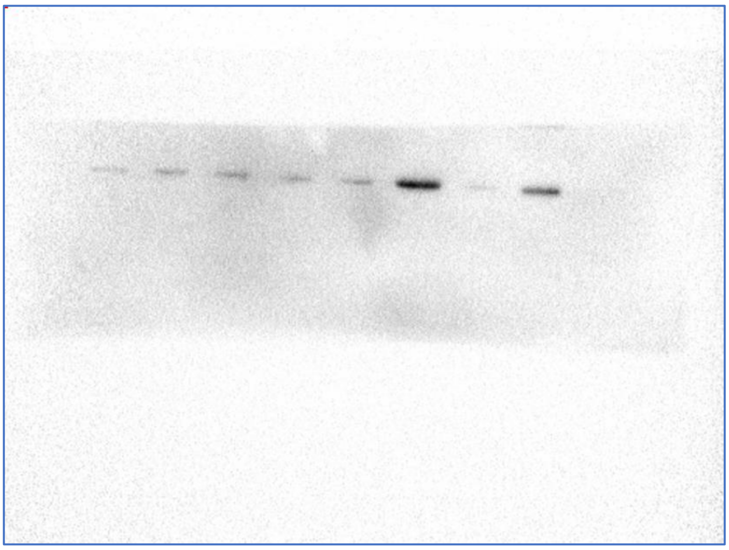

Fig.3a – NLRP3-IP - Repeat1

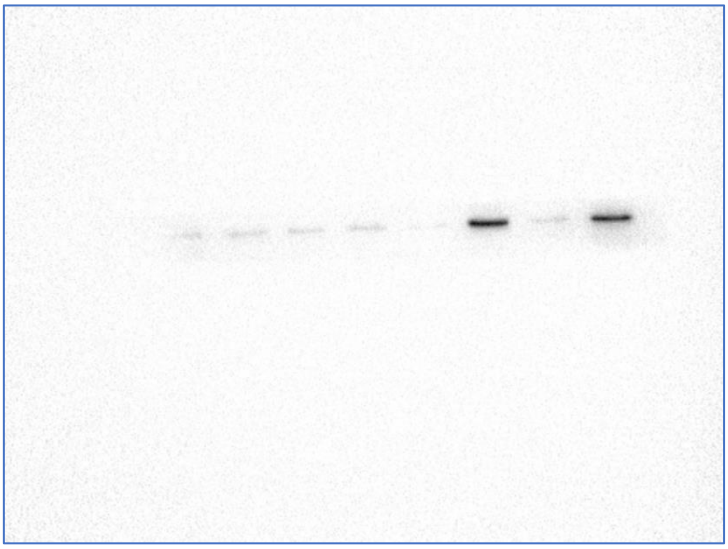

Fig.3a – NLRP3-IP – Repeat2

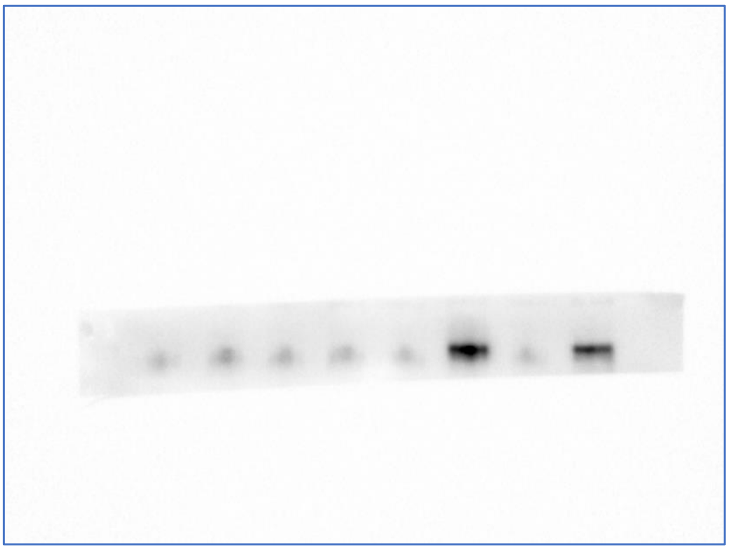

Fig.3a – NLRP3-IP – Repeat3

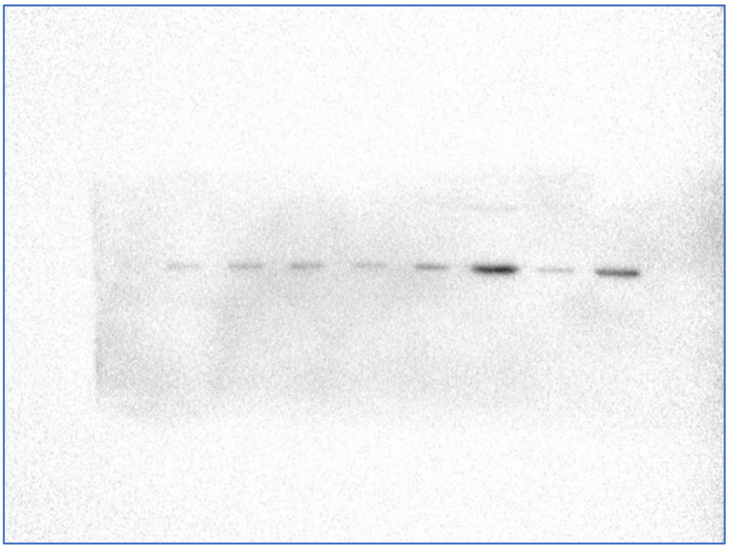

Fig.3a – AIM2-input - Repeat1

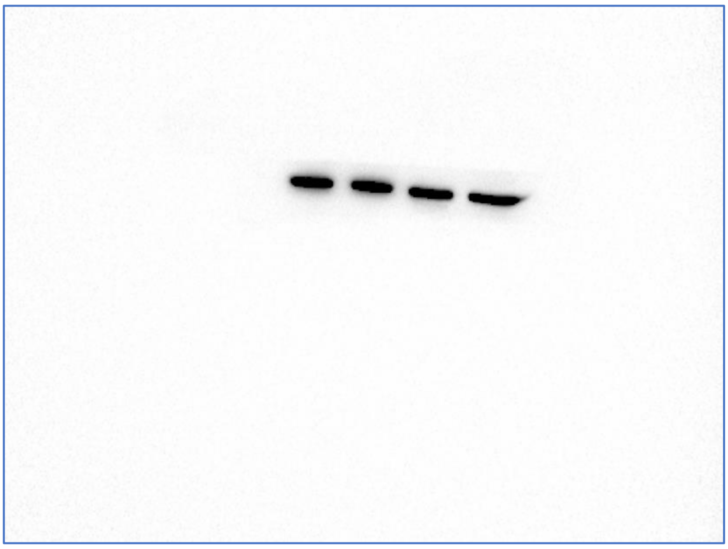

Fig.3a – AIM2-input – Repeat2

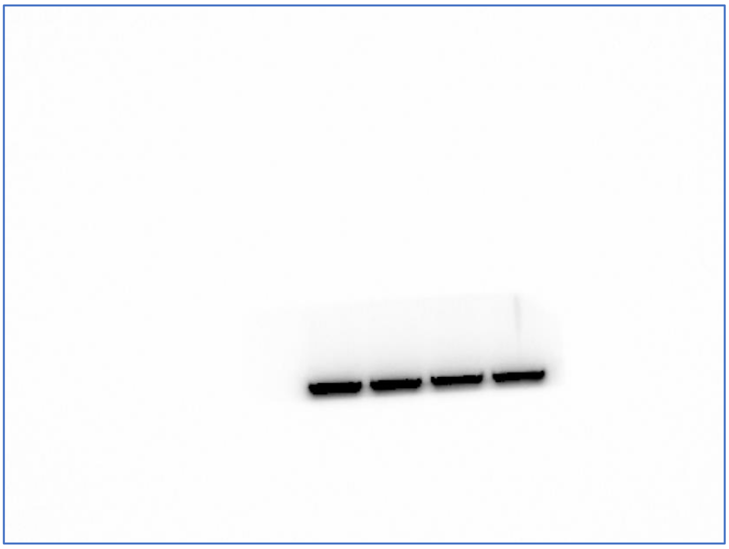

Fig.3a – AIM2-input – Repeat3

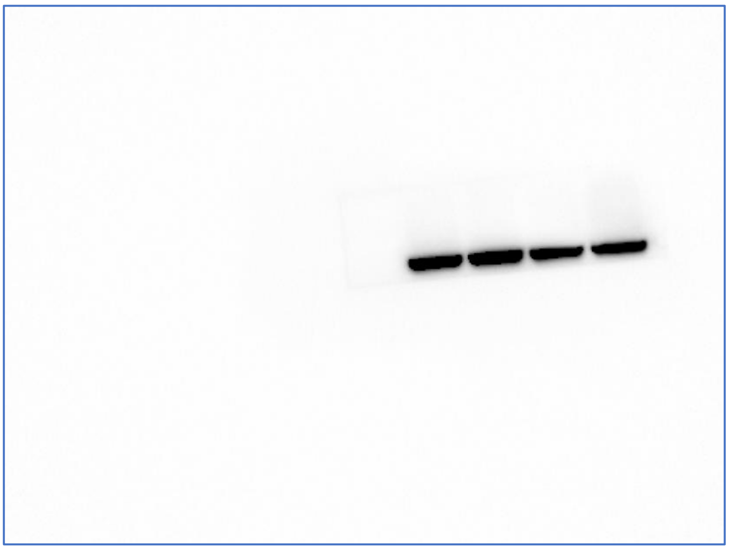

Fig.3a – NLRP3-input - Repeat1

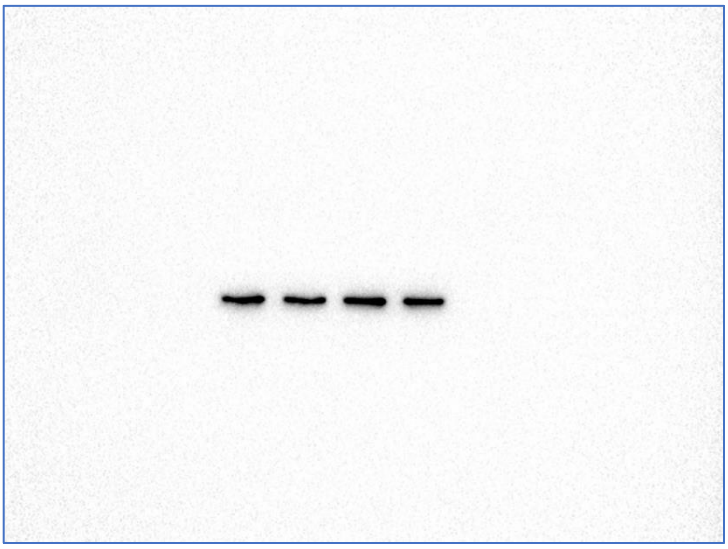

Fig.3a – NLRP3-input – Repeat2

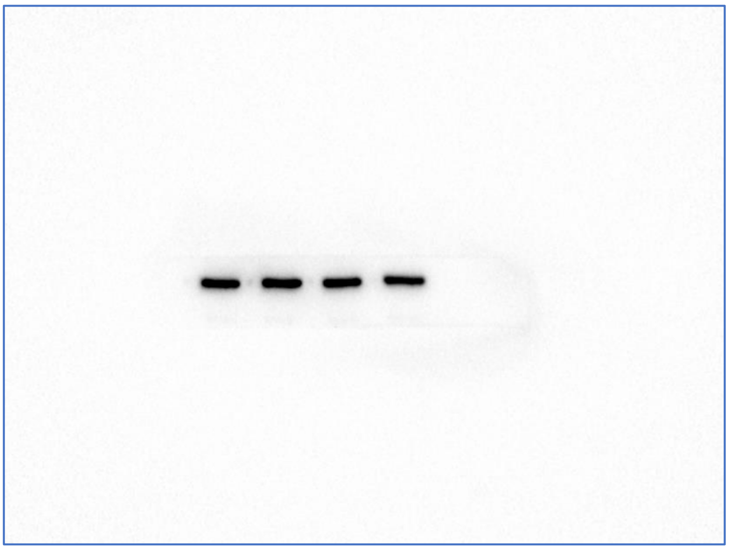

Fig.3a – NLRP3-input – Repeat3

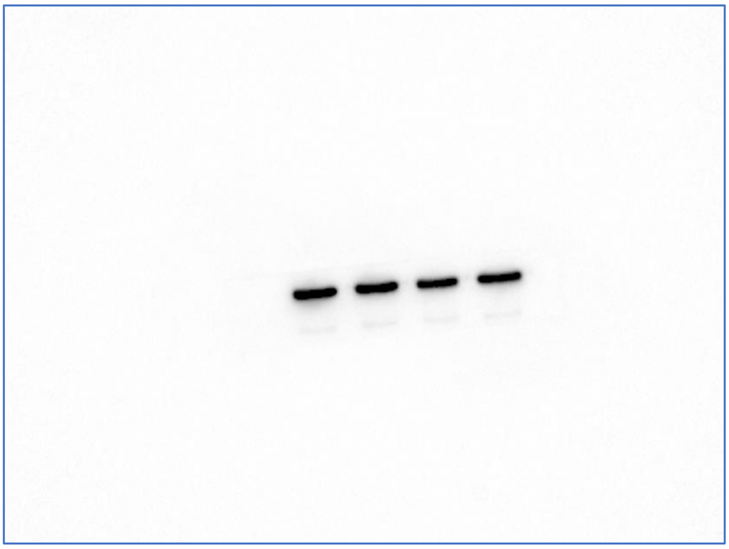

Fig.4a – AIM2-IP - Repeat1

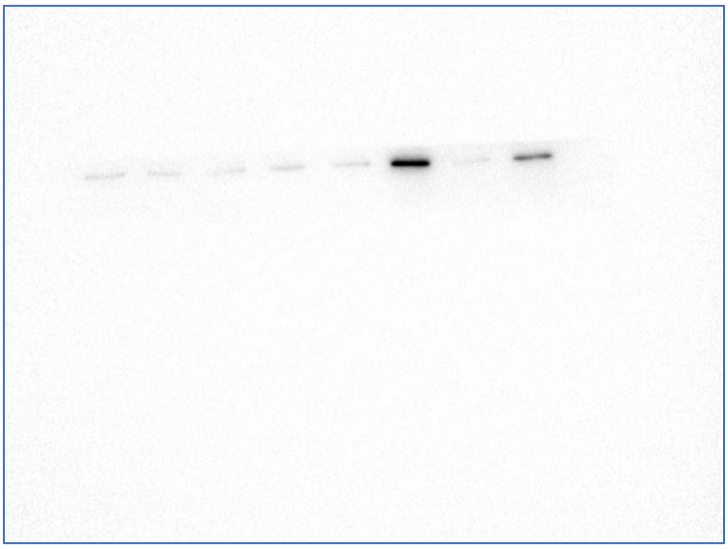

Fig.4a – AIM2-IP – Repeat2

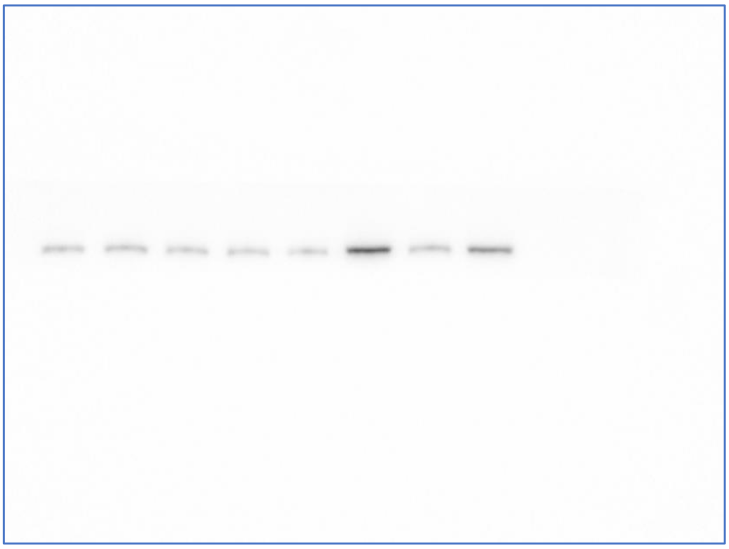

Fig.4a – AIM2-IP – Repeat3

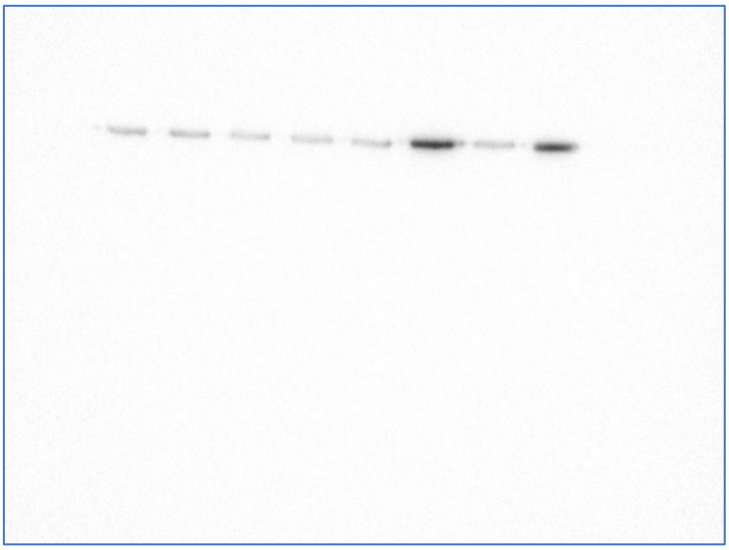

Fig.4a – NLRP3-IP - Repeat1

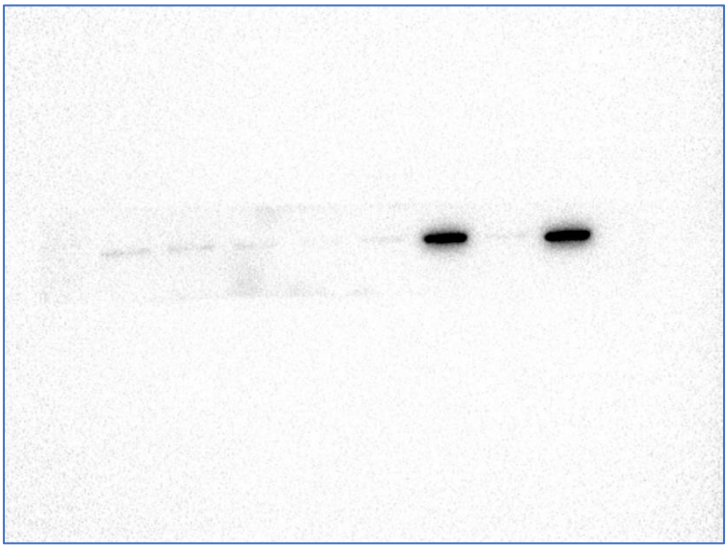

Fig.4a – NLRP3-IP – Repeat2

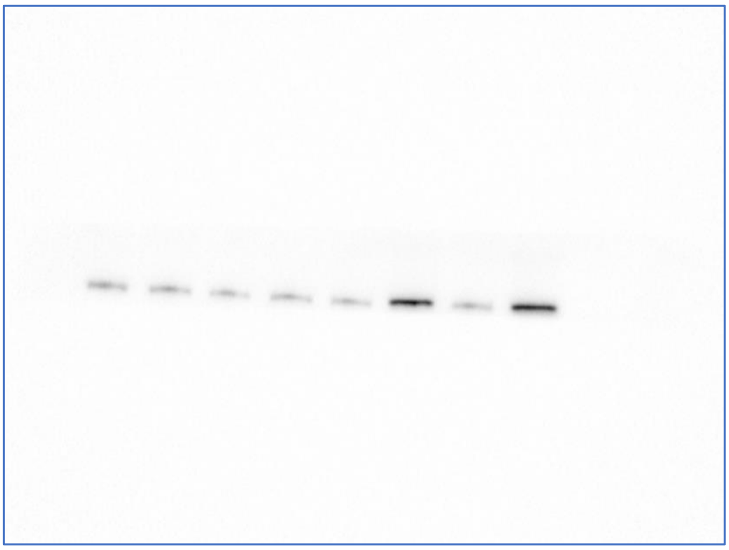

Fig.4a – NLRP3-IP – Repeat3

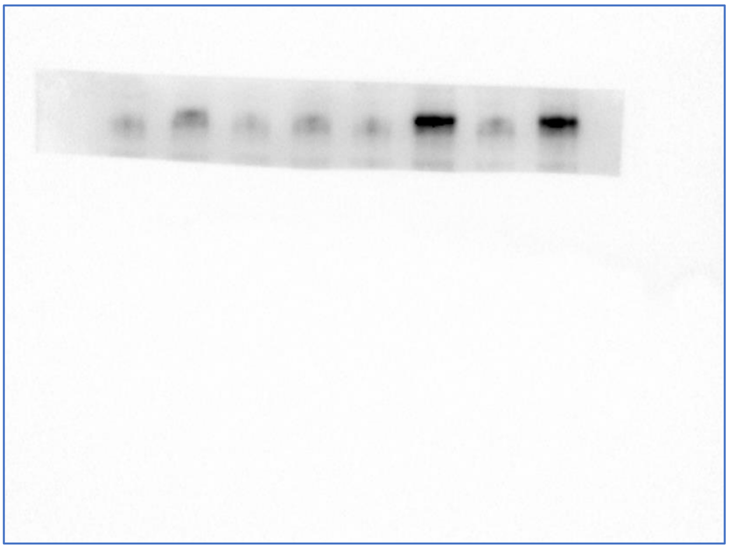

Fig.4a – AIM2-input - Repeat1

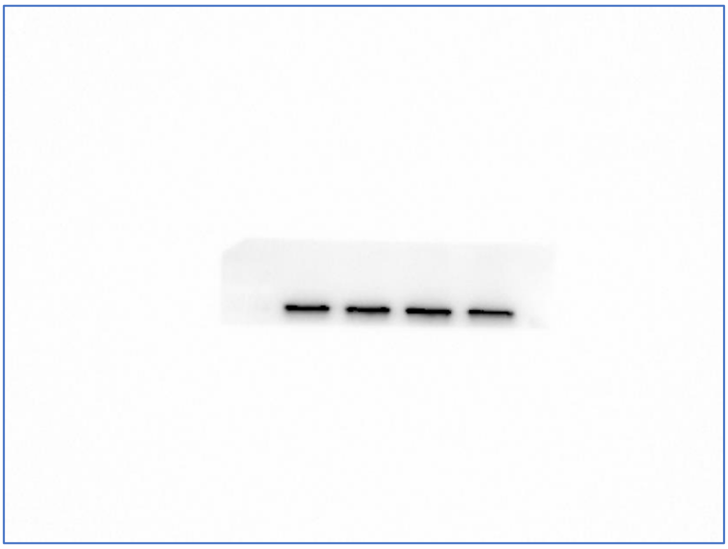

Fig.4a – AIM2-input – Repeat2

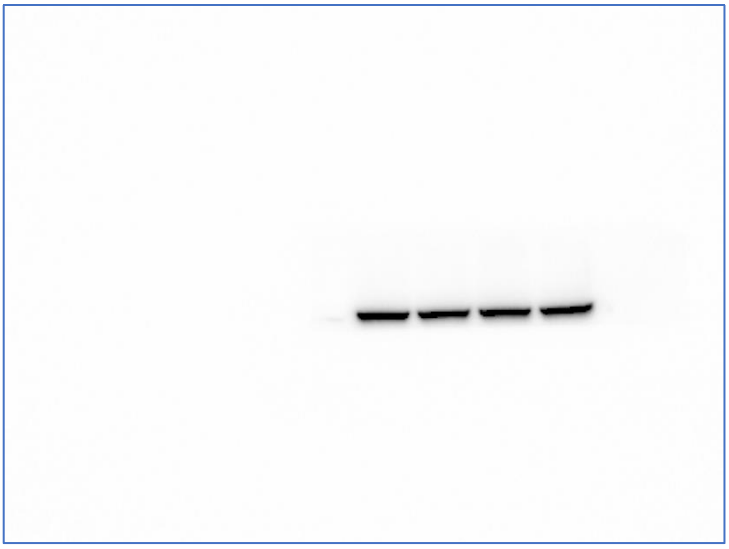

Fig.4a – AIM2-input – Repeat3

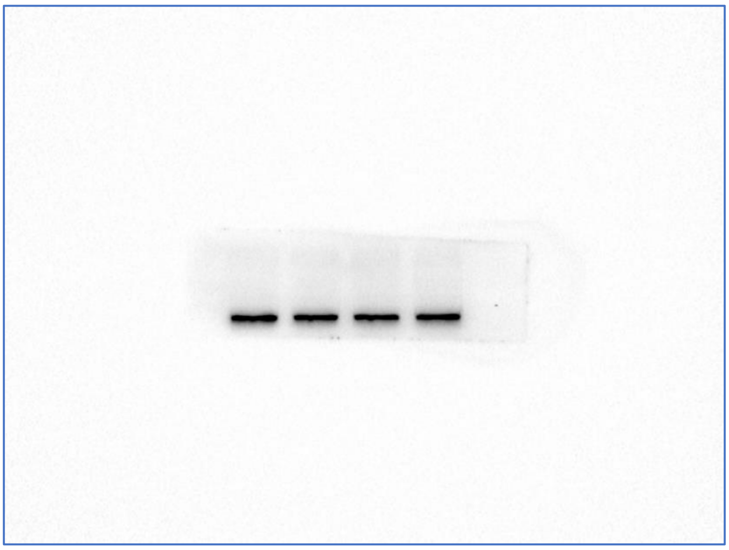

Fig.4a – NLRP3-input - Repeat1

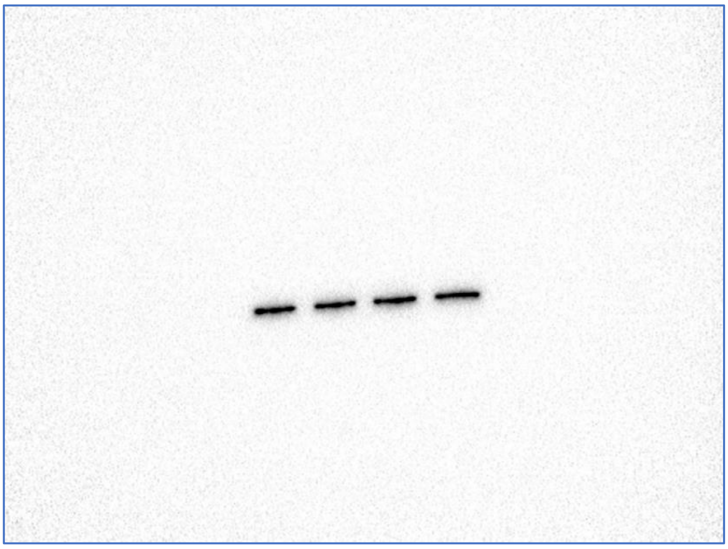

Fig.4a – NLRP3-input – Repeat2

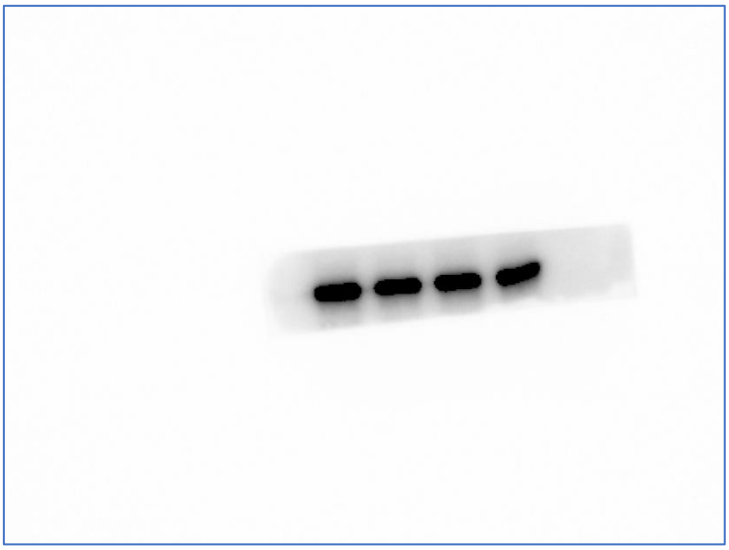

Fig.4a – NLRP3-input – Repeat3

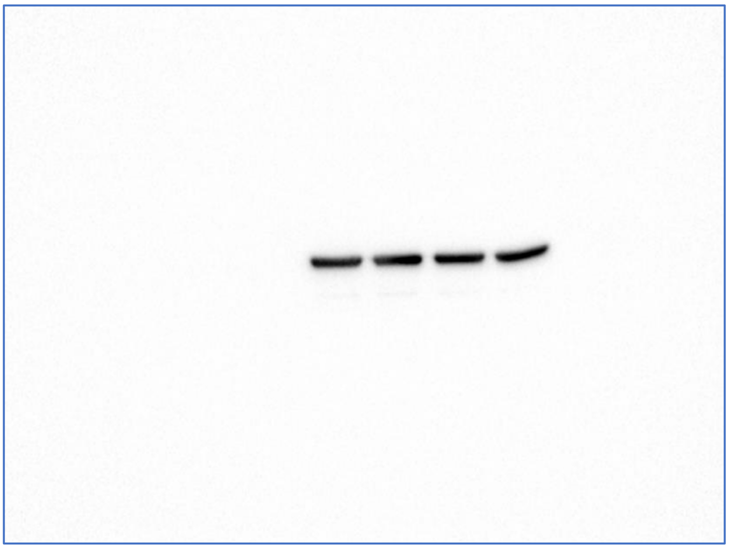

Fig.5a – γH2AX - Repeat1

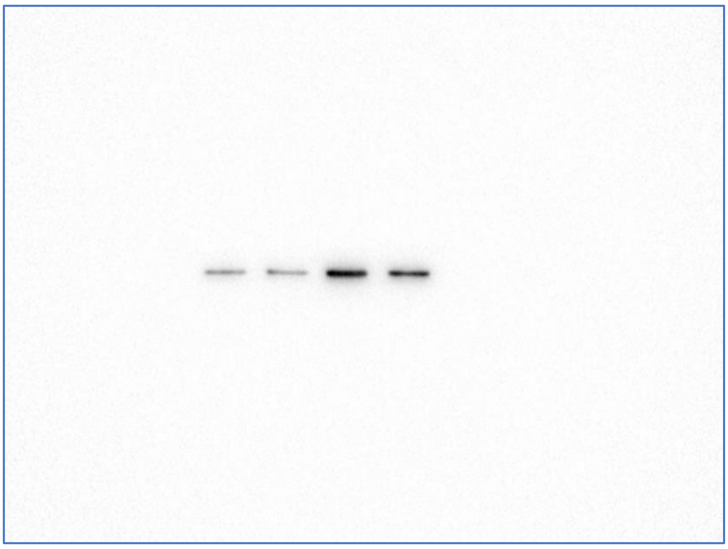

Fig.5a – γH2AX – Repeat2

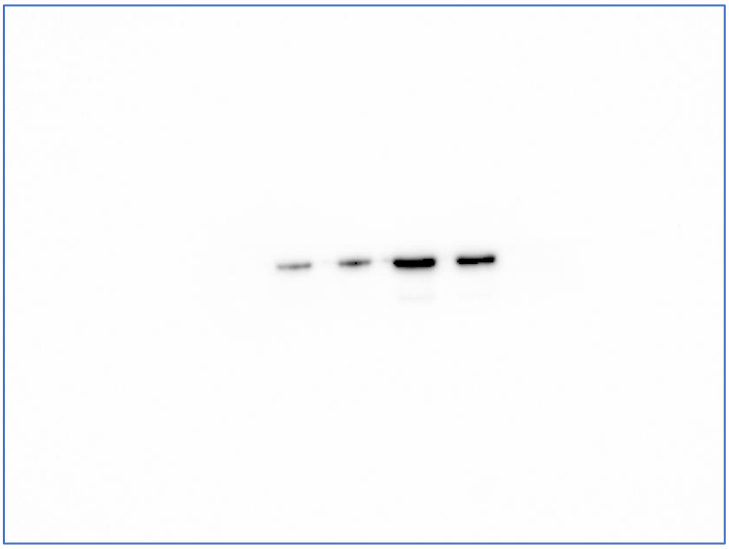

Fig.5a – γH2AX – Repeat3

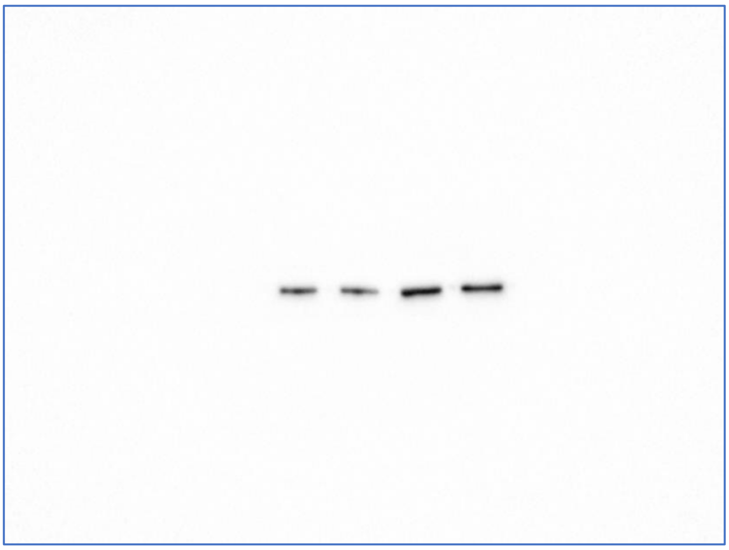

Fig.5a – GAPDH - Repeat1

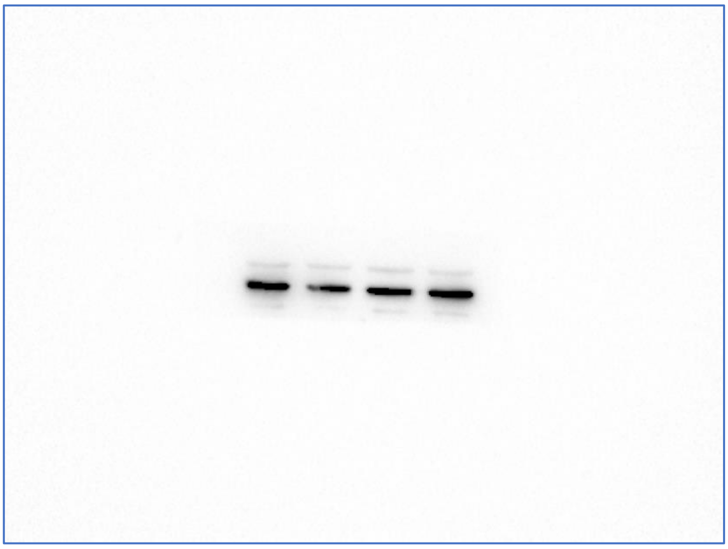

Fig.5a – GAPDH – Repeat2

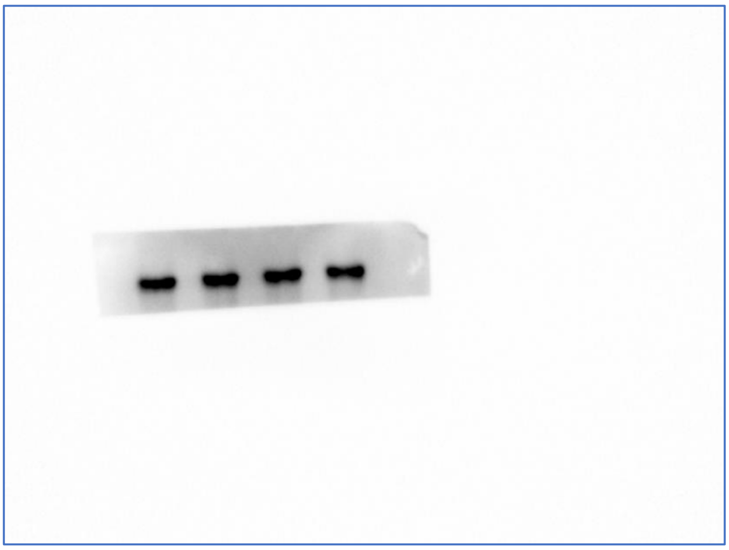

Fig.5a – GAPDH – Repeat3

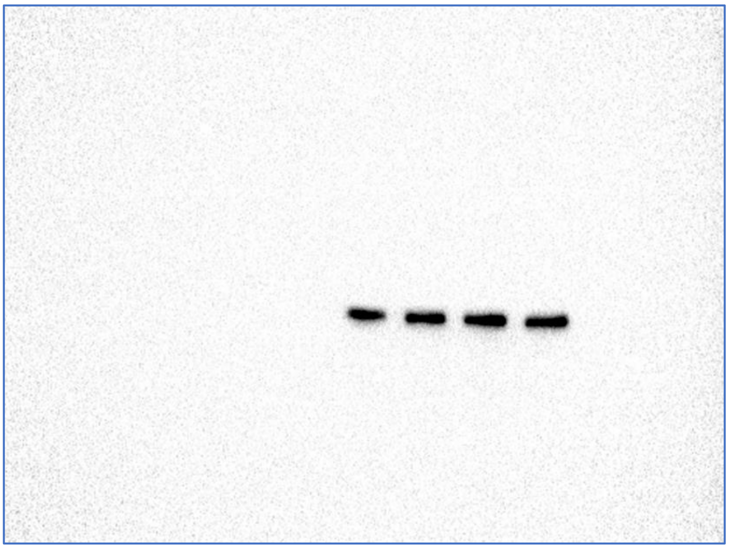

Fig.5c – P53BP1 - Repeat1

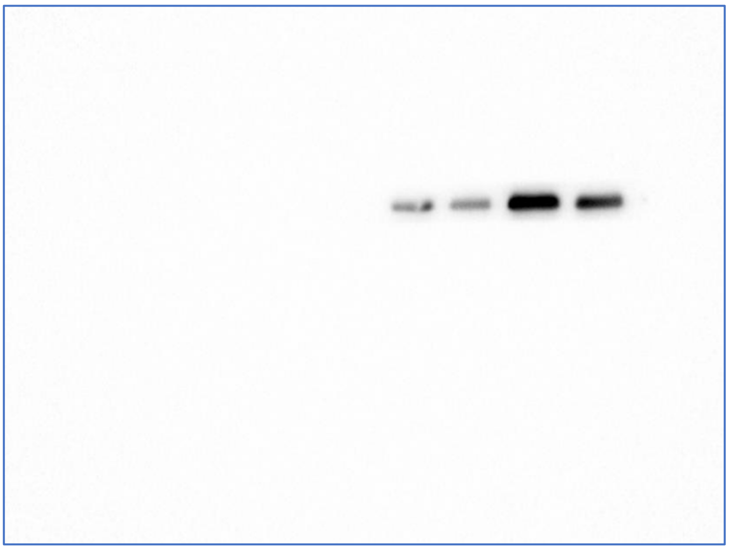

Fig.5c – P53BP1 – Repeat2

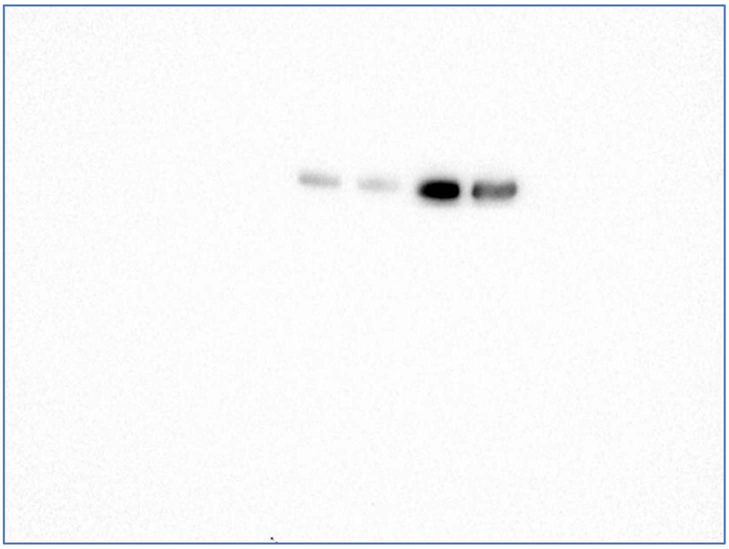

Fig.5c – P53BP1 – Repeat3

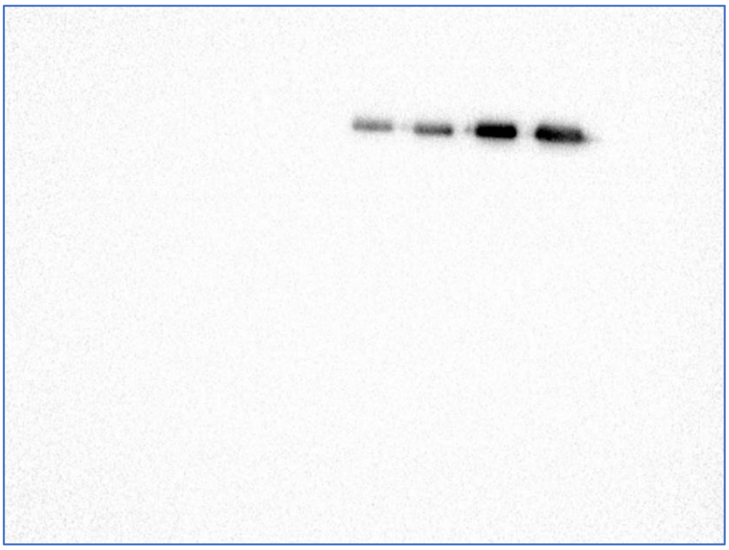

Fig.5c – GAPDH - Repeat1

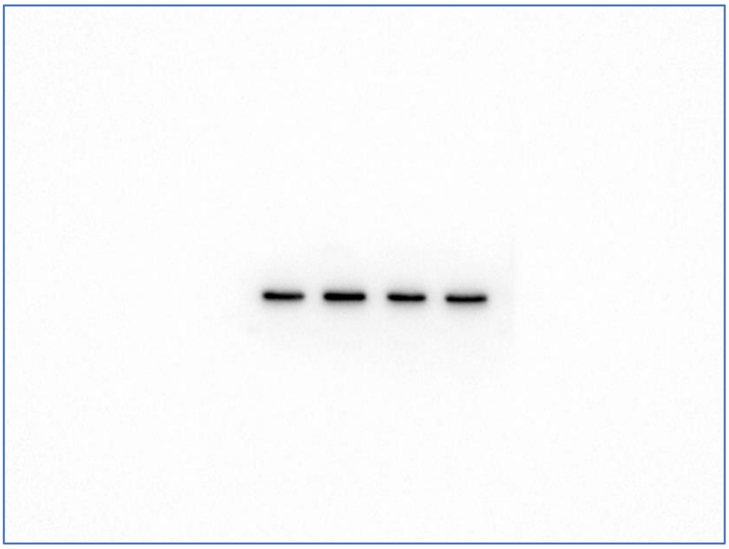

Fig.5c – GAPDH – Repeat2

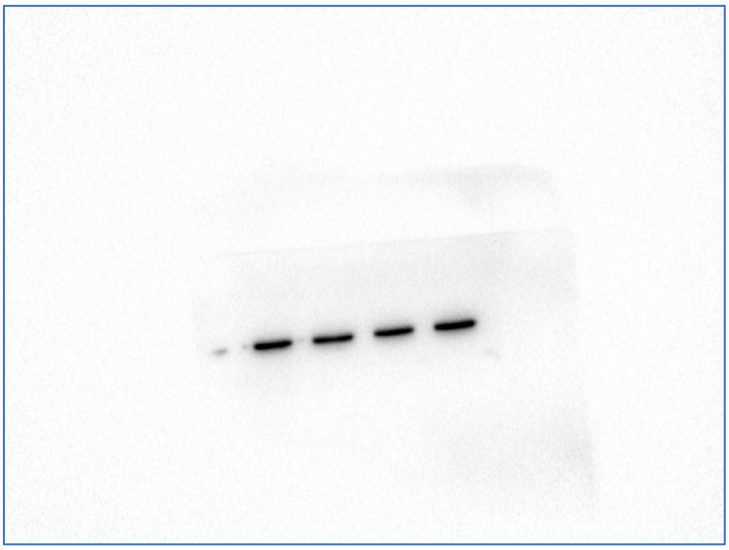

Fig.5c – GAPDH – Repeat3

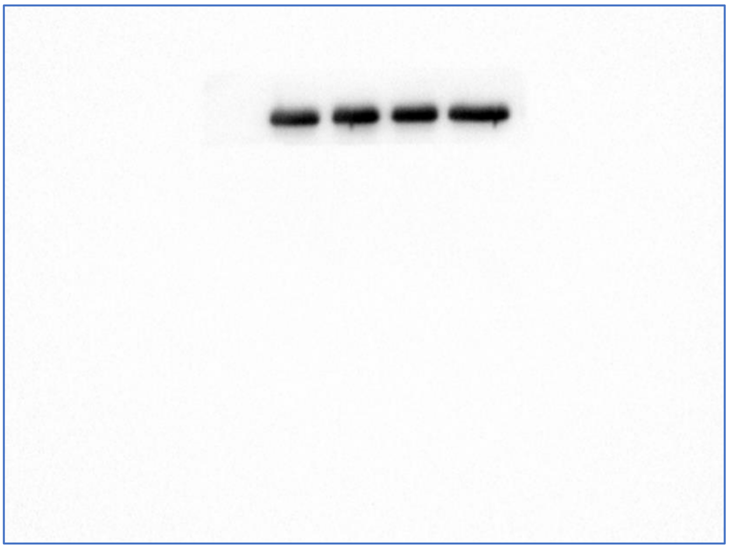

Fig.5h – γH2AX - Repeat1

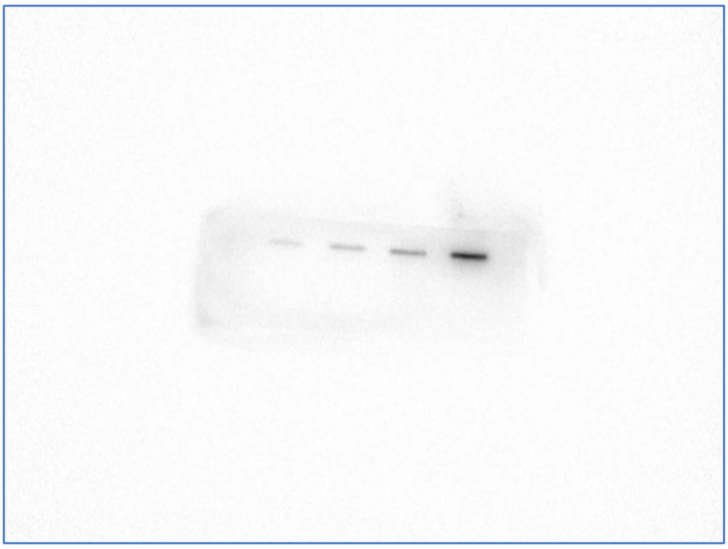

Fig.5h – γH2AX – Repeat2

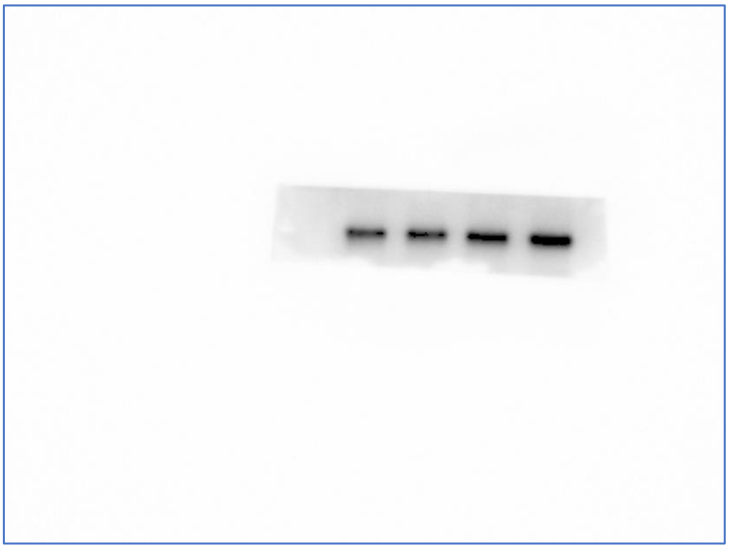

Fig.5h – γH2AX – Repeat3

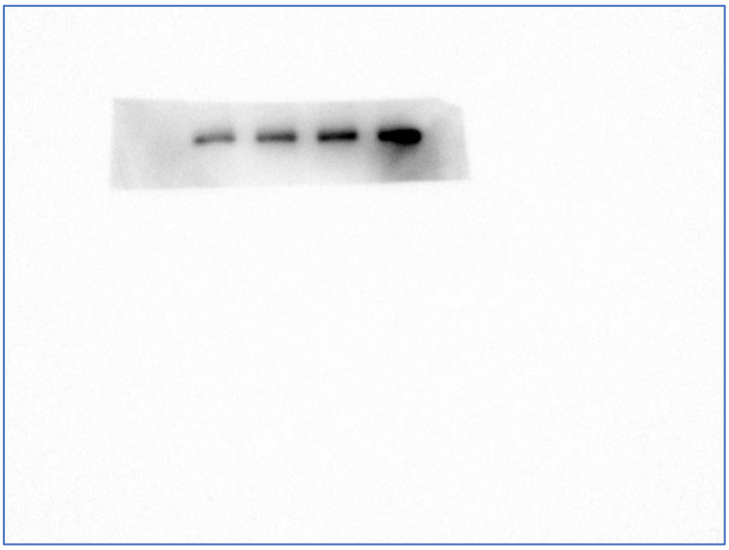

Fig.5h – GAPDH - Repeat1

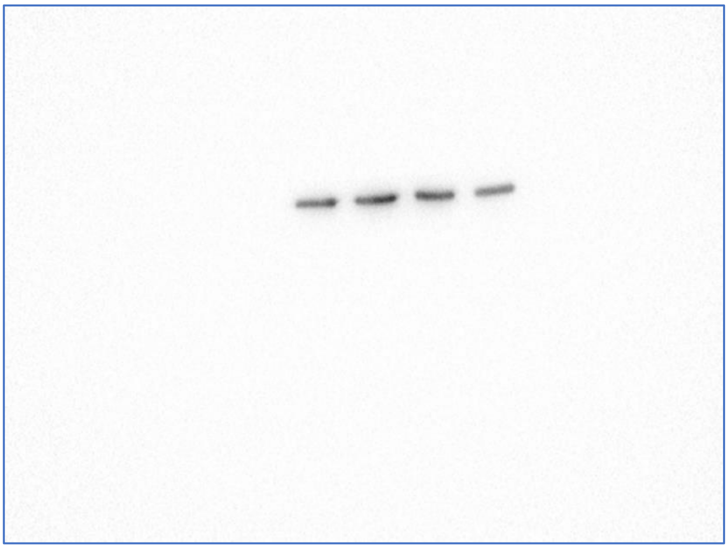

Fig.5h – GAPDH – Repeat2

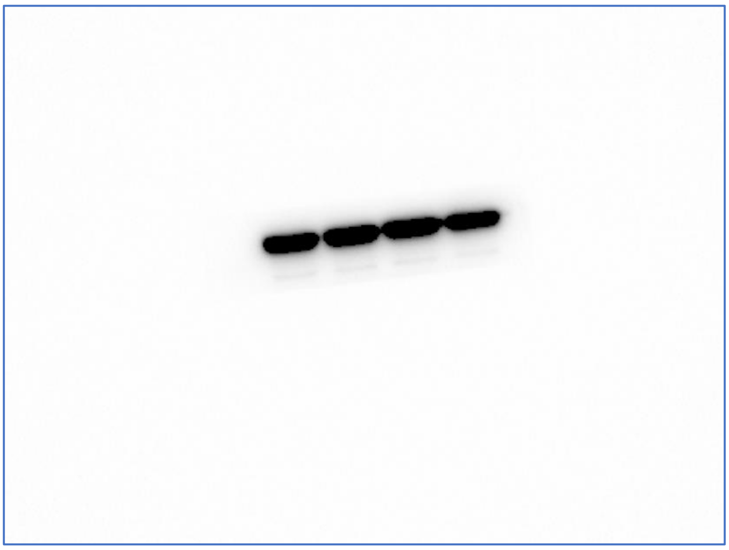

Fig.5h – GAPDH – Repeat3

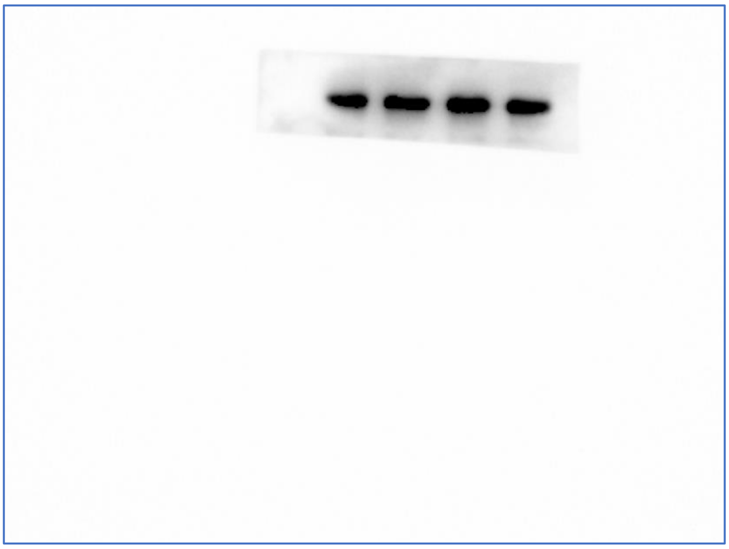

Fig.5j – P53BP1 - Repeat1

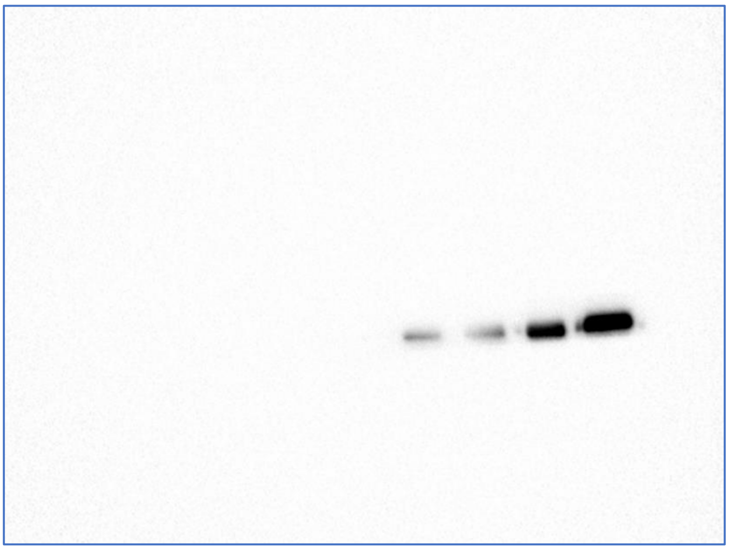

Fig.5j – P53BP1 – Repeat2

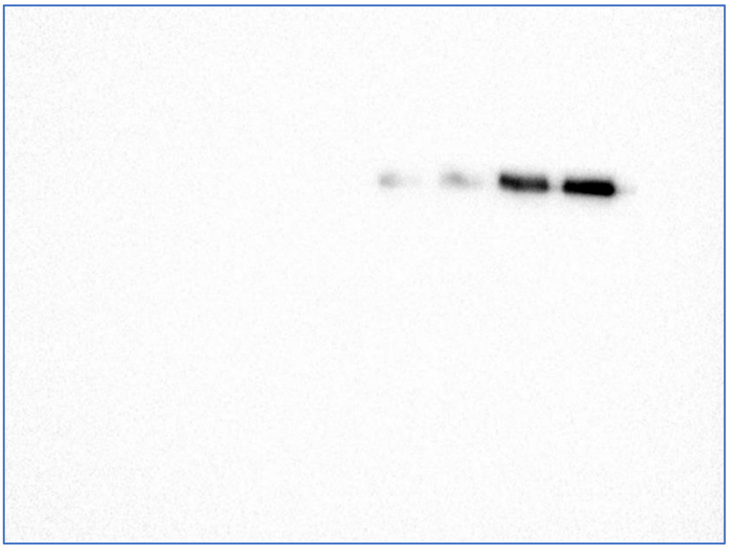

Fig.5j – P53BP1 – Repeat3

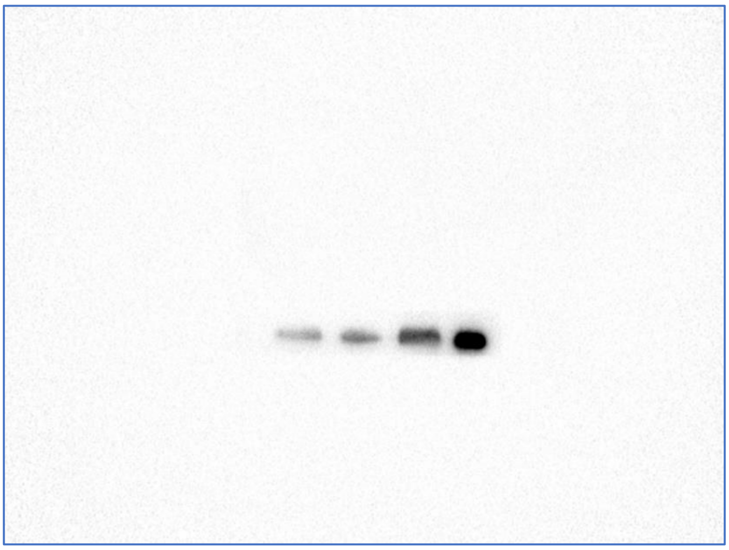

Fig.5j – GAPDH - Repeat1

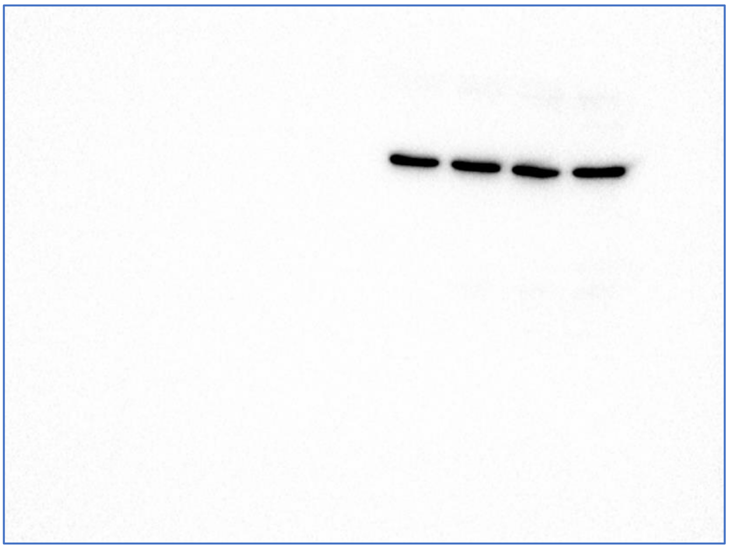

Fig.5j – GAPDH – Repeat2

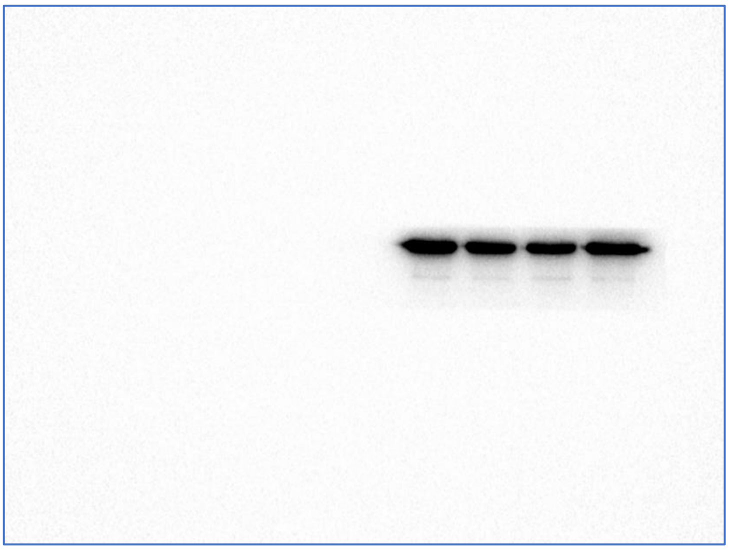

Fig.5j – GAPDH – Repeat3

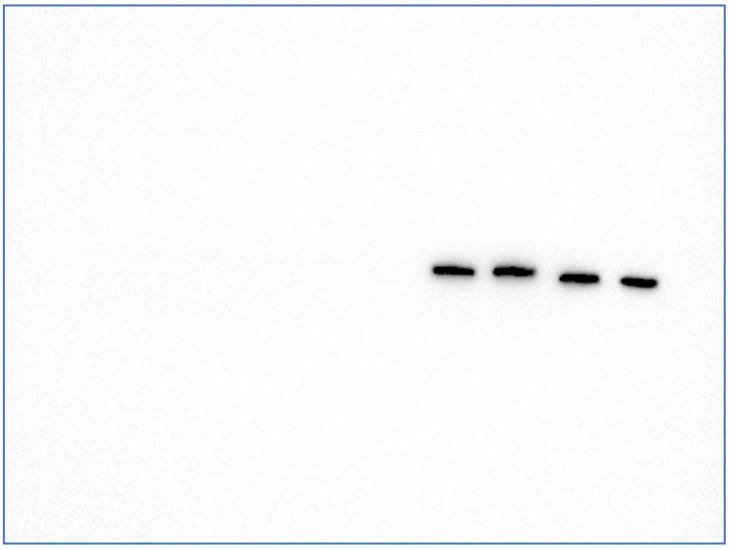

Fig.6e – CASP-1 - Repeat1

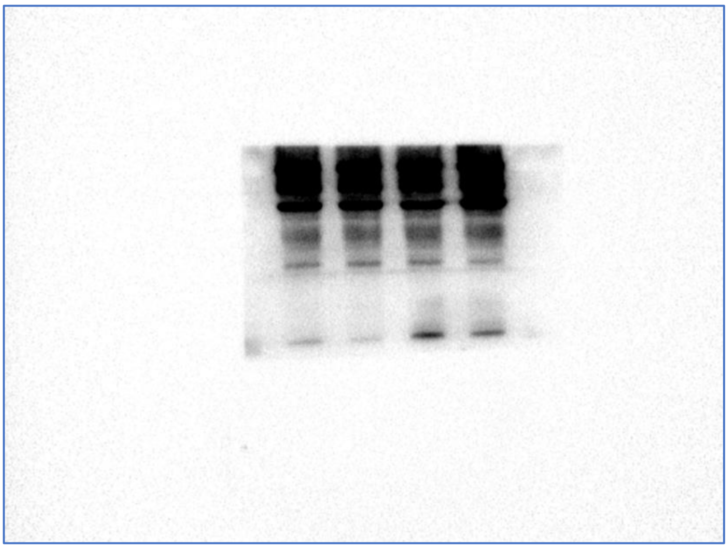

Fig.6e – CASP-1 – Repeat2

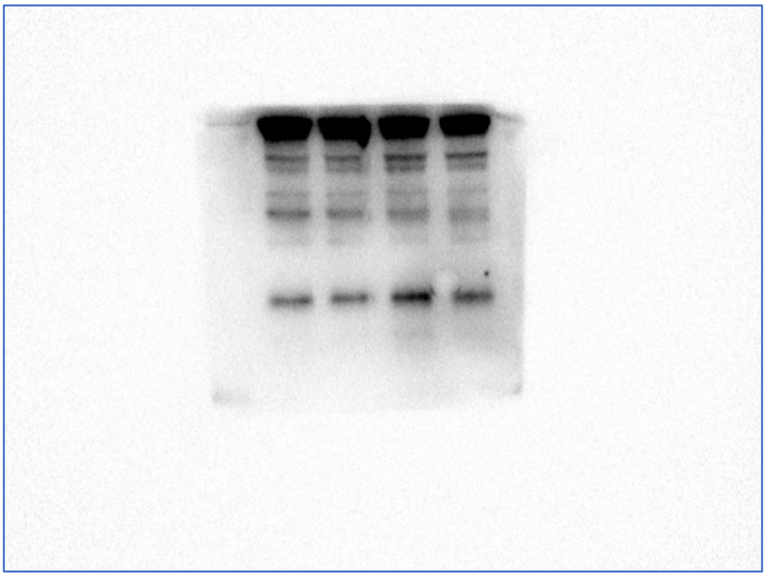

Fig.6e – CASP-1 – Repeat3

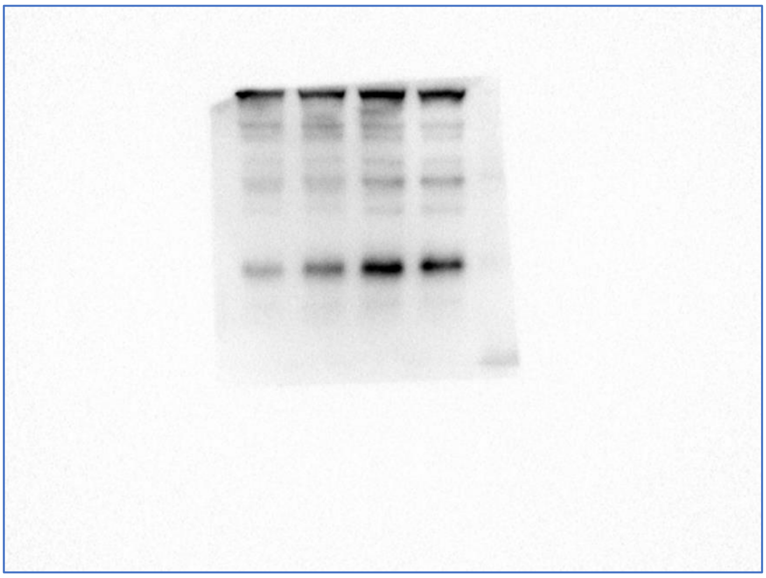

Fig. 6e – IL-18 - Repeat1

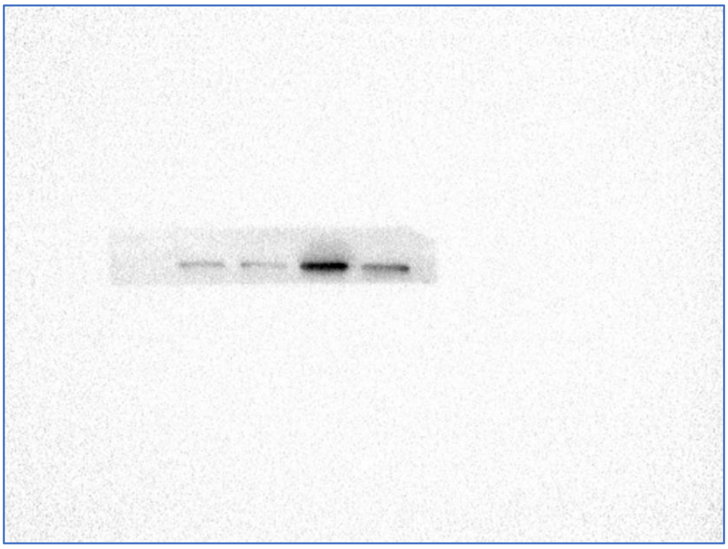

Fig. 6e – IL-18 – Repeat2

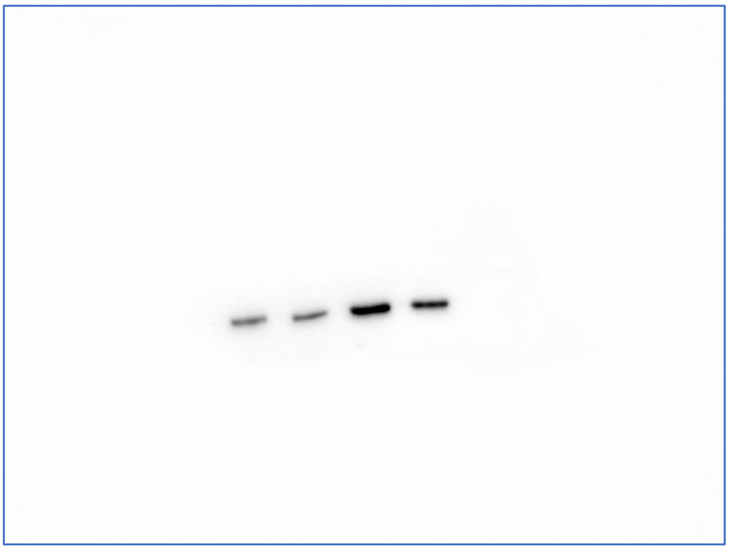

Fig. 6e – IL-18 – Repeat3

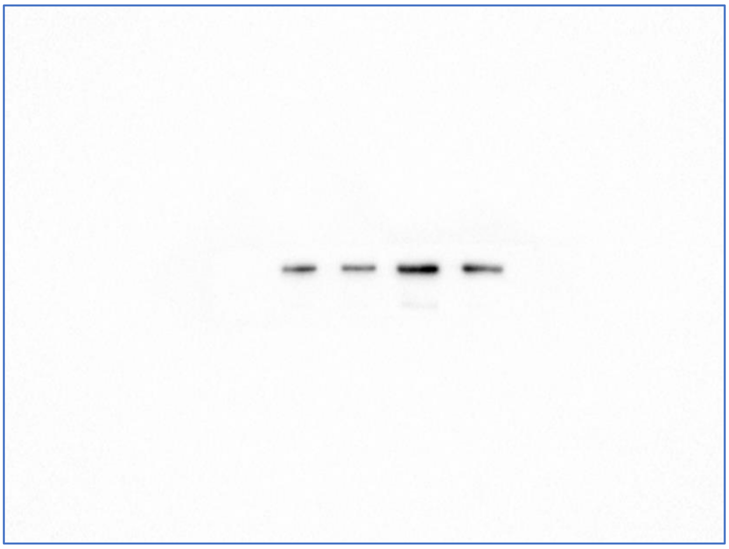

Fig. 6e – IL-1β- Repeat1

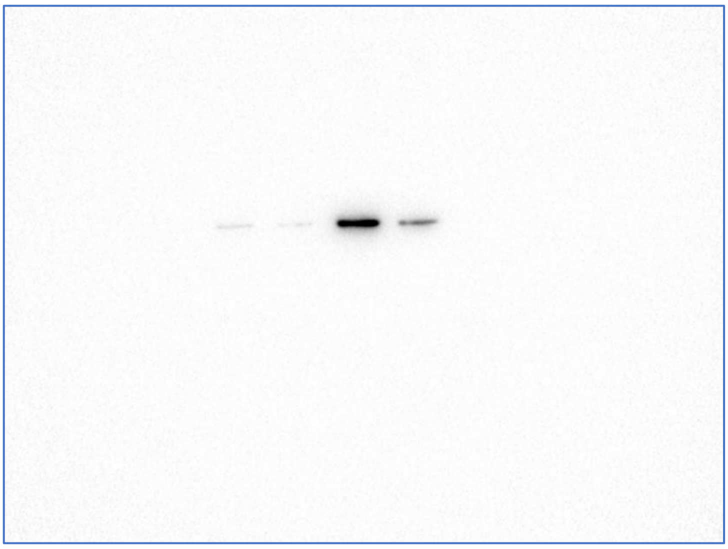

Fig. 6e – IL-1β- Repeat2

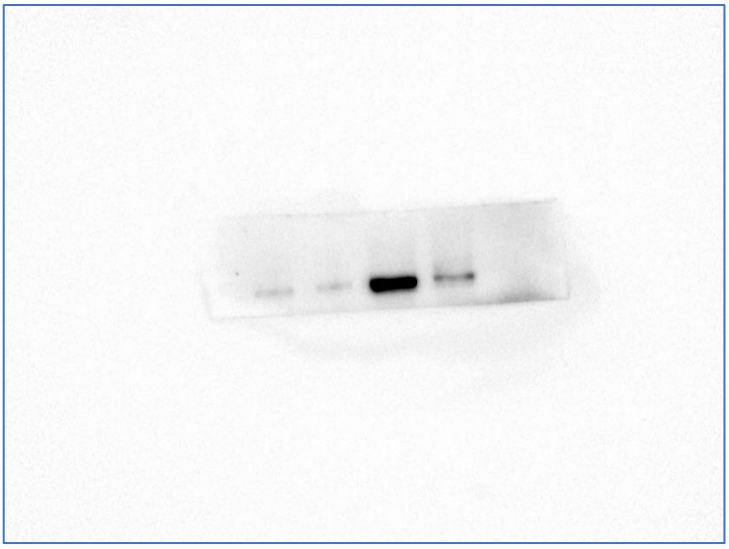

Fig. 6e – IL-1β- Repeat3

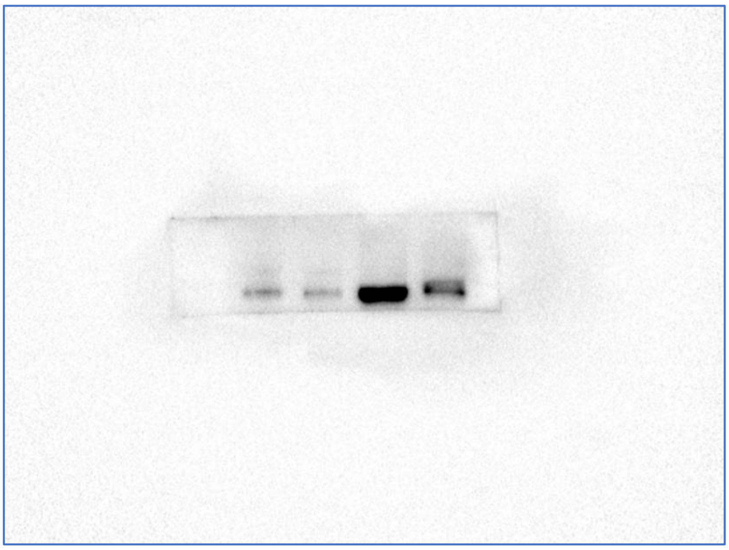

Fig. 6e – γH2AX - Repeat1

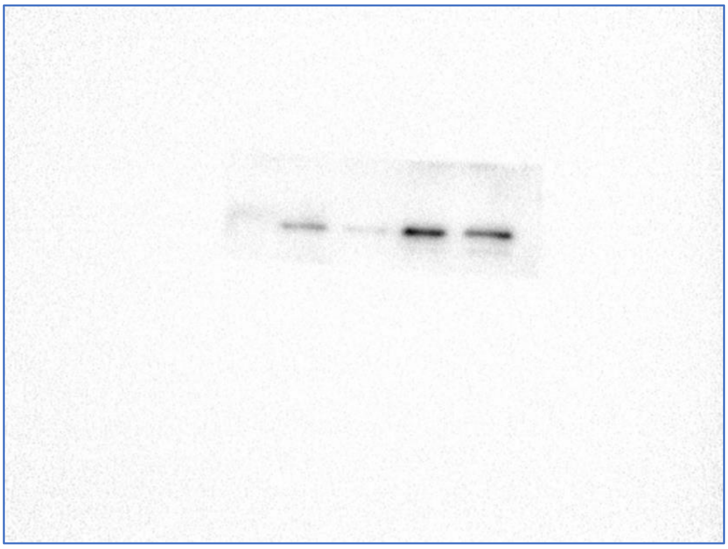

Fig. 6e – γH2AX – Repeat2

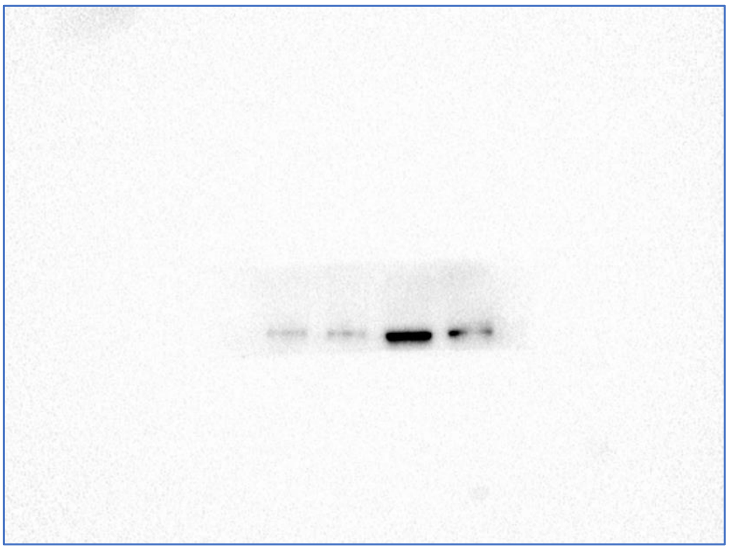

Fig. 6e – γH2AX – Repeat3

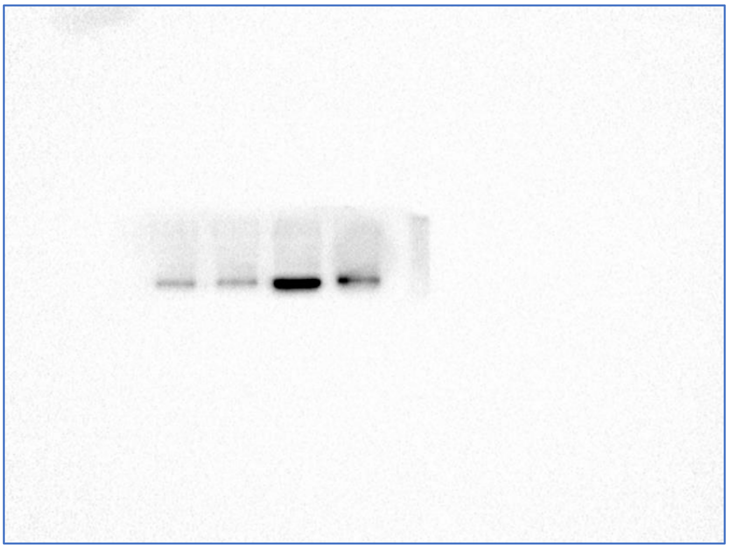

Fig. 6e – GAPDH - Repeat1

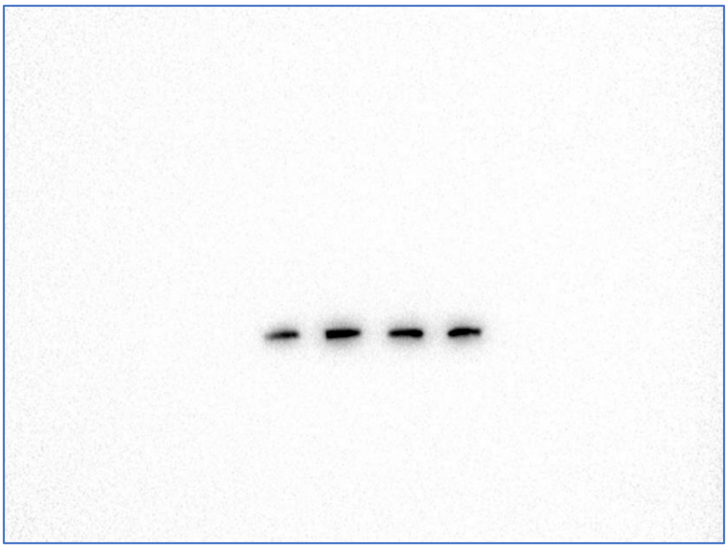

Fig. 6e – GAPDH – Repeat2

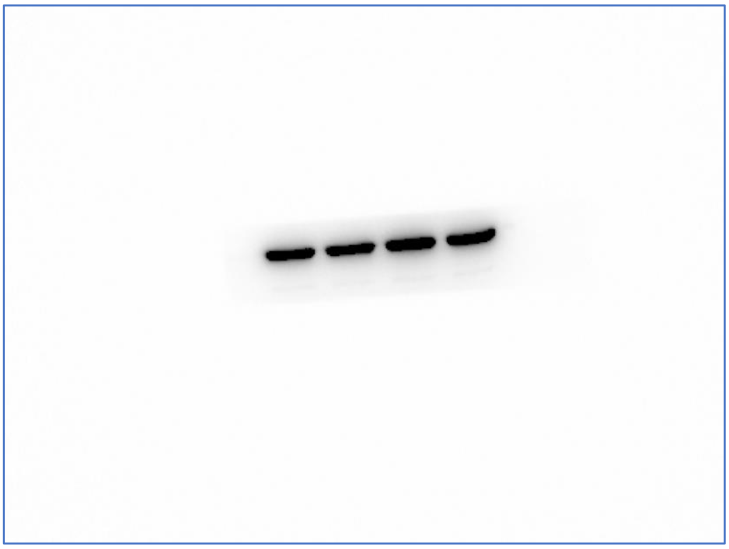

Fig. 6e – GAPDH – Repeat3

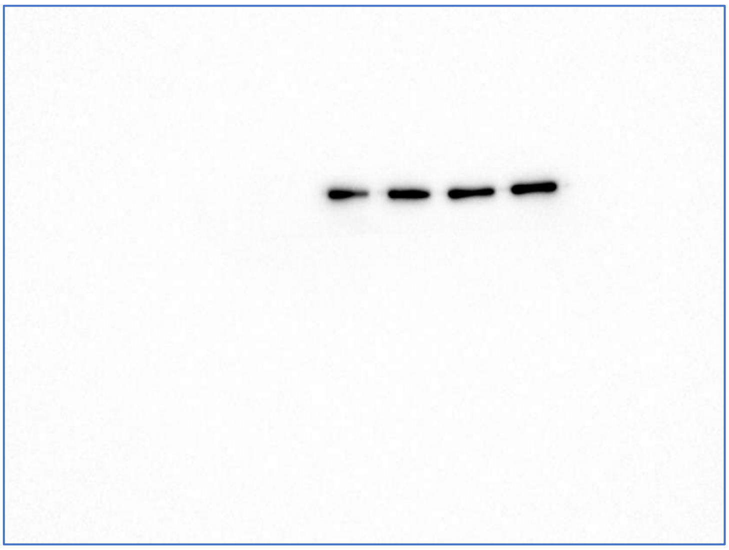

Fig.S1b-CDKN1A - Repeat1

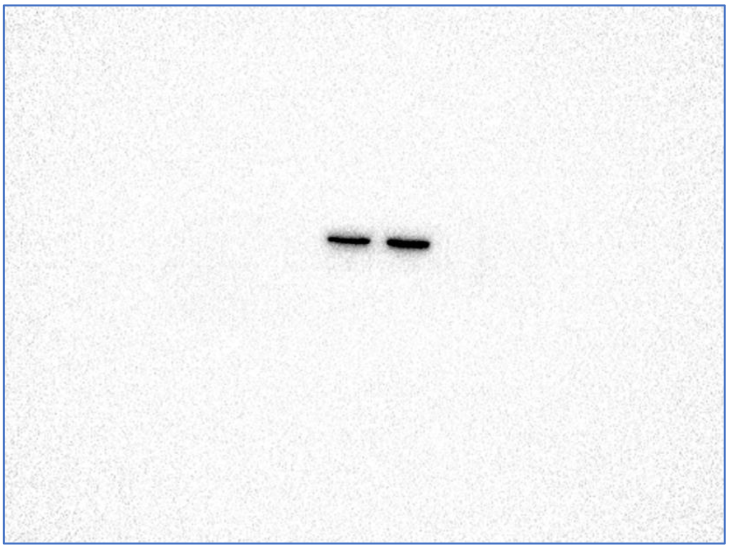

Fig.S1b-CDKN1A – Repeat2

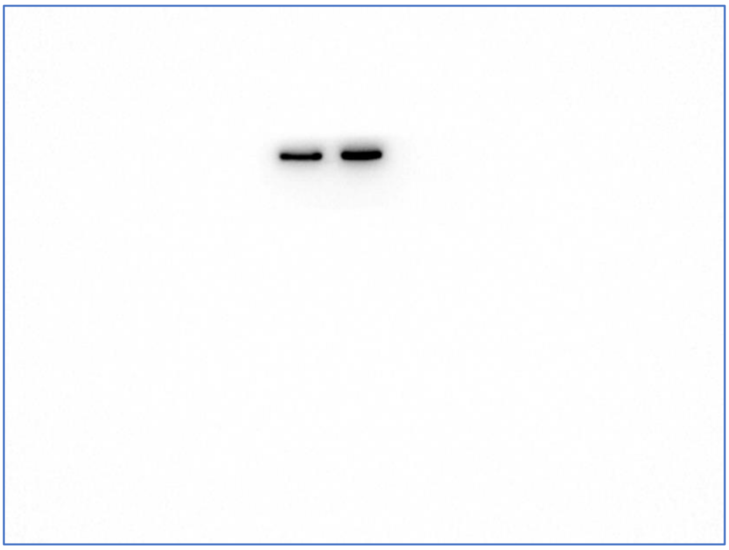

Fig.S1b-CDKN1A – Repeat3

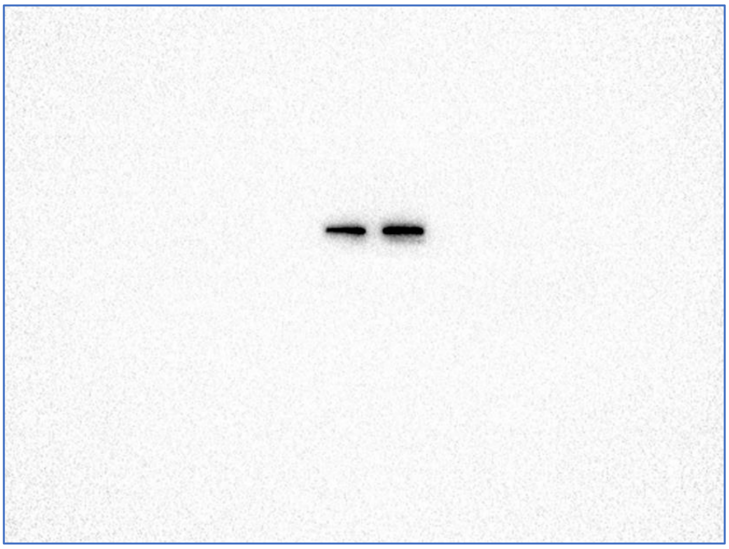

Fig.S1b-GAPDH - Repeat1

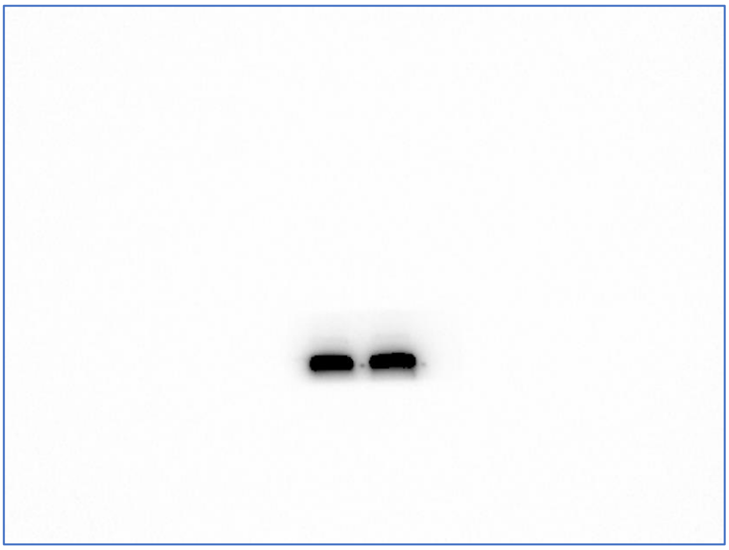

Fig.S1b-GAPDH – Repeat2

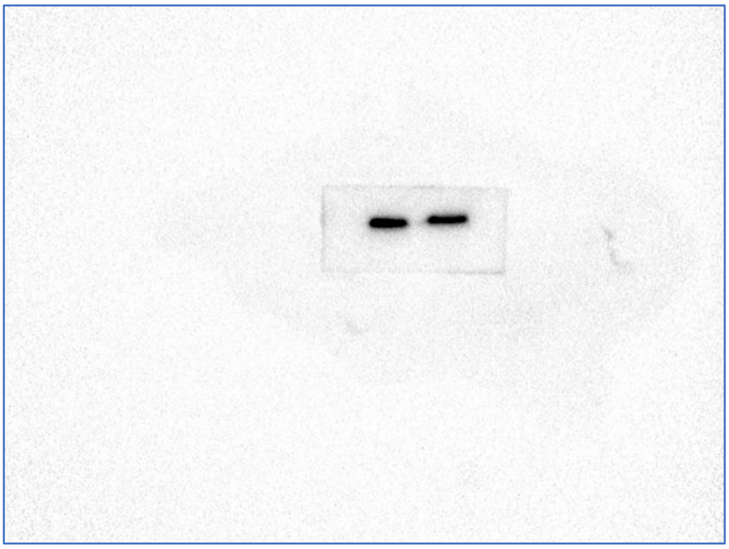

Fig.S1b-GAPDH – Repeat3

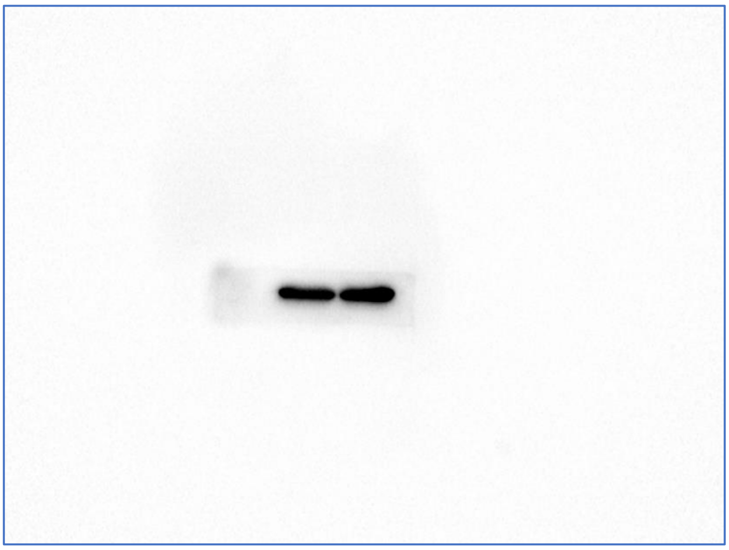

Fig.S1e-CDKN1A - Repeat1

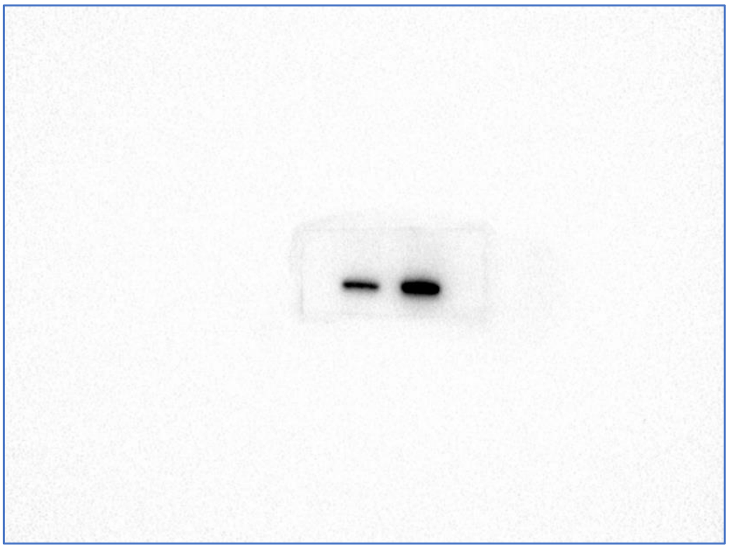

Fig.S1e-CDKN1A – Repeat2

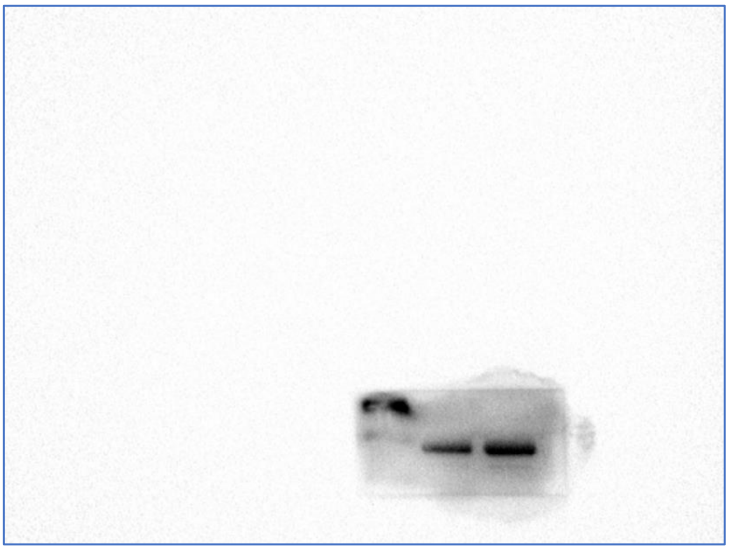

Fig.S1e-CDKN1A – Repeat3

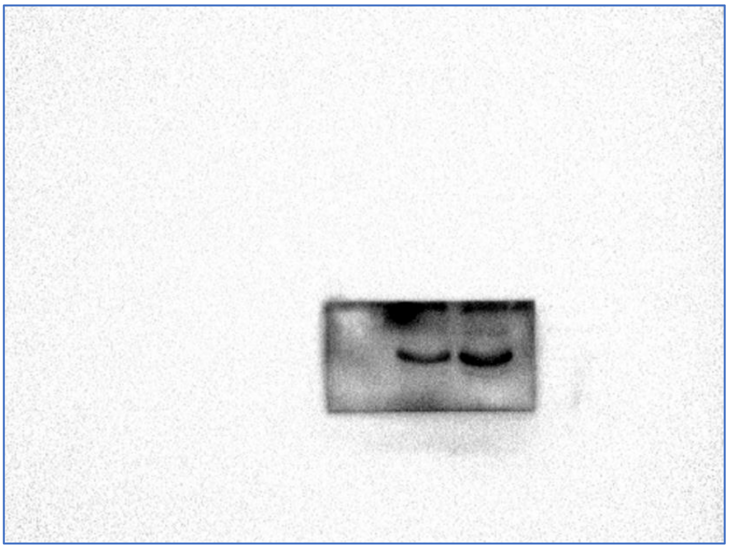

Fig.S1e-GAPDH - Repeat1

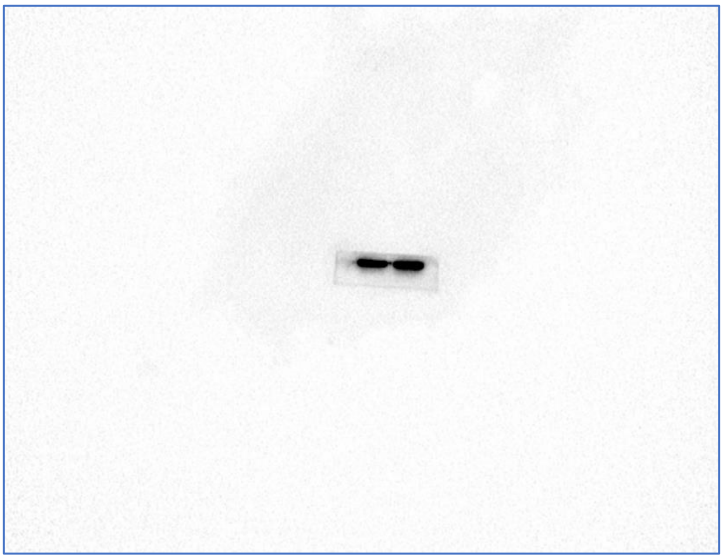

Fig.S1e-GAPDH – Repeat2

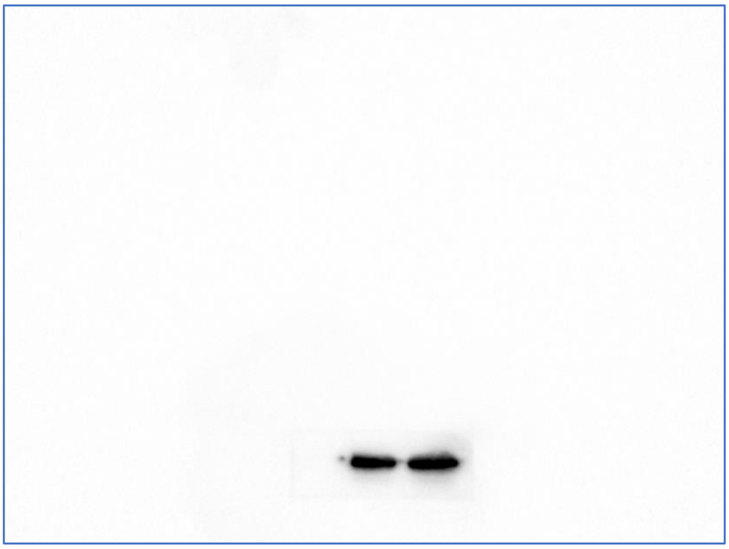

Fig.S1e-GAPDH – Repeat3

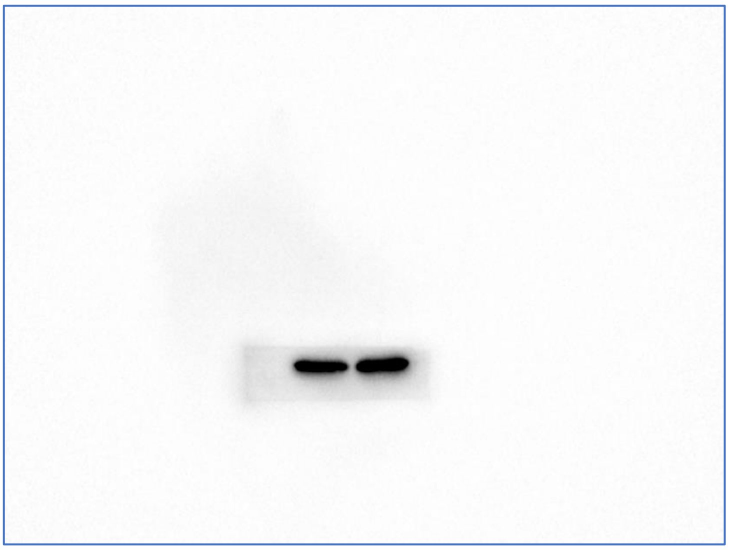

Fig.S1h-CDKN1A - Repeat1

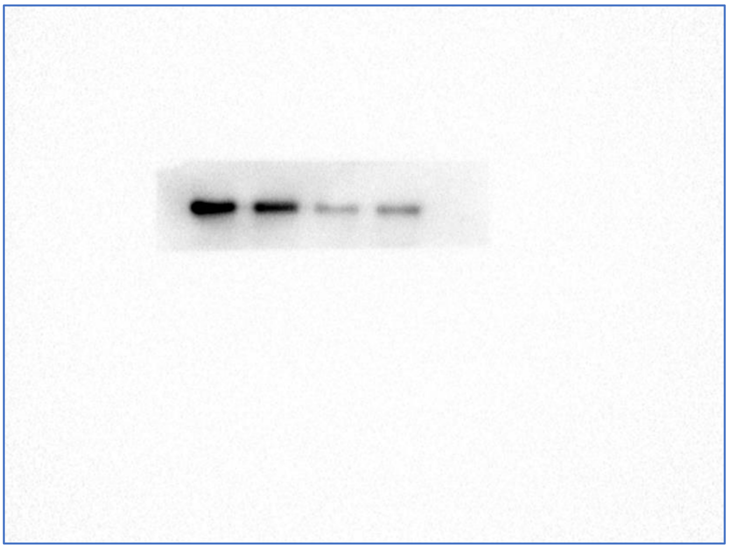

Fig.S1h-CDKN1A – Repeat2

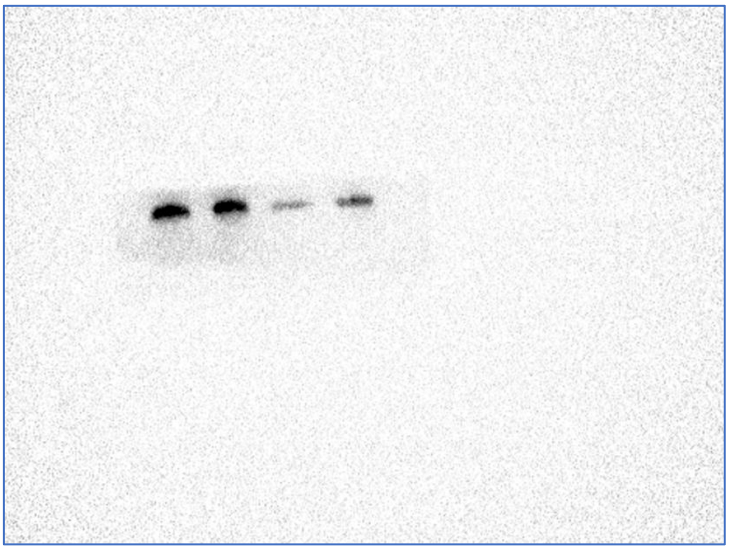

Fig.S1h-CDKN1A – Repeat3

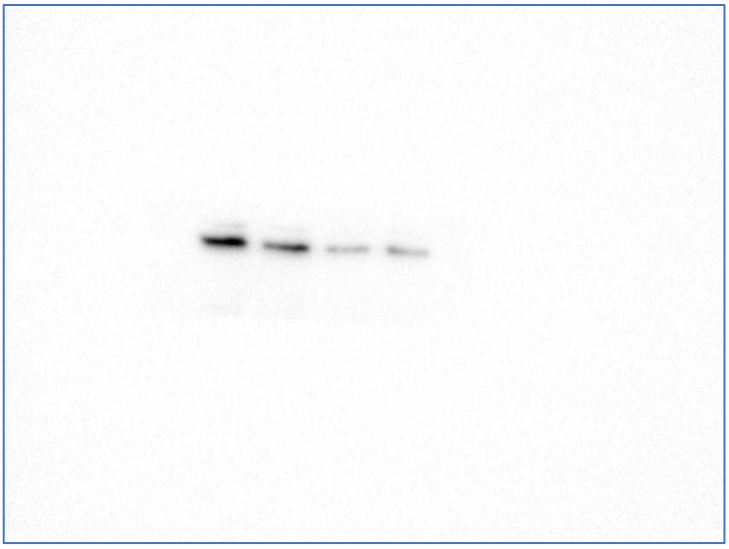

Fig.S1h-GAPDH - Repeat1

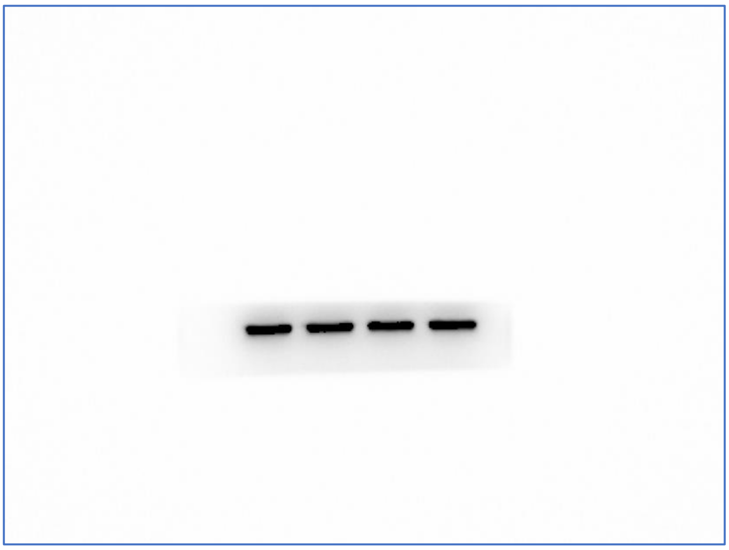

Fig.S1h-GAPDH – Repeat2

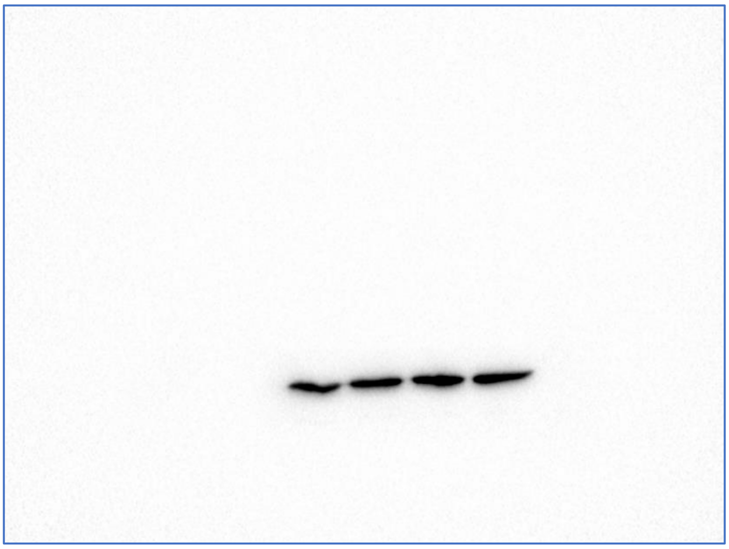

Fig.S1h-GAPDH – Repeat3

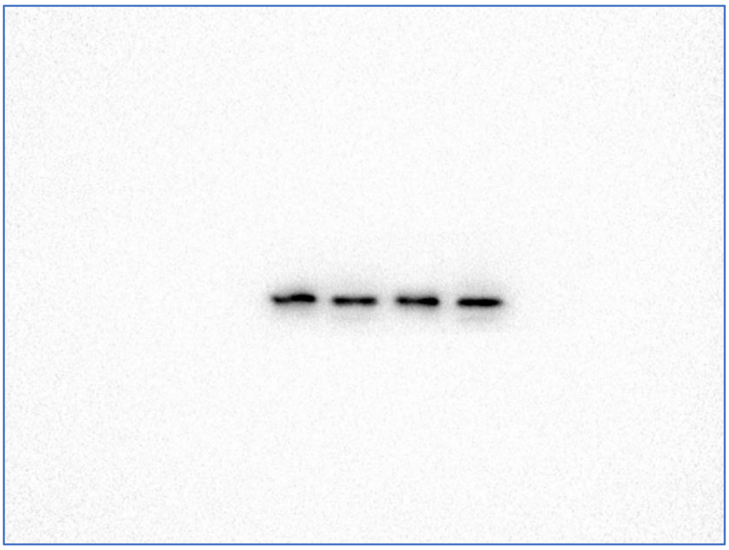

Fig.S3g-CASP-1 - Repeat1

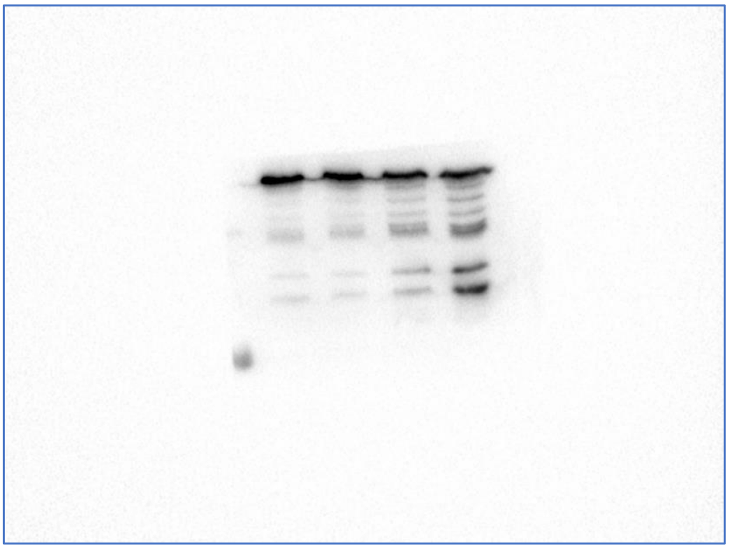

Fig.S3g-CASP-1 – Repeat2

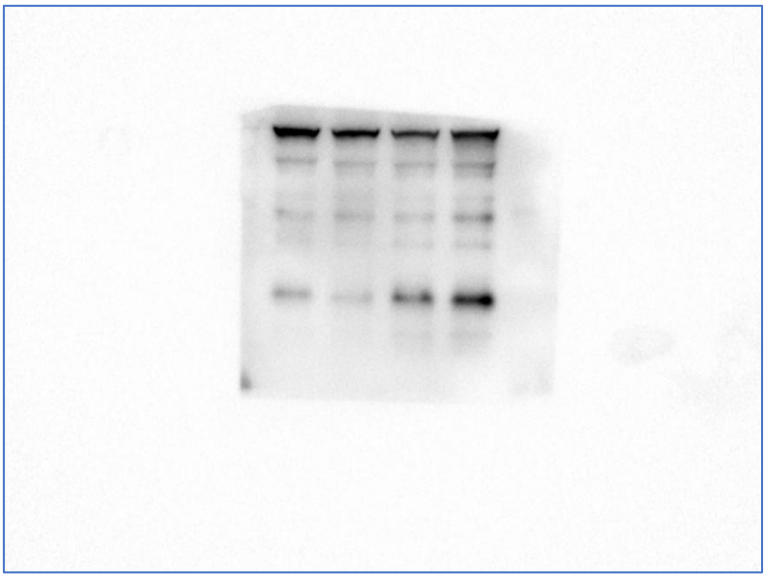

Fig.S3g-CASP-1 – Repeat3

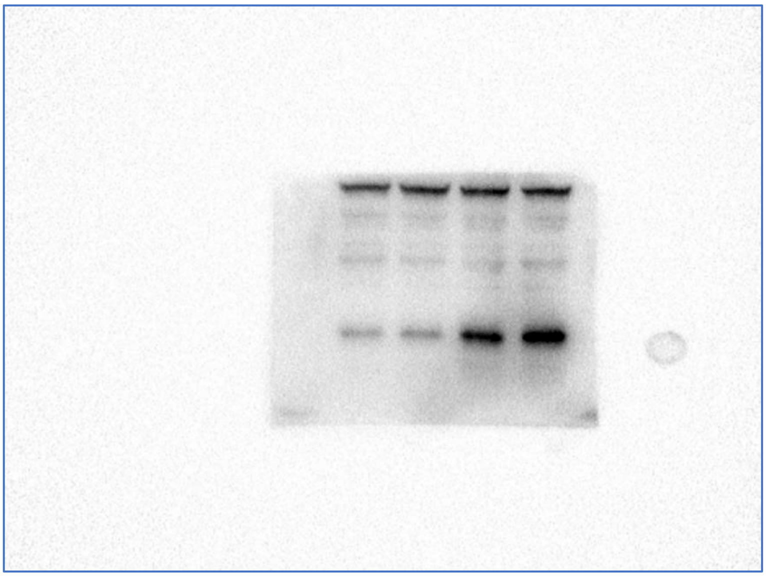

Fig.S3g-IL-18- Repeat1

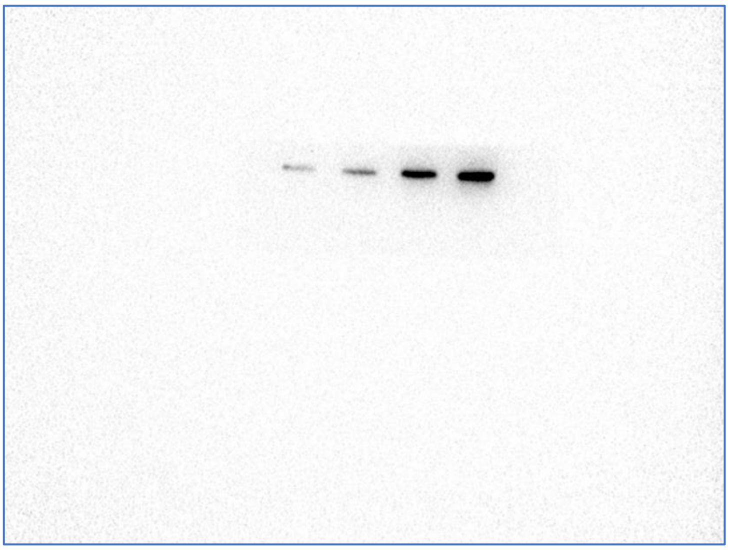

Fig.S3g-IL-18- Repeat2

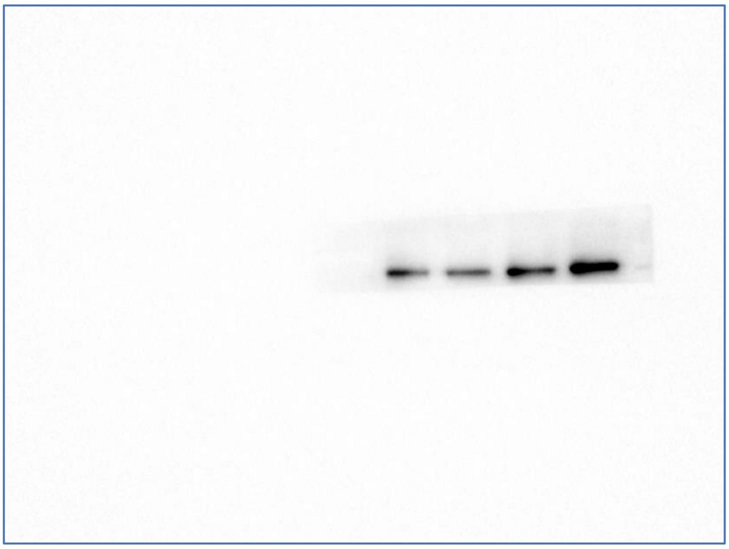

Fig.S3g-IL-18- Repeat3

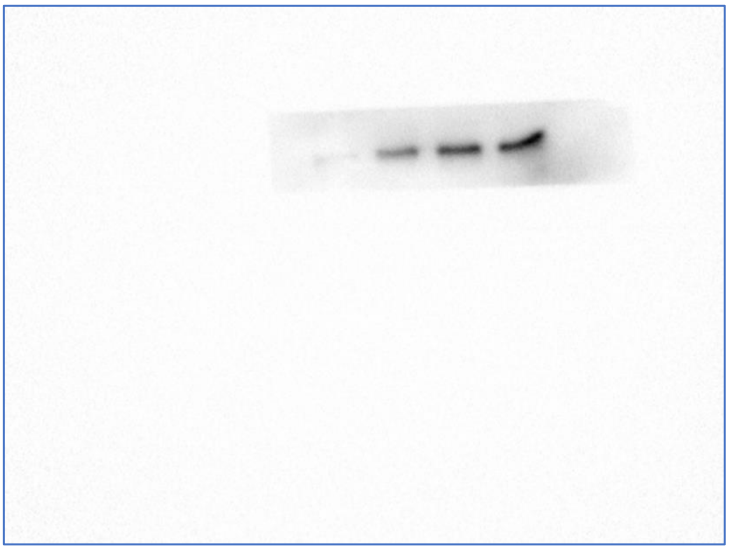

Fig.S3g-IL-1β- Repeat1

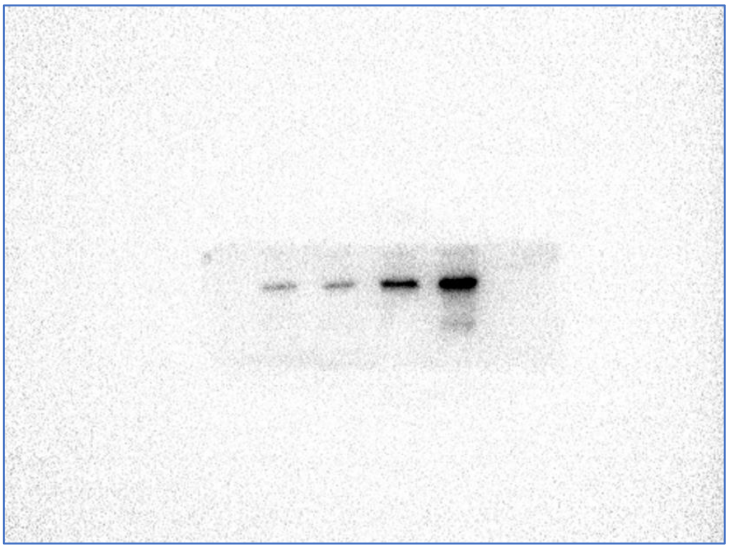

Fig.S3g-IL-1β- Repeat2

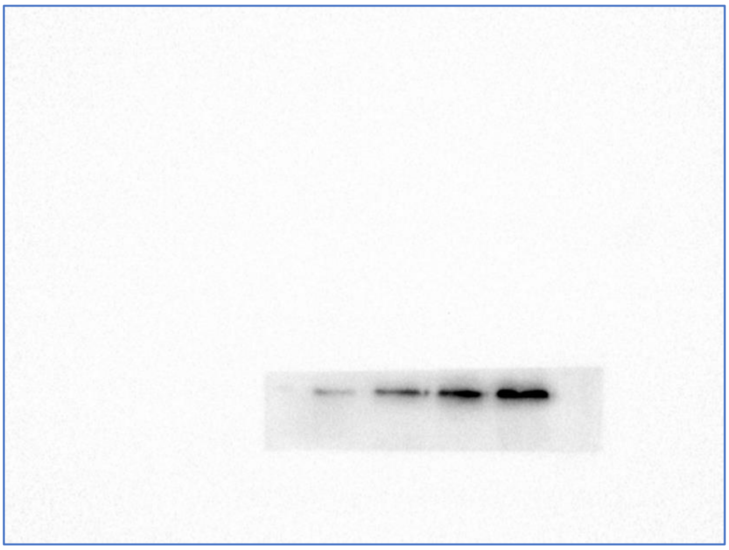

Fig.S3g-IL-1β- Repeat3

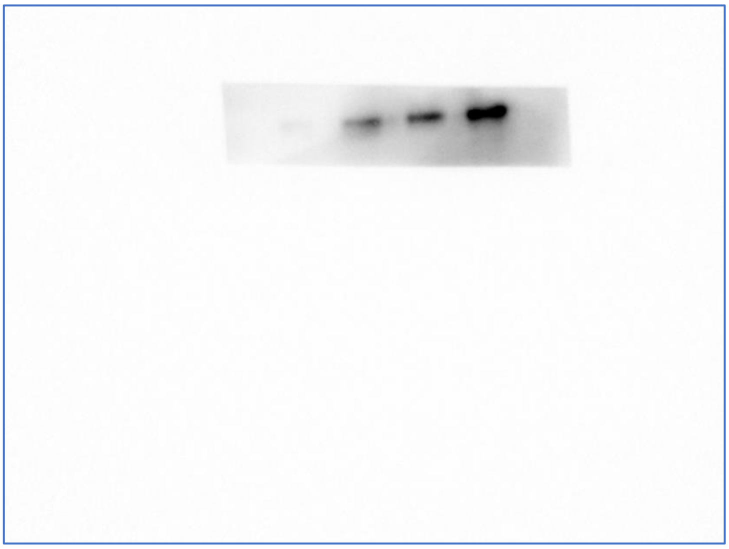

Fig.S3g-GAPDH - Repeat1

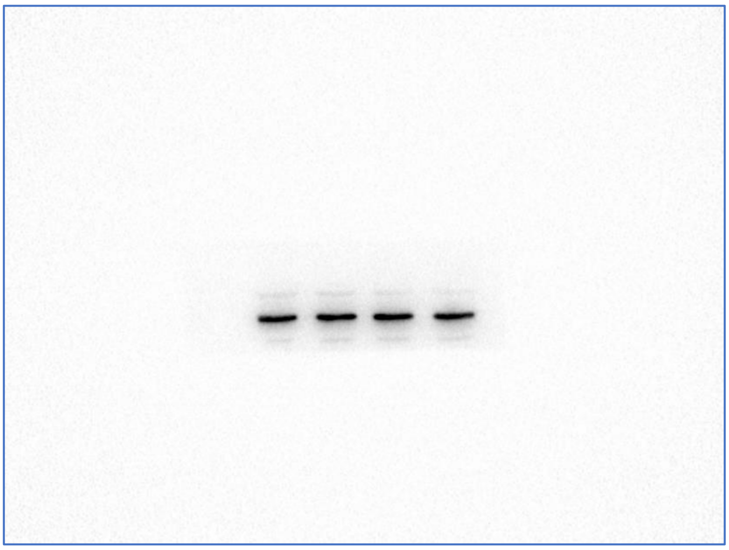

Fig.S3g-GAPDH – Repeat2

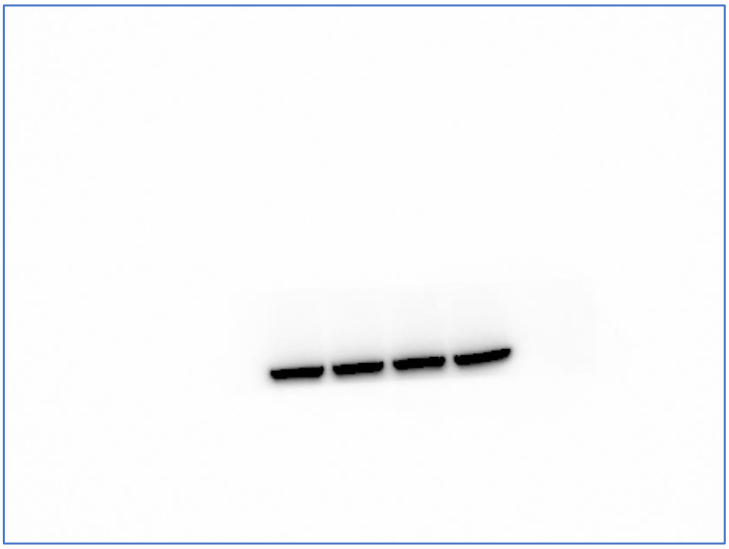

Fig.S3g-GAPDH – Repeat3

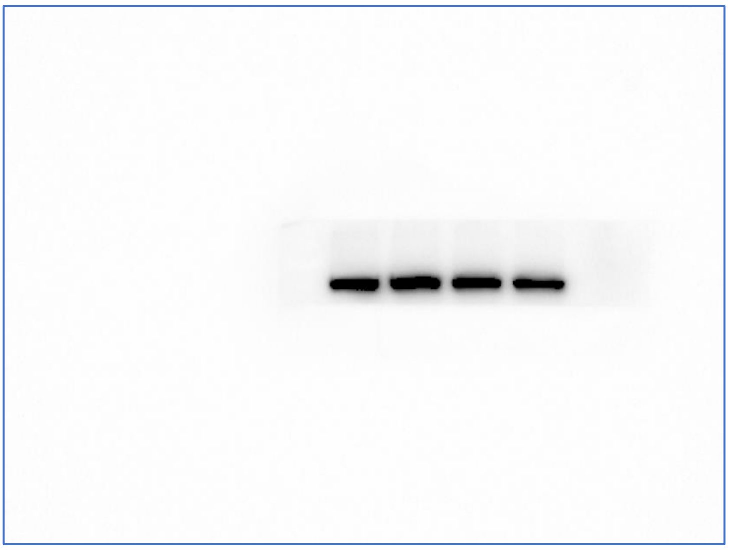

Supplement: Multimedia component 1 [file mmc1.pdf]
