# Supplementary material for: Cyclin-dependent kinase inhibitor 1A inhibits pyroptosis to enhance human lung adenocarcinoma cell radioresistance by promoting DNA repair
Source: Heliyon. 2024 Feb 29;10(5):e26975. doi: 10.1016/j.heliyon.2024.e26975 (PMC10926078; doi:10.1016/j.heliyon.2024.e26975)
Supplement: Multimedia component 2 [file mmc2.pdf]

Cyclin-Dependent Kinase Inhibitor 1A inhibits pyroptosis to enhance human lung adenocarcinoma cell radioresistance by promoting DNA repair

Supplementary Figures

Figure S1. CDKN1A promotes A549 cell survival after radiotherapy.

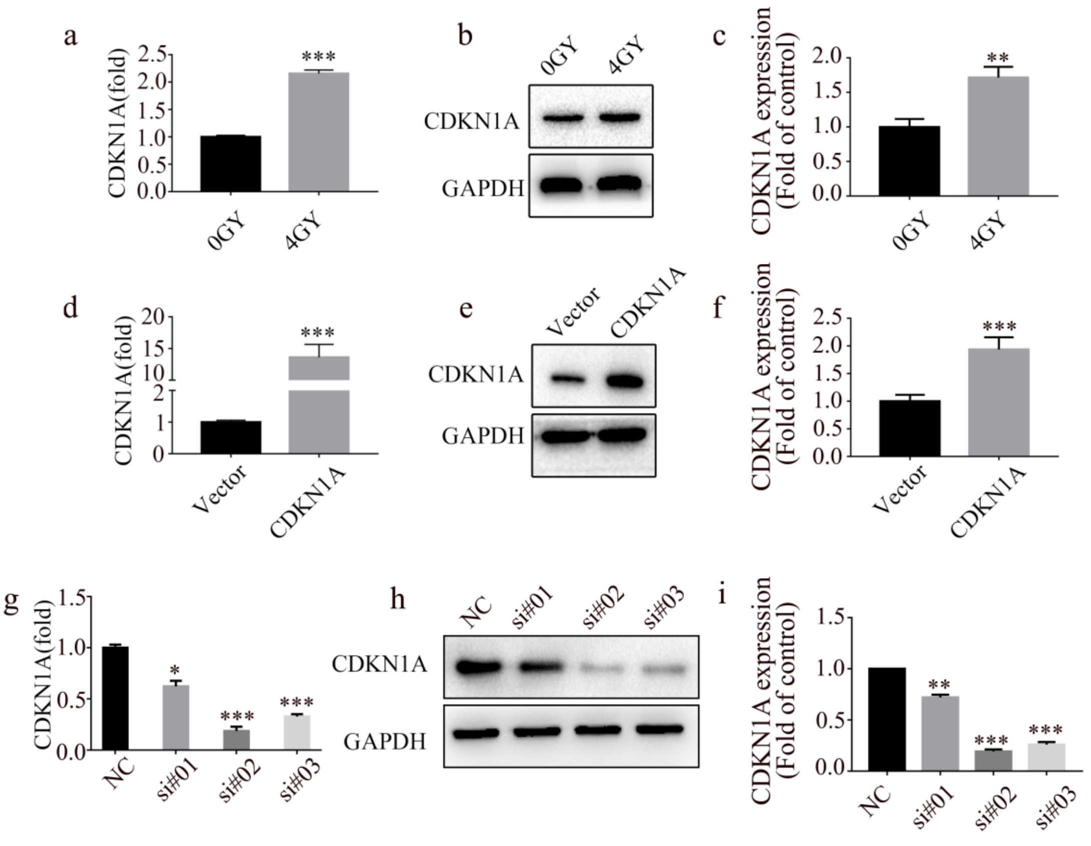

(a-c) Validation of CDKN1A expression by qPCR (a; mean ± SD, unpaired Student's t-test; $p < 0.001$, n = 3 independent experiments), WB (b) and the quantification graphs (c) after radiotherapy. (d-f) Validation of CDKN1A overexpression by qPCR (d; mean ± SD, unpaired Student's t-test; $p < 0.001$, n = 3 independent experiments) and WB (e) and the quantification graphs (f). (g-i) Validation of CDKN1A knockdown by qPCR (g; mean ± SD, unpaired Student's t-test; $p < 0.001$, n = 3 independent experiments) and WB (h) and the quantification graphs (i).

Figure S2. CDKN1A promotes H1650 cell survival after radiotherapy.

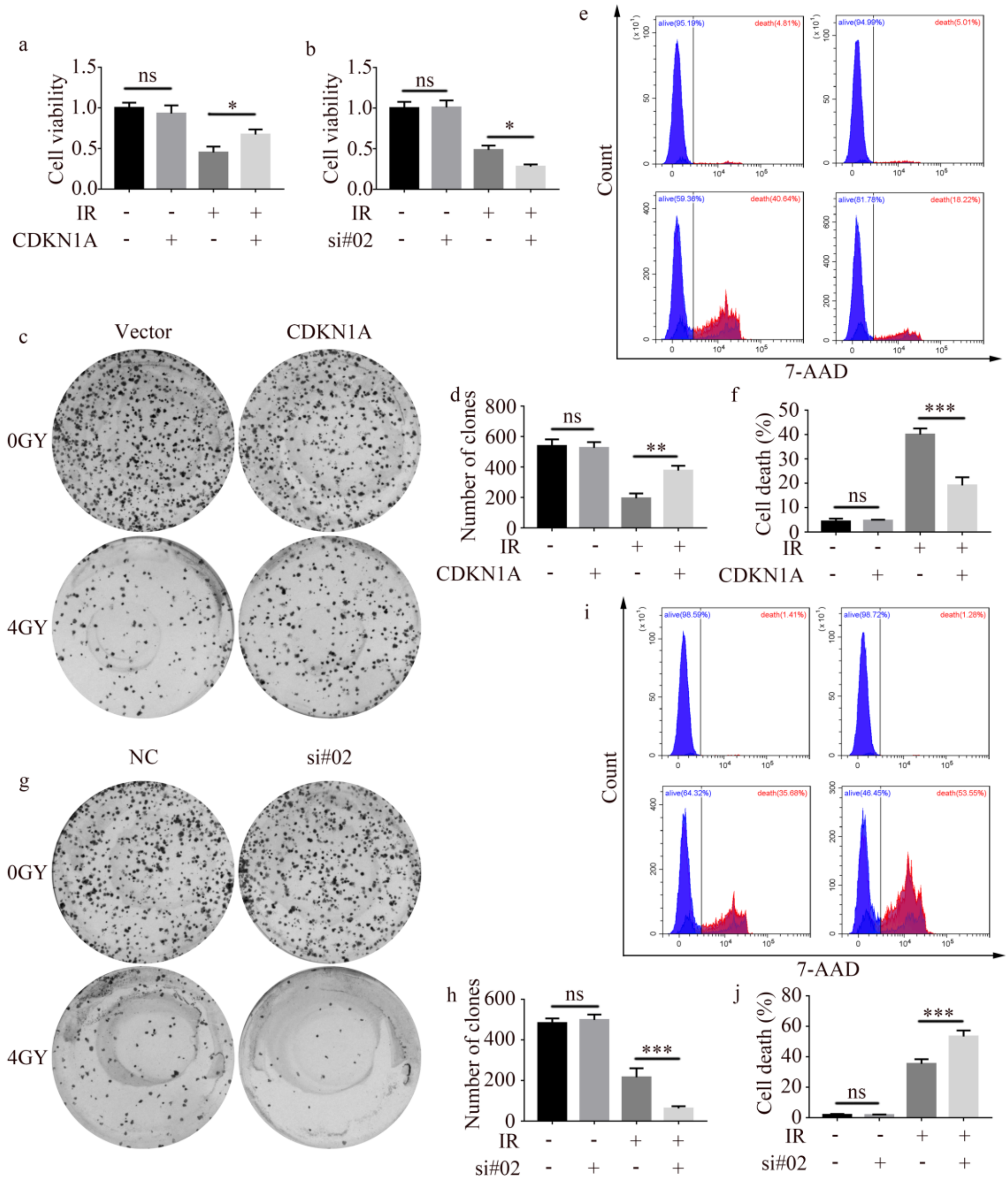

(a) H1650 cells were exposed to 4 Gy radiation after CDKN1A overexpression and cell viability was assessed using CCK-8 assay (mean ± SD, one-way ANOVA with Tukey's multiple comparison test; the third vs fourth group $p < 0.05$, $n = 3$ independent experiments). (b) H1650 cells were exposed to 4 Gy radiation after CDKN1A knockdown and cell viability was assessed using CCK-8 assay (mean ± SD, one-way ANOVA with Tukey's multiple comparison test; the third vs fourth group $p < 0.05$, $n = 3$ independent experiments). (c, d) Radiation-induced death of

H1650 cells with CDKN1A overexpression was monitored using colony formation assays. Representative images (c) and quantitation (d) are shown (mean ± SD, one-way ANOVA with Tukey's multiple comparison test; the third vs fourth group $p < 0.01$, $n = 3$ independent experiments). (e, f) Radiation-induced death of CDKN1A-overexpressing H1650 cells was detected via flow cytometry. Representative images (e) and quantitation (f) are shown (mean ± SD, one-way ANOVA with Tukey's multiple comparison test; the third vs fourth group $p < 0.001$, $n = 3$ independent experiments). (g, h) Radiation-induced death of CDKN1A-knockdown H1650 cells was monitored using colony formation assays. Representative images (g) and quantitation (h) are shown (mean ± SD, one-way ANOVA with Tukey's multiple comparison test; the third vs fourth group $p < 0.001$, $n = 3$ independent experiments). (i, j) Radiation-induced death of CDKN1A-knockdown H1650 cells was detected using flow cytometry. Representative images (i) and quantitation (j) are shown (mean ± SD, one-way ANOVA with Tukey's multiple comparison test; the third vs fourth group $p < 0.001$, $n = 3$ independent experiments).

Figure S3. CDKN1A knockdown after overexpression reverses the suppression of radiation-induced pyroptosis by CDKN1A in A549 cells.

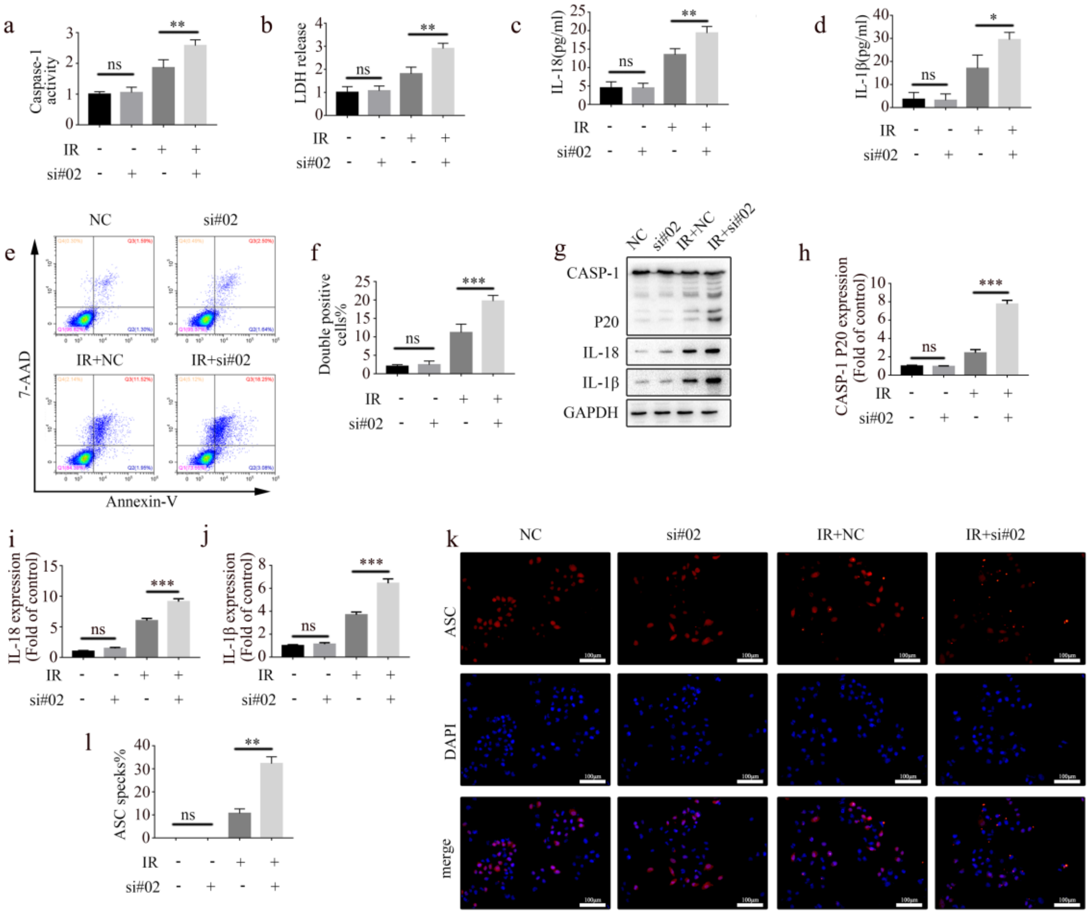

(a) Caspase-1 activity in A549 cells under different treatment conditions (mean ± SD, one-way ANOVA with Tukey's multiple comparison test; the third vs fourth group $p < 0.01$, n = 3 independent experiments). (b) Cell death assessment based on amount of LDH released into supernatant (mean ± SD, one-way ANOVA with Tukey's multiple comparison test; the third vs fourth group $p < 0.01$, n = 3 independent experiments). (c) IL-18 concentration in supernatant of A549 cultures under different treatment conditions (mean ± SD, one-way ANOVA with Tukey's multiple comparison test; the third vs fourth group $p < 0.01$, n = 3 independent experiments). (d) IL-1β concentration in supernatant of A549 cultures under different treatment conditions (mean ± SD, one-way ANOVA with Tukey's multiple comparison test; the third vs fourth group $p < 0.05$, n = 3 independent experiments). (e, f) Irradiation-induced death in A549 by 7AAD and Annexin V cytometry (e) and quantification graphs (f) are shown (mean ± SD, one-way ANOVA with Tukey's multiple comparison test; the third vs fourth group $p < 0.001$, n = 3 independent experiments). (g-j) Representative blots of caspase-1 p20, IL-18, and IL-1β expression in A549 determined by western blot (g) and the quantification graphs (h-j). (k, l) ASC specks detected by

immunofluorescence staining (k) and quantification graphs are shown (l) (mean ± SD, one-way ANOVA with Tukey's multiple comparison test; the third vs fourth group $p < 0.01$, $n = 3$ independent experiments). Scale bar = 100 μm.
